# Synthesis and biological evaluation of phosphatidylcholines with cinnamic and 3-methoxycinnamic acids with potent antiproliferative activity†

**DOI:** 10.1039/c8ra07002d

**Published:** 2018-10-19

**Authors:** Marta Czarnecka, Marta Świtalska, Joanna Wietrzyk, Gabriela Maciejewska, Anna Gliszczyńska

**Affiliations:** Department of Chemistry, Wrocław University of Environmental and Life Sciences Norwida 25 50-375 Wrocław Poland anna.gliszczynska@wp.pl marta.b.czarnecka@gmail.com; Department of Experimental Oncology, Ludwik Hirszfeld Institute of Immunology and Experimental Therapy, Polish Academy of Science Weigla 12 53-114 Wrocław Poland; Central Laboratory of the Instrumental Analysis, Wrocław University of Technology Wybrzeże Wyspiańskiego 27 50-370 Wrocław Poland

## Abstract

A series of eight novel phosphatidylcholines containing cinnamic or 3-methoxycinnamic acids (3a-b, 5a-b, 9a-b, 10a-b) at *sn*-1 and/or *sn*-2 positions were synthesized and tested for their antiproliferative activity in an *in vitro* model against representative six human cancer cell lines (MV4-11, A549, MCF-7, LoVo, LoVo/DX, HepG2) and a normal cell line BALB/3T3. The structures of the new compounds were confirmed by spectral analysis. Biological evaluation revealed that all the tested conjugates exhibited higher antitumor activity than the corresponding free aromatic acids. Compounds 3b and 9b turned out to be the most active, with IC_50_ values of 32.1 and 30.5 μM against the LoVo/DX and MV4-11 cell lines, respectively. Studies of the mechanism of the antitumor action were carried out for 1-palmitoyl-2-cinnamoyl-*sn*-glycero-3-phosphocholine (5a), and it was shown to be active toward almost all the tested types of cancer cells, showing that this compound could effectively arrest the cell cycle in G2/M and decrease the mitochondrial membrane potential of leukemia MV4-11 cells. The obtained results proved that the strategy of the incorporation of cinnamic and 3-methoxycinnamic acids into phospholipids could expand their potential application in industry, as well as could improve their antiproliferative activity and selectivity toward cancer cell lines.

## Abstract 2

A series of eight novel phosphatidylcholines containing CA or 3-OMe-CA acids (3a-b, 5a-b, 9a-b, 10a-b) at *sn*-1 and/or *sn*-2 positions were synthesized and tested for their antiproliferative activity against selected cancer cell lines.

## Graphical abstract

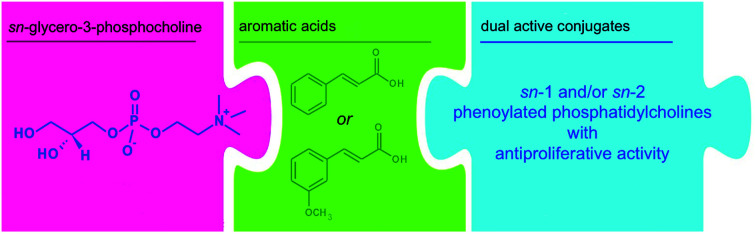

## Introduction

Cancer diseases are among the most significant health problems in the world, being the main cause of death. Chemotherapy is the basic method for cancer treatment; however, due to the fact that available chemotherapeutics also act on healthy cells, it is necessary to look for new anticancer agents, particularly those with a selective mechanism of action.

In the search for molecules that are non-toxic for humans and that have the potential to suppress tumor development and counteract cancers already developed, we became interested in cinnamic acid (CA) (1a) and its methoxy derivatives. These compounds have been known for centuries in treatments for cancer. The first mention of this subject came from 1905 and indicated that a 10% sodium cinnamate solution was a substance that could support anticancer therapy.^1^ Liu and co-workers proved that cinnamic acid has a cytostatic effect on human glioblastoma cells (A175, U251), melanoma (MEL 1011, A375(M), SKMEL 28), prostate cancer (PC3(M), Du145, LNCaP), and lung cancer (A549) at doses that have no significant effect on normal cells.^2^ The anticancer activity of CA (1a) has been reported to be a result of its inhibitory effect on 17β-hydroxysteroid dehydrogenase type 5 (AKR1C3), which indicated a potential use of this compound in the treatment of hormone-dependent forms of cancers.^3^ CA (1a) causes the cell cycle arrest of human cervical cancer cell lines (HeLa), malignant melanoma (Fem-x), and breast cancer (MCF-7) and induces apoptosis of human melanoma cells (HT-144).^4^ In studies of the mechanism of CA action on human leukemia cells (K562) it has been confirmed that this compound promotes cell cycle arrest by prolonging the G1/G0 phase and inducing cell apoptosis.^5^

Methoxy derivatives of cinnamic acid suppress benzo(*a*)pyrene-induced neoplasia of the forestomach and inhibit invasion and metastasis in the melanoma cell lines.^6,7^ In other studies, they turned out to be effective chemopreventive agents against 1,2-dimethylhydrazine in an *in vivo* model, which indicated their possible application in the prevention of colon carcinogenesis.^8^ For methoxy derivatives of cinnamic acid, induction of an intrinsic apoptosis pathway (dependent on mitochondria) in the human colon cancer cell line (HTC-116) has also been confirmed.^9^

Cinnamic acid (1a) and its methoxy derivatives are also known to exert a number of other beneficial effects. Among these, antimicrobial,^10,11^ hepatoprotective,^12^ and antidiabetic^13,14^ activities, as well as a protective effect against glutamate-induce neurodegeneration in cortical neurons, which should be especially emphasized.^15^

Despite extensive literature data indicating the biological activities of cinnamic acid and its methoxy derivatives, it is difficult to achieve in practice an anticancer and pro-health impact of these compounds on the body. The biological effects of aromatic acids delivery to the organisms from natural sources depend not only on their chemical form but also on the level of their release from the food matrix *via* gut microbes. It has been also confirmed that even when they are supplied in the free form to the organism, their bioavailability is still very low, because of their fast metabolism and elimination in both urine and bile after ingestion. Therefore, their effects that have been proved in *in vitro* studies are difficult to achieve in *in vivo* experiments. For this reason, in the food and pharmaceutical industries, the products of the lipophilization of aromatic acids are used. One of the most effective strategies to enhance their bioavailability in biological systems is through covalent bounding with phospholipids (PLs). Only a few papers concerning aromatic acids attached to PLs have been published so far. Yang and co-workers incorporated ferulic acid into the structure of phosphatidylcholine using lipase Novozym 435.^16^ In another study, phospholipids derivatives of syringic and vanillic acids obtained by chemical synthesis were introduced as new food-based ingredients with potential application in the food industry.^17^

Recently, we described phospholipid conjugates of methoxy derivatives of benzoic acid as potential anticancer chemotherapeutics.^18^ Herein, we report the synthesis of phosphatidylcholines containing in their structures cinnamic acid (CA) (1a) and 3-methoxycinnamic acid (3-OMe–CA) (1b), which are known to be even more active antitumor agents than the methoxy derivatives of benzoic acid, like anisic or veratric acids.

## Results and discussion

### Chemoenzymatic synthesis of phospholipid with cinnamic (1a) and 3-methoxycinnamic acid (1b) residues

**Scheme 1:**
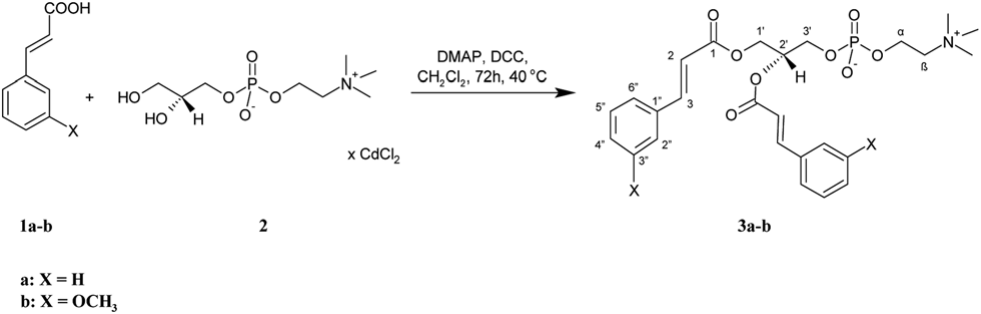
Synthesis of symmetrically substituted phosphocholines (3a-b) containing the CA and 3-OMe–CA residues.

**Scheme 2:**
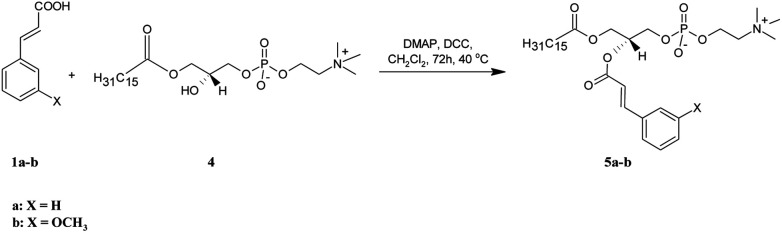
Synthesis of asymmetrically substituted phosphatidylcholines containing CA or 3-OMe–CA in the *sn*-2 position (5a-b).

**Scheme 3:**
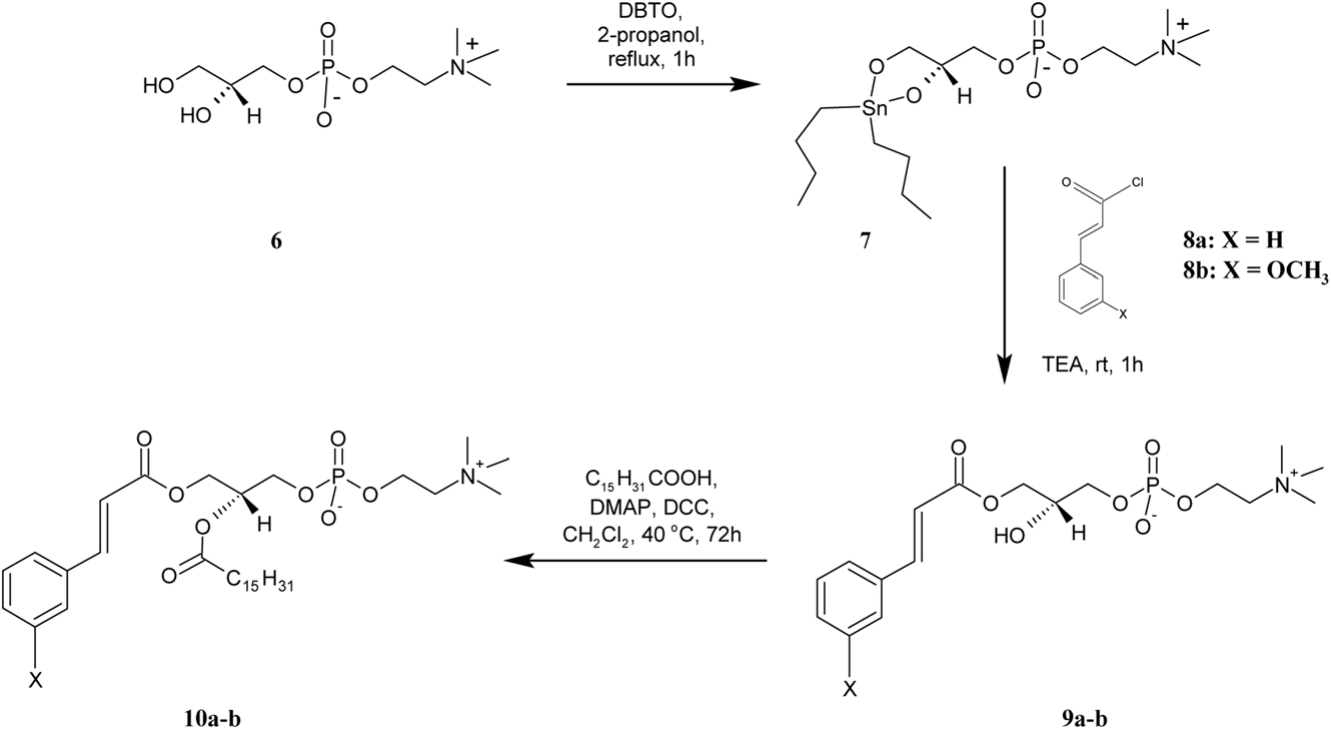
Synthesis of asymmetrically substituted phosphatidylcholines containing CA or 3-OMe–CA in the *sn*-1 position (10a-b).

All the target conjugates (3a-b, 5a-b, 7a-b, 8a-b) were synthesized as outlined in Scheme 1–3. First, symmetrically substituted phosphatidylcholines with cinnamic or 3-methoxycinnamic acid residues (3a-b) were prepared (Scheme 1). Cinnamic acid (CA) (1a) and 3-methoxycinnamic acid (3-OMe–CA) (1b) were esterified with the cadmium complex of *sn*-glycero-3-phosphocholine (GPC × CCl_2_) (2) in the presence of 4-(*N*,*N*-dimethylamino)pyridine (DMAP) as a catalyst and *N*,*N*′-dicyclohexylcarbodiimide (DCC) as a coupling agent. The products 3a-b were then purified by column chromatography. 1,2-Dicinnamoyl-*sn*-glycero-3-phosphocholine (3a) and 1,2-di(3-methoxycinnamoyl)-*sn*-glycero-3-phosphocholine (3b) were obtained in high yields, 93.5% and 94%, respectively. The structures of 3a and 3b were confirmed by ^1^H, ^13^C, and ^31^P nuclear magnetic resonance (NMR) spectroscopy. Correlation spectroscopy (COSY), heteronuclear single-quantum correlation spectroscopy (HSQC), and mass spectra (ESI-MS) were also applied for this purpose. In the ^1^H NMR spectra of 3a and 3b, not only were the signals characteristic for PC fragments visible, but also the signals from the aromatic acids incorporated into its structure. Signals of CH_2_-1′ from the glycerol skeleton at *δ* = 4.17 and 4.31 for 3a and *δ* = 4.19 and 4.33 for 3b were observed as a doublet of doublets and as multiplets, respectively. The multiplets of H-2′ were observed in the spectra of 3a and 3b at characteristic chemical shifts of 5.18 and 5.21 ppm for *sn*-2 substituted phosphocholines, respectively. The signals at 2.96 and 2.99 ppm from three *N*-methyl groups from the choline were visible as singlets in the spectra of 3a and 3b. Characteristic signals of protons of aromatic moiety were found in the range 6.69–7.29 ppm. In the spectrum of 3a, the peaks at 6.22 and 7.45 ppm, as well as in the spectrum of 3b the peaks at 6.21 and 7.41 ppm, were from the olefinic protons. In the ^13^C NMR spectra of 3a and 3b, the carbon atoms of the α,β-double bond were detected at *δ* = 116.56, 116.69, 145.46, 145.64 and 116.80, 116.91, 145.39, 145.64, respectively. The signals of the ester carbon atoms were observed at 166.17 and 166.56 ppm and confirmed the presence of ester connections between aromatic acids and glycerol. The presence of an aromatic ring was also confirmed by the signals at 112.61–134.89 ppm observed in the ^13^C NMR spectra of 3a and 3b. The ^13^P NMR data confirmed that the phosphatidyl part in new molecules was retained, while the ESI-high-resolution mass spectra (HRMS) showed the expected mass values.

Two asymmetrically substituted phosphatidylcholines containing CA or 3-OMe–CA at the *sn*-2 position (5a-b) were obtained according to the reaction presented in Scheme 2. The starting lysophosphatidylcholine (PA-LPC) 4 was synthesized using phospholipase A_2_ (PLA_2_), as was described previously^19^ and was then subjected to esterification with 1a and 1b in the presence of DMAP and DCC. After purification, we received products 5a in a 90% yield and 5b in a 58% yield. The formation of 5a-b was confirmed by the ESI-MS spectra, in which intensive signals at *m*/*z* 626.3824 for 5a and 656.3925 for 5b were detected. To determine their structures, 1D and 2D NMR experiments were performed (all data are presented in the Experimental section and all the spectra are in the ESI†). In the NMR spectra of 5a-b, all characteristic signals from the glycerol, choline, aromatic acid, and palmitic acid were identified. In the ^1^H NMR spectra of 5a and 5b, signals from protons of the benzene ring (6.72–7.31 ppm) and olefinic protons (*δ* = 6.23 and 7.47 for 5a and 6.20 and 7.42 ppm for 5b) were visible. The terminal methyl signal of palmitoyl residue at the *sn*-1 position was observed at *δ* = 0.64 (5a) and 0.62 (5b) as a triplet (*J* = 6.8 Hz), whereas the signals for methylene groups in the palmitic acid residue were observed in the range 0.95–1.09 ppm. The chemical shift of the multiplet of H-2′ at 5.12 ppm in the spectra of 5a and 5b proved that the *sn*-2 position was esterified and PA-LPC was conjugated with aromatic acids. In the ^13^C NMR spectra of 5a and 5b, two carbon atom signals from ester groups were identified at *δ* = 166.13 and 173.63 for phosphocholine 5a and at *δ* = 166.26 and 173.75 for compound 5b. Signals of C-2 and C-3 carbon atoms were observed at 116.74 and 145.60 ppm for 5a and at 116.88 and 145.72 ppm for 5b. The ^31^P NMR data confirmed the presence of a phosphocholine 5a and 5b as a singlet at −0.66 and −4.64 ppm, respectively.

For the preparation of lyso PC 9a-b, containing the CA and 3-OMe–CA in the *sn*-1 position, GPC was regioselectively acylated using a tin-mediated mono-functionalization method described by D'Arrigo previously.^19^ First, the stanylene acetal 7 was prepared in the reaction of *sn*-glycerophosphocholine 6 with dibutyltin oxide (DBTO) and then subjected to acylation by cinnamoyl and 3-methoxycinnamoyl chlorides 8a-b obtained *in situ*, according to the method proposed by Mattson.^20^ Lysophosphocholines 9a-b were obtained in good yields of 67% and 69%, respectively. In the ^1^H NMR spectra of 9a-b, the characteristic for the –N^+^(CH_3_)_3_ group single peak at 3.01 ppm was detected. The multiplets of protons H-2′ in the range 3.73–3.85 ppm proved that the *sn*-2 position was non-esterified. The peaks from protons of the aromatic ring appeared in the range 6.83–7.31 ppm and confirmed the conjugation of CA and 3-OMe–CA with GPC. The structures of products 9a-b were fully confirmed also by the ^13^C, ^31^P, correlation spectroscopy and ESI-MS spectra as well.

The heterosubstituted phospholipids 10a-b, in which CA and 3-OMe–CA occur in the *sn*-2 position and the *sn*-1 position is occupied by the palmitic acid, were synthesized from 9a-b. These lyso PCs were subjected to Steglich esterification with palmitic acid. Phosphocholines 10a-b were obtained in high yields of 90% and 85.5%, respectively. The ^1^H NMR spectra of 10a-b displayed all the proton signals from CA/3-OMe–CA acid and GPC, such as signals for the hydrogen protons in aromatic rings at 6.72–7.36 ppm and the –N^+^(CH_3_)_3_ group at 2.98–3.01 ppm of the GPC fragment. The appearance of new peaks in the range 0.63–2.15 ppm confirmed successfully the incorporation of palmitic acid into the structure of the lyso PC used as a substrate. The ^1^H NMR spectra of 10a and 10b showed a multiplet of proton H-2′ at *δ* = 5.10 and 5.07, respectively. In comparison to the lysophosphatidylcholines 9a-b, the chemical shifts of these signals were shifted to a lower frequency, which proved that the *sn*-2 position was esterified. The structural assignments were also accomplished through extensive 2D NMR spectroscopy, and mass spectra (ESI-MS).

### Antiproliferative activity *in vitro* toward selected cancer cell lines

Cinnamic acid (1a), 3-methoxycinnamic acid (1b), and all the synthesized phosphatidylcholines (3a-b, 5a-b, 9a-b, 10a-b) were assessed for their antiproliferative activity against a panel of six cancer cell lines: MV4-11 (leukaemia), A549 (lung cancer), MCF-7 (breast cancer), LoVo (colon cancer), LoVo/DX (doxorubicin-resistant colon cancer), HepG2 (liver cancer), and normal mice fibroblast cells BALB/3T3. The selection of cancer cell lines was based on the literature data indicating the antiproliferative activity of benzoic and cinnamic acids and their methoxy derivatives.^2,4,5,8,9^ Biological studies were carried out using the 3-(4,5-dimethylthiazol-2-yl)-2,5-diphenyltetrazolium bromide (MTT) or sulforhodamine B (SRB) assays. Two commercial anticancer drugs, *i.e.*, cisplatin and doxorubicin, were used as the positive control. The results are shown in Table 1. Almost all the tested conjugates showed much stronger antiproliferative activity than free aromatic acids (which was statistically significant in comparison to the CA or 3-OMe–CA acids, *p* < 0.05). Except for the HepG2 line, asymmetrically substituted phosphatidylcholines 5a, 5b, and 10a were significantly more toxic toward the studied cancer lines than normal mice fibroblast. Among the tested compounds, 1-palmitoyl-2-cinnamoyl-*sn*-3-glycero-phosphocholine 5a appeared to be the most effective and promising chemoprevention agent active toward the wide range of studied cancer lines. Analyzing the correlations between the activity of the mentioned derivatives and their chemical structures, the position of the aromatic fragment in the skeleton of glycerol seemed to be significant. Higher cytotoxic activities exhibited asymmetrically substituted phosphatidylcholines containing CA or 3-OMe–CA in the *sn*-2 position than those with aromatic acids in the *sn*-1 position. Lysophosphatidylcholines 9a and 9b were characterized by their selectivity of action and they effectively inhibited only the proliferation of leukaemia cells. Their determined IC_50_ values were respectively 8- and 11-fold lower than those reported for free cinnamic and 3-methoxycinnamic acids, being at the same time definitely less toxic toward normal cells. It is also worth noting that in the group of symmetrically substituted phosphatidycholines, 1,2-di(3-methoxycinnamoyl)-*sn*-glycero-3-phosphocholine (3b) was the most active derivative toward the doxorubicin-resistant line of colon cancer. This compound was effective in a concentration of 32.1 μM against LoVo/DX, while free acid 1b was not active at the tested range of concentrations (IC_50_ > 625 μM).

**Table 1:** Antiproliferative activity of the synthesized phospholipids against selected cell lines^a^

Compound	Acyl residue		Cell lines IC_50_ [μM]						
	*sn*-1	*sn*-2	MV4-11	A-549	MCF-7	LoVo	LoVo/DX	HepG2	BALB/3T3
1a	—	—	358.7 ± 104.6	>625	480.3 ± 46.2	283.6 ± 26.6	>625	>625	>625
3a	CA	CA	214.9 ± 51.9	>625	320.7 ± 11.1^b^	199.9 ± 49.7	329.2 ± 12.5	>625	>625
5a	PA	CA	**43.2 ± 9.9** b	**56.3 ± 0.4**	**64.6 ± 3.4** b	**52.3 ± 5** b	**56.3 ± 0.8**	227.6 ± 30.6	113.7 ± 36.6
9a	CA	—	**44.3 ± 5.1** b	287.1 ± 2	104.1 ± 4.4^b^	111.8 ± 26.3^b^	238.2 ± 2.3	289.7 ± 19.4	160 ± 45.7
10a	CA	PA	**88.7 ± 28.9** b	**61.9 ± 3.7**	126.9 ± 28.3^b^	**63 ± 14.4** b	**66 ± 8.7**	283.4 ± 5.2	207.6 ± 30.7
1b	—	—	338.7 ± 111.0	>625	420.2 ± 56.2	232.7 ± 15.5	>625	>625	506 ± 67.7
3b	3-OMe–CA	3-OMe–CA	165.3 ± 25.4	286.1 ± 5.3	307.3 ± 12.5^b^	209.9 ± 22.2	**32.1 ± 16.9**	83.6 ± 45.1	297.1 ± 56.4^b^
5b	PA	3-OMe–CA	**83.2 ± 4.0** b	192.6 ± 27.4	224.2 ± 9.5^b^	**70.4 ± 5.4** b	**69.9 ± 6.3**	294.8 ± 11.4	257.4 ± 52.4^b^
9b	3-OMe–CA	—	**30.5 ± 8.2** b	258.6 ± 16.7	84.1 ± 7.4^b^	85.1 ± 11.9^b^	160 ± 61.7	272.3 ± 33.1	154.3 ± 66.4^b^
10b	3-OMe–CA	PA	235.9 ± 46.3	280.4 ± 8.8	214.1 ± 89.6	211.6 ± 1.3	254.5 ± 11.6	296.5 ± 13.2	291.3 ± 5.6^b^
Cisplatin			1.3 ± 0.47	8.6 ± 0.7	8.1 ± 0.03	2.56 ± 0.4	3.17 ± 0.2	2.38 ± 0.64	4.2 ± 1.1
Doxorubicin				—	—	0.117 ± 0.012	6.53 ± 0.93	—	—

a IC_50_ – compound concentration leading to 50% inhibition of cell proliferation. Data are presented as the mean ± standard deviation (SD) calculated using Prolab-3 system based on Cheburator 0.4 software.^21^ Statistical analysis was performed using STATISTICA version 10 (StatSoft Inc., USA). *t*-test was used in the analysis.

b Results within column that are significantly different in comparison to CA or 3-OMe–CA, respectively; *p* < 0.05.

A comparison of the activities of phospholipid derivatives of CA and 3-OMe–CA showed that homosubstituted PC with 3-OMe–CA 3b was a little more active than homosubstituted PC with CA 3a. Moreover, for heterosubstituted PC with CA 5a and 10a and heterosubstituted PC with 3-OMe–CA 5b and 10b, the opposite correlations were observed. Phosphatidylcholines 5a and 10a were significantly more active than 5b and 10b.

The results of the antiproliferative activity of the previously reported phosphatidylcholines containing anisic/veratric acids^18^ and those with cinnamic/3-methoxycinnamic acids presented here confirmed that some carcinoma cell lines, such as MV4-11, MCF-7, and LoVo, seemed to be more sensitive to the studied conjugates than the other ones, like HepG2. Based on our results, it can be concluded that phospholipids with *O*-methylated derivatives of benzoic acid are more active in the form of 1-acyl-LPC than phosphatidylcholines with CA and 3-OMe–CA residues mainly in the form of heterosubstituted PC. However, it was difficult to determine the impact of the presence and the position of methoxy group in the benzene ring on the activity of the novel phospholipids. For this purpose, more PC derivatives should be synthesized and tested.

The phospholipid derivatives of cinnamic and 3-methoxycinnamic acids had also high antiproliferative activity against the doxorubicin-resistant LoVo/DX cell line. Resistance index (RI) values were calculated and the data are presented in Table 2. All of the obtained phosphatidylcholines were able to overcome drug resistance, especially 3b, and only 9a had a moderate ability to overcome drug resistance.

**Table 2:** Resistance index (RI) values of phospholipids with CA or 3-OMe–CA residues against a doxorubicin-resistant LoVo/DX cell line

Compound	Acyl residue		RI
	*sn*-1	*sn*-2	
1a	—	—	—
3a	CA	CA	1.65
5a	PA	CA	1.08
9a	CA	—	2.13
10a	CA	PA	1.05
1b	—	—	—
3b	3-OMe–CA	3-OMe–CA	0.15
5b	PA	3-OMe–CA	0.99
9b	3-OMe–CA	—	1.88
10b	3-OMe–CA	PA	1.2
DOX	—	—	55.81

### The effect of compound 5a on the cell cycle of the MV4-11 cells

**Fig. 1:**
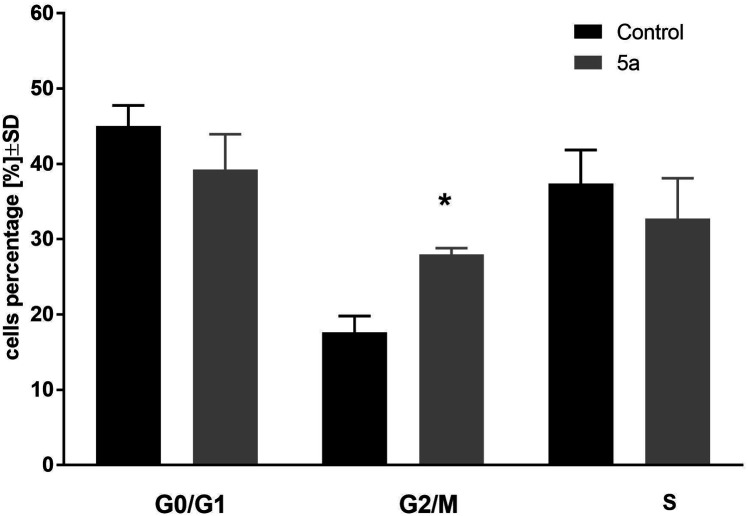
Cell cycle analysis of MV4-11 cells after treatment of 1-palmitoyl-2-cinnamoyl-*sn*-glycero-3-phosphocholine (5a; 50 μM); *p < 0.05 in comparison to control cells, *t*-test, Statistica v.10.

In the next step of the study, the most active compound 1-palmitoyl-2-cinnamoyl-*sn*-glycero-3-phosphocholine (5a) was chosen. The cell cycle of leukemia MV4-11 cells was analyzed after 72 h treatment of compound 5a in the concentration 50 μM (Fig. 1).

This compound arrested cell cycle in the G2/M phase (which was statistically significant in comparison to the control cells, *p* < 0.05) and lowered the percentage of cells in G0/G1 and S phase (which was statistically not significant in comparison to the control cells).

### The effect of derivative 5a on the mitochondrial membrane potential and cell death of the MV4-11 cells

**Fig. 2:**
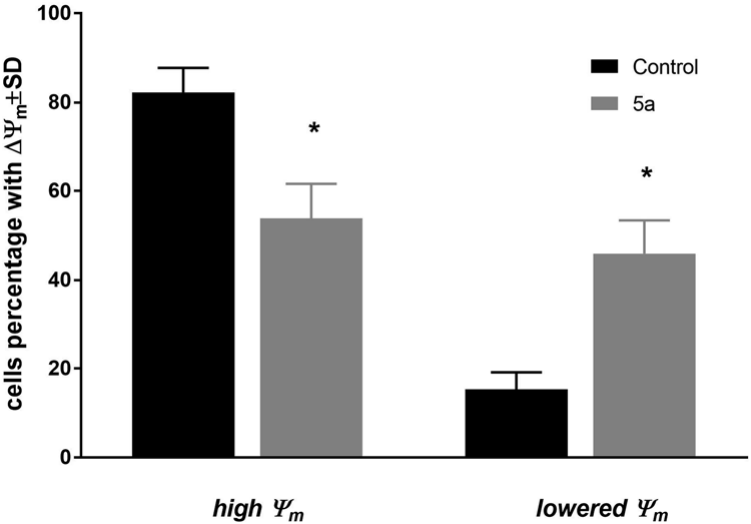
Mitochondrial membrane potential (Δ*Ψ*_m_) of MV4-11 cells after treatment of 1-palmitoyl-2-cinnamoyl-*sn*-glycero-3-phosphocholine (5a; 50 μM).

The changes of mitochondrial membrane potential (Δ*Ψ*_m_) and induction of cell death of MV4-11 cells were analyzed after treatment of 5a (50 μM) compound. After 72 h of treatment, a high influence on the mitochondrial membrane potential (Fig. 2) was observed, with about 45% of cells having a significantly lowered *Ψ*_m_ (which was statistically significant in comparison to the control cells). Analysis of cell death with using Annexin-V and PI staining showed a lack of induction of early apoptotic (An-V+/PI−), apoptotic (An-V+/PI+), or necrotic (An-V−/PI+) cell death (data not shown).

Compound 5a was used in a concentration about its IC_50_ in all types of analyses: cell cycle distribution, mitochondrial membrane potential, and apoptosis. Based on these results, we can observe that compound 5a was able to arrest cell cycle in the G2/M phase, leading to a decrease in the subpopulation in the S and G0/G1 phases and was able to decrease mitochondrial potential, but did not induce apoptosis. The blockage of the cells exposed to 5a at G2/M implied an inhibition of mitosis and resulted in cell proliferation inhibition. The lack of apoptosis or necrosis induction may suggest that compound 5a acted rather as a cytostatic agent. On the other hand, the decreased *Ψ*_m_ may suggest a mitochondrial autophagy.^22,23^ However, further studies are needed to explore the exact mechanisms of action of the tested compound.

## Experimental

### Substrates and chemicals for the synthesis

The enantiomerically pure form of *sn*-glycero-3-phosphocholine (GPC) was purchased from Bachem. Cinnamic acid (CA) (1a) and 3-methoxycinnamic acid (3-OMe–CA) (1b) were purchased from Sigma-Aldrich Chemical Co. The methanol, chloroform, dichloromethane and isooctane used in the reactions and for column chromatography, thin-layer chromatography, and enzymatic hydrolysis were purchased from Sigma-Aldrich as well as 4-(*N*,*N*-dimethylamino)pyridine (DMAP), *N*,*N*′-dicyclohexylcarbodiimide (DCC), triethylamine (TEA), palmitic acid, DBTO, oxalyl chloride, dioctyl sulfosuccinate sodium salt (AOT), and Dowex® 50WX8 H^+^ form (an ion-exchange resin). Phospholipase A_2_ (Lecitase 10 L; 10 000 LEU per mL) was a gift from Novozymes.

### Analysis

Column chromatography was performed on silica gel 60 with 0.1% of CA (230–400 mesh ASTM, Merck) using a solvent mixture of CHCl_3_/CH_3_OH/H_2_O (65 : 25 : 4, v/v/v). Analytical TLC was performed on Merck Kieselgel 60 F_254_ plates (0.2 mm silica gel with a fluorescent indicator UV254) with mixtures of CHCl_3_/CH_3_OH/H_2_O (65 : 25 : 4, v/v/v) as the developing system. Products were detected by spraying the plates with a solution of 10 g of Ce(SO_4_)_2_ and 20 g of phosphoromolibdenic acid in 1 L of 10% H_2_SO_4_ followed by heating or 0.05% primuline solution acetone/H_2_O (8 : 2, v/v) followed by UV (365 nm) visualization. All the NMR spectra were recorded using a Bruker Avance II 600 MHz spectrometer (Brüker, Billerica, MA, USA). High-resolution mass spectra (HRMS) were obtained using an electron spray ionization (ESI) technique on a Waters ESI-Q-TOF Premier XE spectrometer.

### Chemical synthesis

The cadmium chloride complex (GPC × CdCl_2_) was obtained according to the procedure previously described.^24,25^

#### Synthesis of symmetrically substituted phosphocholines (3a-b) containing the CA and 3-OMe–CA residues

The synthesis of the structured phospholipids (3a-b) was carried out according to the procedure described earlier.^18^ A mixture of GPC × CdCl_2_ (0.23 mmol), aromatic acid (0.92 mmol), 4-(*N*,*N*-dimethylamino)pyridine (DMAP) (0.46 mmol), and *N*,*N*′-dicyclohexylcarbodiimide (DCC) (0.97 mmol) was stirred for 72 h at 40 °C in anhydrous CH_2_Cl_2_ (12 mL), under nitrogen and protection from light. The reaction progress was monitored by TLC. When the reaction was completed, the precipitate formed during the reaction course was filtered off and Dowex® 50WX8 H^+^ form was added to the mixture. The solution was stirred for 30 min and then the resin was filtered off under reduced pressure and the solvent was evaporated *in vacuo*. The crude PC was purified by column chromatography (CHCl_3_/CH_3_OH/H_2_O (65 : 25 : 4, v/v/v)).

##### 1,2-Dicinnamoyl-*sn*-glycero-3-phosphocholine (3a)

Colourless greasy solid (93.5% yield, *R*_f_ 0.38); ^1^H NMR (600 MHz, CDCl_3_/CD_3_OD 2 : 1 (v/v)), *δ*: 2.96 (s, 9H, –N(CH_3_)_3_), 3.37 (m, 2H, CH_2_-β), 3.90 (t, *J* = 6.0 Hz, 2H, CH_2_-3′), 4.03 (m, 2H, CH_2_-α), 4.17 (dd, *J* = 12.0, 6.6 Hz, 1H, one of CH_2_-1′), 4.31 (dd, *J* = 12.0, 3.4 Hz, 1H, one of CH_2_-1′), 5.18 (m, 1H, H-2′), 6.22 (two d, *J* = 15.8 Hz, 2H, H-2_*sn*-1_, H-2_*sn*-2_), 7.11–7.13 (m, 6H, H-3′′_*sn*-1_, H-4′′_*sn*-1_, H-5′′_*sn*-1_, H-3′′_*sn*-2_, H-4′′_*sn*-2_, H-5′′_*sn*-2_), 7.28–7.29 (m, 4H, H-2′′_*sn*-1_, H-6′′_*sn*-1_, H-2′′_*sn*-2_, H-6′′_*sn*-2_), 7.45 (two d, *J* = 15.8 Hz, 2H, H-3_*sn*-1_, H-3_*sn*-2_); ^13^C NMR (150 MHz, CDCl_3_/CD_3_OD 2 : 1 (v/v)) *δ*: 53.48, 53.51, 53.53 (–N(CH_3_)_3_), 58.75 (C-α), 62.34 (C-1′), 63.38 (C-3′), 65.90 (C-β), 70.34 (C-2′), 116.56, 116.69 (C-2_*sn*-1_, C-2_*sn*-2_), 127.68, 127.69 (C-2′′_*sn*-1_, C-6′′_*sn*-1_, C-2′′_*sn*-2_, C-6′′_*sn*-2_), 128.41, 128.46 (C-3′′_*sn*-1_, C-5′′_*sn*-1_, C-3′′_*sn*-2_, C-5′′_*sn*-2_), 130.13, 130.21 (C-4′′_*sn*-1_, C-4′′_*sn*-2_), 133.56, 133.59 (C-1′′_*sn*-1_, C-1′′_*sn*-2_), 145.46, 145.64 (C-3_*sn*-1_, C-3_*sn*-2_), 166.17, 166.56 (C-1_*sn*-1_, C-1_*sn*-2_); ^31^P NMR (243 MHz, CDCl_3_/CD_3_OD 2 : 1 (v/v)) *δ*: −0.76; HRMS (ESI): *m*/*z* calcd for C_26_H_32_NO_8_P [M + H]^+^ 518.1944; found 518.1945.

##### 1,2-Di(3-methoxycinnamoyl)-*sn*-glycero-3-phosphocholine (3b)

Colourless greasy solid (94% yield, *R*_f_ 0.37); ^1^H NMR (600 MHz, CDCl_3_/CD_3_OD 2 : 1 (v/v)), *δ*: 2.99 (s, 9H, –N(CH_3_)_3_), 3.43 (t, 2H, CH_2_-β), 3.56, 3.57 (two s, 6H, 2× –OCH_3_), 3.91 (m, 2H, CH_2_-3′), 4.08 (m, 2H, CH_2_-α), 4.19 (m, 1H, one of CH_2_-1′), 4.33 (m, 1H, one of CH_2_-1′), 5.21 (m, 1H, H-2′), 6.21 (two d, *J* = 16.0 Hz, 2H, H-2_*sn*-1_, H-2_*sn*-2_), 6.69–6.71 (m, 2H, H-4′′_*sn*-1_, H-4′′_*sn*-2_), 6.78–6.85 (m, 2H, H-2′′_*sn*-1_, H-2′′_*sn*-2_), 6.86–6.89 (m, 2H, H-6′′_*sn*-1_, H-6′′_*sn*-2_), 7.02–7.06 (m, 2H, H-5′′_*sn*-1_, H-5′′_*sn*-2_), 7.41 (two d, *J* = 16.0 Hz, 2H, H-3_*sn*-1_, H-3_*sn*-2_); ^13^C NMR (150 MHz, CDCl_3_/CD_3_OD 2 : 1 (v/v)) *δ*: 53.46 (–N(CH_3_)_3_), 54.57, 54.58 (2× –OCH_3_), 58.81 (C-α), 62.45 (C-1′), 63.41 (C-3′), 65.84 (C-β), 70.44 (C-2′), 112.61, 112.72 (C-2′′_*sn*-1_, C-2′′_*sn*-2_), 115.90, (C-4′′_*sn*-1_, C-4′′_*sn*-2_), 116.80, 116.91 (C-2_*sn*-1_, C-2_*sn*-2_), 120.35 (C-6′′_*sn*-1_, C-6′′_*sn*-2_), 129.40, 129.47 (C-5′′_*sn*-1_, C-5′′_*sn*-2_), 134.84, 134.89 (C-1′′_*sn*-1_, C-1′′_*sn*-2_), 145.39, 145.64 (C-3_*sn*-1_, C-3_*sn*-2_), 159.46, 159.50 (C-3′′_*sn*-1_, C-3′′_*sn*-2_), 166.17, 166.52 (C-1_*sn*-1_, C-1_*sn*-2_); ^31^P NMR (243 MHz, CDCl_3_/CD_3_OD 2 : 1 (v/v)) *δ*: −3.44; HRMS (ESI): *m*/*z* calcd for C_28_H_36_NO_10_P [M + H]^+^ 578.2155; found 578.2156.

#### Synthesis of asymmetrically substituted phosphocholines (5a-b) containing the CA and 3-OMe–CA residues in the *sn*-2 position

Aromatic acid (0.604 mmol) was added to a CH_2_Cl_2_ solution (8 mL) containing 1-palmitoyl-2-hydroxy-*sn*-3-glycero-phosphocholine (0.302 mmol) and DMAP (0.6 mmol). Next the DCC (1.3 mmol) dissolved in CH_2_Cl_2_ (4 mL) was added to the mixture. The reaction was carried out for 72 h in a nitrogen atmosphere in the dark at 40 °C. The product was extracted and purified according to the procedure described for compounds 3a and 3b.

##### 1-Palmitoyl-2-cinnamoyl-*sn*-glycero-3-phosphocholine (5a)

Colourless greasy solid (90% yield, *R*_f_ 0.31); ^1^H NMR (600 MHz, CDCl_3_/CD_3_OD 2 : 1 (v/v)), *δ*: 0.64 (t, *J* = 6.8 Hz, 3H, CH_3_(CH_2_)_13_CH_2_C(O)), 0.97–1.05 (m, 24H, CH_3_(CH_2_)_12_CH_2_CH_2_C(O)), 1.35 (m, 2H, CH_3_(CH_2_)_12_CH_2_CH_2_C(O)), 2.09 (t, *J* = 7.4 Hz, 2H, CH_3_(CH_2_)_13_CH_2_C(O)), 2.97 (s, 9H, –N(CH_3_)_3_), 3.37 (m, 2H, CH_2_-β), 3.85 (m, 2H, CH_2_-3′), 4.02–4.07 (m, 3H, CH_2_-α, one of CH_2_-1), 4.13 (m, 1H, one of CH_2_-1′), 5.12 (m, 1H, H-2′), 6.23 (d, *J* = 16.0 Hz, 1H, H-2), 7.17 (m, 3H, H-3′′, H-4′′, H-5′′), 7.31 (m, 2H, H-2′′, H-6′′), 7.47 (d, *J* = 16.0 Hz, 1H, H-3); ^13^C NMR (150 MHz, CDCl_3_/CD_3_OD 2 : 1 (v/v)) *δ*: 13.40 (CH_3_(CH_2_)_13_CH_2_C(O)), 22.17 (CH_3_CH_2_(CH_2_)_12_CH_2_C(O)), 24.42 (CH_3_(CH_2_)_12_CH_2_CH_2_C(O)), 28.63, 28.74, 28.81, 28.86, 28.99, 29.01, 29.13, 29.15, 29.17, 29.19, 31.44 (CH_3_CH_2_(CH_2_)_11_CH_2_CH_2_C(O)), 33.62 (CH_3_(CH_2_)_13_CH_2_C(O)), 53.56, 53.59, 53.61 (–N(CH_3_)_3_), 58.75 (C-α), 62.16 (C-1′), 63.51 (C-3′), 65.96 (C-β), 70.34 (C-2′), 116.74 (C-2), 127.73 (C-2′′, C-6′′), 128.50, 128.53 (C-3′′, C-5′′), 130.28 (C-4′′), 133.62 (C-1′′), 145.60 (C-3), 166.13 (C-1), 173.63 (CH_3_(CH_2_)_13_CH_2_C(O)); ^31^P NMR (243 MHz, CDCl_3_/CD_3_OD 2 : 1 (v/v)) *δ*: −0.66; HRMS (ESI): *m*/*z* calcd for C_33_H_56_NO_8_P [M + H]^+^ 626.3822; found 626.3824.

##### 1-Palmitoyl-2-3-methoxycinnamoyl-*sn*-glycero-3-phosphocholine (5b)

Colourless greasy solid (58% yield, *R*_f_ 0.33); ^1^H NMR (600 MHz, CDCl_3_/CD_3_OD 2 : 1 (v/v)), *δ*: 0.62 (t, *J* = 6.8 Hz, 3H, CH_3_(CH_2_)_13_CH_2_C(O)), 0.95–1.09 (m, 24H, CH_3_(CH_2_)_12_CH_2_CH_2_C(O)), 1.33 (m, 2H, CH_3_(CH_2_)_12_CH_2_CH_2_C(O)), 2.07 (t, *J* = 7.5 Hz, 2H, CH_3_(CH_2_)_13_CH_2_C(O)), 2.96 (s, 9H, –N(CH_3_)_3_), 3.39 (m, 2H, CH_2_-β), 3.58 (s, 3H, –OCH_3_), 3.81 (m, 2H, CH_2_-3′), 4.01–4.04 (m, 3H, CH_2_-α, one of CH_2_-1′), 4.17 (m, 2H, one of CH_2_-1′), 5.12 (m, 1H, H-2′), 6.20 (d, *J* = 15.9 Hz, 1H, H-2), 6.72 (m, 1H, H-4′′), 6.83 (m, 1H, H-2′′), 6.90 (m, 1H, H-6′′), 7.07 (m, 1H, H-5′′), 7.42 (d, *J* = 15.9 Hz, 1H, H-3); ^13^C NMR (150 MHz, CDCl_3_/CD_3_OD 2 : 1 (v/v)) *δ*: 13.35 (CH_3_(CH_2_)_13_CH_2_C(O)), 22.13 (CH_3_CH_2_(CH_2_)_12_CH_2_C(O)), 24.38 (CH_3_(CH_2_)_12_CH_2_CH_2_C(O)), 28.61, 28.80, 28.82, 28.96, 29.11, 29.14, 31.40 (CH_3_CH_2_(CH_2_)_11_CH_2_CH_2_C(O)), 33.58 (CH_3_(CH_2_)_13_CH_2_C(O)), 53.49 (–N(CH_3_)_3_), 54.69 (–OCH_3_), 58.86 (C-α), 62.24 (C-1′), 63.47 (C-3′), 65.83 (C-β), 70.44 (C-2′), 112.80 (C-2′′), 116.00 (C-4′′), 116.88 (C-2), 120.40 (C-6′′), 129.55 (C-5′′), 134.88 (C-1′′), 145.72 (C-3), 159.56 (C-3′′), 166.26 (C-1), 173.75 (CH_3_(CH_2_)_13_CH_2_C(O)); ^31^P NMR (243 MHz, CDCl_3_/CD_3_OD 2 : 1 (v/v)) *δ*: −4.64; HRMS (ESI): *m*/*z* calcd for C_34_H_58_NO_9_P [M + H]^+^ 656.3928; found 656.3925.

#### Synthesis of 2-lyso-phosphatidylcholines (9a-b) containing the CA and 3-OMe–CA residues in the *sn*-1 position

GPC (1 mmol) and DBTO (1 mmol) were suspended in 12 mL of anhydrous propan-2-ol and refluxed for 1 h. The reaction mixture was cooled to room temperature and TEA (2.4 mmol) was added, followed by the chloride of CA and 3-OMe–CA acids (2.4 mmol) in 5 mL of anhydrous propan-2-ol. After 1 h of reaction, the mixture was filtered using Celite® 545 and washed CH_2_Cl_2_. In the next step, the solvent was evaporated under vacuum and the crude product was purified on a silica gel column (CHCl_3_/CH_3_OH/H_2_O (65 : 25 : 4, v/v/v)).

##### 1-Cinnamoyl-2-hydroxy-*sn*-glycero-3-phosphocholine (9a)

Colourless greasy solid (67% yield, *R*_f_ 0.17); ^1^H NMR (600 MHz, CDCl_3_/CD_3_OD 2 : 1 (v/v)), *δ*: 3.01 (s, 9H, –N(CH_3_)_3_), 3.44 (m, 2H, CH_2_-β), 3.73–3.79 (two m, 3H, H-2′, CH_2_-3′), 3.85 (m, 1H, –OH), 4.00–4.10 (two m, 4H, CH_2_-α, CH_2_-1′), 6.25 (d, *J* = 16.0 Hz, 1H, H-2), 7.16 (m, 3H, H-3′′, H-4′′, H-5′′), 7.31 (m, 2H, H-2′′, H-6′′), 7.48 (d, *J* = 16.0 Hz, 1H, H-3); ^13^C NMR (151 MHz, CDCl_3_/CD_3_OD 2 : 1 (v/v)) *δ*: 53.57, 53.60, 53.62 (–N(CH_3_)_3_), 58.97 (C-α), 64.72 (C-1′), 65.88 (C-β), 66.60 (C-3′), 68.17 (C-2′), 116.85 (C-2), 127.68, (C-2′′, C-6′′), 128.50, 128.53 (C-3′′, C-5′′), 130.16 (C-4′′), 133.72 (C-1′′), 145.27 (C-3), 16.91 (C-1); ^31^P NMR (243 MHz, CDCl_3_/CD_3_OD 2 : 1 (v/v)) *δ*: −1.26; HRMS (ESI): *m*/*z* calcd for C_17_H_26_NO_7_P [M + H]^+^ 388.1525; found 388.1526.

##### 1-(3-Methoxycinnamoyl)-2-hydroxy-*sn*-glycero-3-phosphocholine (9b)

Colourless greasy solid (69% yield, *R*_f_ 0.15); ^1^H NMR (600 MHz, CDCl_3_/CD_3_OD 2 : 1 (v/v)), *δ*: 3.01 (s, 9H, –N(CH_3_)_3_), 3.49 (m, 2H, CH_2_-β), 3.58 (s, 3H, –OCH_3_), 3.77–3.85 (m, 3H, H-2′, CH_2_-3′, –OH), 4.00–4.12 (m, 4H, CH_2_-1′, CH_2_-α), 6.23 (d, *J* = 16 Hz, 1H, H-2), 6.71 (m, 1H, H-4′′), 6.83 (m, 1H, H-2′′), 6.90 (m, 1H, H-6′′), 7.06 (m, 1H, H-5′′), 7.44 (d, *J* = 16 Hz, 1H, H-3); ^13^C NMR (151 MHz, CDCl_3_/CD_3_OD 2 : 1 (v/v)) *δ*: 53.61 (–N(CH_3_)_3_), 54.71 (–OCH_3_), 59.21 (C-α), 64.73 (C-1′), 65.78 (C-β), 66.53 (C-3′), 68.04 (C-2′), 112.76 (C-2′′), 115.84 (C-4′′), 117.04 (C-2), 120.35 (C-6′′), 129.52 (C-5′′), 135.01 (C-1′′), 145.29 (C-3), 159.51 (C-3′′), 166.94 (C-1); ^31^P NMR (243 MHz, CDCl_3_/CD_3_OD 2 : 1 (v/v)) *δ*: −1.26; HRMS (ESI): *m*/*z* calcd for C_18_H_28_NO_8_P [M + H]^+^ 418.1631; found 418.1640.

#### Synthesis of asymmetrically substituted phosphocholines (10a-b) containing the CA and 3-OMe–CA residues in the *sn*-1 position

The synthesis of 10a-b was carried in the same scale and according to the procedure described for 5a and 5b.

##### 1-Cinnamoyl-2-palmitoyl-*sn*-glycero-3-phosphocholine (10a)

Colourless greasy solid (22% yield, *R*_f_ 0.29); ^1^H NMR (600 MHz, CDCl_3_/CD_3_OD 2 : 1 (v/v)), *δ*: 0.66 (t, *J* = 7.0 Hz, 3H, CH_3_(CH_2_)_13_CH_2_C(O)), 0.99–1.08 (m, 24H, CH_3_(CH_2_)_12_CH_2_CH_2_C(O)), 1.39 (m, 2H, CH_3_(CH_2_)_12_CH_2_CH_2_C(O)), 2.13–2.15 (m, 2H, CH_3_(CH_2_)_13_CH_2_C(O)), 3.01 (s, 9H, –N(CH_3_)_3_), 3.43 (m, 2H, CH_2_-β), 3.82 (t, *J* = 6.5 Hz, 2H, CH_2_-3′), 4.06 (m, 2H, CH_2_-α), 4.12 (dd, *J* = 12.0, 7.0 Hz, 1H, one of CH_2_-1′), 4.27 (dd, *J* = 12.0, 3.0 Hz, 1H, one of CH_2_-1′), 5.10 (m, 1H, H-2′), 6.25 (m, 1H, H-2), 7.19–7.20 (m, 3H, H-3′′, H-4′′, H-5′′), 7.33–7.36 (m, 2H, H-2′′, H-6′′), 7.48 (m, 1H, H-3); ^13^C NMR (150 MHz, CDCl_3_/CD_3_OD 2 : 1 (v/v)) *δ*: 13.45 (CH_3_(CH_2_)_13_CH_2_C(O)), 22.20 (CH_3_CH_2_(CH_2_)_12_CH_2_C(O)), 24.51 (CH_3_(CH_2_)_12_CH_2_CH_2_C(O)), 28.66, 28.70, 28.86, 28.89, 28.90, 29.02, 29.06, 29.18, 29.21, 31.46 (CH_3_CH_2_(CH_2_)_11_CH_2_CH_2_C(O)), 33.81 (CH_3_(CH_2_)_13_CH_2_C(O)), 53.58 (–N(CH_3_)_3_), 58.91 (C-α), 62.49 (C-1′), 63.36 (C-3′), 65.88 (C-β), 70.05 (C-2′), 116.51 (C-2), 127.78 (C-2′′, C-6′′), 128.54 (C-3′′, C-5′′), 130.32 (C-4′′), 133.60 (C-1′′), 145.67 (C-3), 166.71 (C-1), 173.54 (CH_3_(CH_2_)_13_CH_2_C(O)); ^31^P NMR (243 MHz, CDCl_3_/CD_3_OD 2 : 1 (v/v)) *δ*: −4.2; HRMS (ESI): *m*/*z* calcd for C_33_H_56_NO_8_P [M + H]^+^ 626.3822; found 626.3824.

##### 1-3-Methoxycinnamoyl-2-palmitoyl-*sn*-glycero-3-phosphocholine (10b)

Colourless greasy solid (14% yield, *R*_f_ 0.29); ^1^H NMR (600 MHz, CDCl_3_/CD_3_OD 2 : 1 (v/v)), *δ*: 0.63 (t, *J* = 7 Hz, 3H, CH_3_(CH_2_)_13_CH_2_C(O)), 0.96–1.01 (m, 24H, CH_3_(CH_2_)_12_CH_2_CH_2_C(O)), 1.35–1.38 (m, 2H, CH_3_(CH_2_)_12_CH_2_CH_2_C(O)), 2.09–2.12 (m, 2H, CH_3_(CH_2_)_13_CH_2_C(O)), 2.98 (s, 9H, –N(CH_3_)_3_), 3.39 (m, 2H, CH_2_-β), 3.60 (s, 3H, –OCH_3_), 3.79 (t, *J* = 6,5 Hz, 2H, CH_2_-3′), 4.03 (m, 2H, CH_2_-α), 4.08–4.11 (m, 2H, CH_2_-1′), 5.07 (m, 1H, H-2′), 6.18–6.21 (m, 1H, H-2), 6.72–6.73 (m, 1H, H-4′′), 6.83 (m, 1H, H-2′′), 6.89–6.90 (m, 1H, H-6′′), 7.08 (m, 1H, H-5′′), 7.39–7.42 (m, 1H, H-3); ^13^C NMR (150 MHz, CDCl_3_/CD_3_OD 2 : 1 (v/v)) *δ*: 13.38 (CH_3_(CH_2_)_13_CH_2_C(O)), 22.15 (CH_3_CH_2_(CH_2_)_12_CH_2_C(O)), 24.47 (CH_3_(CH_2_)_12_CH_2_CH_2_C(O)), 28.61, 28.81, 28.84, 28.98, 29.01, 29.14, 29.16, 31.42 (CH_3_CH_2_(CH_2_)_11_CH_2_CH_2_C(O)), 33.76 (CH_3_(CH_2_)_13_CH_2_C(O)), 53.50, 53.53, 53.55 (–N(CH_3_)_3_), 54.73 (OCH_3_), 58.85 (C-α), 62.46 (C-1′), 63.31 (C-3′), 65.87 (C-β), 70.03 (C-2′), 112.76 (C-2′′), 116.80 (C-4′′), 120.43 (C-6′′), 129.54 (C-5′′), 134.94 (C-1′′), 145.51 (C-3), 166.61 (C-1), 173.48 (CH_3_(CH_2_)_13_CH_2_C(O)); ^31^P NMR (243 MHz, CDCl_3_/CD_3_OD 2 : 1 (v/v)) *δ*: −3.18; HRMS (ESI): *m*/*z* calcd for C_34_H_58_NO_9_P [M + H]^+^ 656.3928; found 656.3918.

### Biological studies

#### Cell lines

Biophenotypic B myelomonocytic leukaemia MV4-11, human lung carcinoma A549, human breast carcinoma MCF-7, human colon carcinoma LoVo, its drug resistant line LoVo/DX, liver carcinoma HepG2, and normal mouse fibroblast BALB/3T3 cells were obtained from the American Type Culture Collection (Rockville, Maryland, USA). All lines are being maintained at the Institute of Immunology and Experimental Therapy, Wrocław, Poland. All cell lines were grown in a humid atmosphere at 37 °C with 5% CO_2_ and were cultured in media according to the method described before.^18^

#### Antiproliferative assay *in vitro*

Prior to usage, the compounds were dissolved in DMSO to the concentration of 25 or 50 mM, and subsequently diluted in the culture medium to reach the required concentrations (ranging from 5 to 625 μM).

Twenty-four hours before addition of the tested compounds, the cells were plated in 96-well plates (Sarstedt, USA) at a density of 10^4^ cells per well. All cell lines were exposed to each tested phosphatidylcholines for 72 h. Cells were also exposed to the commercially available drugs cisplatin and doxorubicin. Cell lines were also exposed to the solvent used for the tested compounds (DMSO) at concentrations corresponding to those present in the tested agents' dilutions. For adherent cells, a SRB assay was performed and an MTT assay was performed for leukaemia cells.

#### SRB assays

The cytotoxicity assay was performed after 72 h exposure of the cultured cells to varying concentrations (from 5 to 625 μM) of the tested agents. The cells attached to the plastic were fixed *in situ* by gently adding 50 μL per well of cold 50% TCA (trichloroacetic acid). The plates were incubated at 4 °C for 1 h and then washed five times with tap water. The background optical density was measured in the wells filled with culture medium, without the cells. The cellular material fixed with TCA was stained with 50 μL of 0.4% sulforhodamine B (SRB) dissolved in 1% acetic acid for 30 min. Unbound dye was removed by rinsing (4×) with 1% acetic acid. The protein-bound dye was extracted with 10 mM unbuffered Tris base for determination of the optical density (at 540 nm) in a computer-interfaced 96-well microtiter plate reader Multiskan RC photometer (Labsystems, Helsinki, Finland). Each compound in the given concentration was tested in triplicate in each experiment, which was repeated 3–5 times.

#### MTT assays

This technique was applied for the cytotoxicity screening against leukemia cells. An assay was performed after 72 h exposure to varying concentrations (from 5 to 625 μM) of the tested agents. For the last 3–4 hours of incubation. 20 μL of MTT solution was added to each well (MTT: 3-(4,5-dimethylthiazol-2-yl)-2,5-diphenyl tetrazolium bromide; stock solution: 5 mg mL^−1^, Sigma, Germany). The mitochondria of viable cells reduce the pale yellow MTT to a navy blue formazan: the more viable cells are present in a well, the more MTT will be reduced to formazan. When the incubation time was completed, 80 μL of the lysing mixture was added to each well (lysing mixture: 225 mL dimethylformamide, 67.5 g sodium dodecyl sulphate, and 275 mL of distilled water). After 24 h, when formazan crystals had been dissolved, the optical densities of the samples were read with a Multiskan RC photometer at 570 nm wavelength. Each compound in a given concentration was tested in triplicate in each experiment, which was repeated 3–5 times.

The results of the cytotoxic activity *in vitro* were expressed as IC_50_—the concentration of compound (in μM) that inhibits the proliferation rate of the tumor cells by 50% as compared to control untreated cells.

#### Cell cycle analysis

For cell cycle analysis, the cells MV4-11 were treated with the tested compound 5a dissolved in DMSO at a concentration of 50 μM. The cells were treated also only by the DMSO for comparison. After 72 h of incubation, the cells (1 × 10^6^) were trypsinized and rinsed with cold PBS. Washed cell pellets were fixed for 24 h in 70% ethanol at −20 °C. After fixation, the cells were washed twice with PBS once again and resuspended in PBS. Next, RNAse (8 μg mL^−1^, Fermentas, Germany) was added and the cells were incubated for 1 h at 37 °C. The cells were then stained with propidium iodide (50 μg mL^−1^, Sigma Aldrich, Germany) at 4 °C for 30 min and the cellular DNA content was analyzed by flow cytometry using a BD LSRFortessa cytometer (BD Bioscience, San Jose, USA). Compounds at each concentration were tested at least three times independently. The control in the assay was the cells exposed to DMSO at a concentration of 0.05%. Data were analyzed using ModFit 3.2 software (Verity Software, USA).

#### Determination of the mitochondrial membrane potential

The loss of mitochondrial membrane potential was estimated using the fluorescent probe JC-1 (Sigma Aldrich, Germany). This dye binds to nucleic acids and emits red fluorescence, with a spectral overlap with the orange fluorescence of JC-1 aggregates. The cells were exposed to the tested compound 5a at a concentration of 50 μM. The MV4-11 cells (5 × 10^5^) were washed in phosphate-buffered saline (PBS) containing 2% FBS. The pelleted cells were resuspended in 100 μL of the warm cultured medium with the addition of 10 μL of JC-1 (the final concentration of JC-1 was 3 μg mL^−1^) and were then incubated for 30 min at 37 °C. Next, the cells were washed with 1 mL of PBS with 2% FBS and resuspended in 300 μL of PBS with 2% FBS. The mitochondrial membrane potential was analyzed by flow cytometry using BD LSRFortessa cytometry. Compounds at each concentration were tested at least three times independently. The control in the assay was the cells exposed to DMSO at a concentrations of 0.05%. Data were analyzed using Flowing software 2.5.1.

#### Cell death determination by Annexin V and PI staining

The MV4-11 cells were exposed to the test compound 5a at a concentration of 50 μM for 72 h. After incubation, the cells (2 × 10^5^) were washed twice with PBS. APC-Annexin V (BD Pharmingen) was dissolved to the concentration of 1 mg mL^−1^ in a binding buffer (HEPES buffer: 10 mM HEPES/NaOH), pH 7.4, 150 mM NaCl, 5 mM KCl, 1 mM MgCl_2_, 1.8 mM CaCl_2_, (IIET, Poland) and the cells were suspended in 200 μL of this 1 mg mL^−1^ solution (freshly prepared each time). After the incubation in the dark (15 min) at room temperature, the solution (1 mg mL^−1^) of propidium iodide (PI) was added prior to the analysis to give a final concentration of 0.1 mg mL^−1^. Data acquisition was performed by flow cytometry using a BD LSRFortessa cytometer. Compound 5a was tested at least three times independently. The control in the assay was the cells exposed to DMSO at a concentration of 0.05%. Results were analyzed using Flowing software 2.5.1. The data were displayed as a two-color dot plot with APC-Annexin V *vs.* PI. Double-negative cells were live cells, PI+/Annexin V+ were late apoptotic or necrotic cells, and PI-/Annexin V+ were early apoptotic.

#### Statistical analysis

Statistical analysis was performed in Statsoft Statistica 10. All datasets were analyzed using *t*-test. *p*-Values lower than 0.05 were considered as statistically significant.

## Conclusions

In summary, we successfully synthesized eight new phosphatidylcholines as potential chemotherapeutics in the treatment of cancers. We evaluated the antiproliferative activities of these conjugates against six human cancer cell lines. We identified that some phosphatidylcholines posses high *in vitro* anticancer activity and at estimated doses are not toxic toward normal BALB/3T3 cells. The highest antiproliferative activity was observed for 1-palmitoyl-2-cinnamoyl-*sn*-glycero-3-phosphocholine (5a). Mechanistic studies showed that 5a caused cell cycle arrest in the G2/M phase, decreasing the mitochondrial membrane potential, but with no induction of apoptosis induction. Our studies suggest that the production of phospholipid derivatives containing aromatic acid residues is a convenient method to obtain more active products that can play a role as new antitumor therapeutics.

## Conflicts of interest

There are no conflicts to declare.

## Supplementary Material

RA-008-C8RA07002D-s001
