# Supplementary material for: Synthesis and biological evaluation of phosphatidylcholines with cinnamic and 3-methoxycinnamic acids with potent antiproliferative activity
Source: RSC Adv. 2018 Oct 19;8(62):35744–52. doi: 10.1039/c8ra07002d (PMC9088016; doi:10.1039/c8ra07002d)

Electronic Supplementary Material (ESI) for RSC Advances.
This journal is © The Royal Society of Chemistry 2018

Supplementary Materials

Synthesis and biological evaluation of phosphatidylcholines with cinnamic and 3-methoxycinnamic acids with potent antiproliferative activity

Marta Czarnecka *[a], Marta Świtalska [b], Joanna Wietrzyk [b], Gabriela Maciejewska [c] and Anna Gliszczyńska *[a]

[a] Department of Chemistry, Wrocław University of Environmental and Life Sciences, Norwida 25, 50-375 Wrocław, Poland
[b] Ludwik Hirszfeld Institute of Immunology and Experimental Therapy, Polish Academy of Sciences, Department of Experimental Oncology, Weigla 12, 53-114 Wrocław, Poland
[c] Central Laboratory of the Instrumental Analysis, Wrocław University of Technology, Wybrzeże Wyspiańskiego 27, 50-370 Wrocław, Poland

* Correspondence: anna.gliszczynska@wp.pl, marta.b.czarnecka@gmail.com; Tel.: +48-71-320-5183

Content

Figure S1: ^{1}H NMR spectrum of 3a. 4
Figure S2: ^{13}C NMR spectrum of 3a. 5
Figure S3: ^{31}P NMR spectrum of 3a. 6
Figure S4: ^{1}H – ^{1}H COSY spectrum of 3a. 7
Figure S5: HSQC spectrum of 3a. 8
Figure S6: ^{1}H NMR spectrum of 3b. 10
Figure S7: ^{13}C NMR spectrum of 3b. 11
Figure S8: ^{31}P NMR spectrum of 3b. 12
Figure S9: ^{1}H – ^{1}H COSY spectrum of 3b. 13
Figure S10: HSQC spectrum of 3b. 14
Figure S11: ^{1}H NMR spectrum of 5a. 16
Figure S12: ^{13}C NMR spectrum of 5a. 17
Figure S13: ^{31}P NMR spectrum of 5a. 18
Figure S14: ^{1}H – ^{1}H COSY spectrum of 5a. 19
Figure S15: HSQC spectrum of 5a. 20
Figure S16: ^{1}H NMR spectrum of 5b. 22
Figure S17: ^{13}C NMR spectrum of 5b. 23
Figure S18: ^{31}P NMR spectrum of 5b. 24
Figure S19: ^{1}H – ^{1}H COSY spectrum of 5b. 25

Figure S20: HSQC spectrum of 5b. 26
Figure S21: ^{1}H NMR spectrum of 9a. 28
Figure S22: ^{13}C NMR spectrum of 9a. 29
Figure S23: ^{31}P NMR spectrum of 9a. 30
Figure S24: ^{1}H – ^{1}H COSY spectrum of 9a. 31
Figure S25: HSQC spectrum of 9a. 32
Figure S26: ^{1}H NMR spectrum of 9b. 34
Figure S27: ^{13}C NMR spectrum of 9b. 35
Figure S28: ^{31}P NMR spectrum of 9b. 36
Figure S29: ^{1}H – ^{1}H COSY spectrum of 9b. 37
Figure S30: HSQC spectrum of 9b. 38
Figure S31: ^{1}H NMR spectrum of 10a. 40
Figure S32: ^{13}C NMR spectrum of 10a. 41
Figure S33: ^{31}P NMR spectrum of 10a. 42
Figure S34: ^{1}H – ^{1}H COSY spectrum of 10a. 43
Figure S35: HSQC spectrum of 10a. 44
Figure S36: ^{1}H NMR spectrum of 10b. 46
Figure S37: ^{13}C NMR spectrum of 10b. 47
Figure S38: ^{31}P NMR spectrum of 10b. 48
Figure S39: ^{1}H – ^{1}H COSY spectrum of 10b. 49
Figure S40: HSQC spectrum of 10b. 50

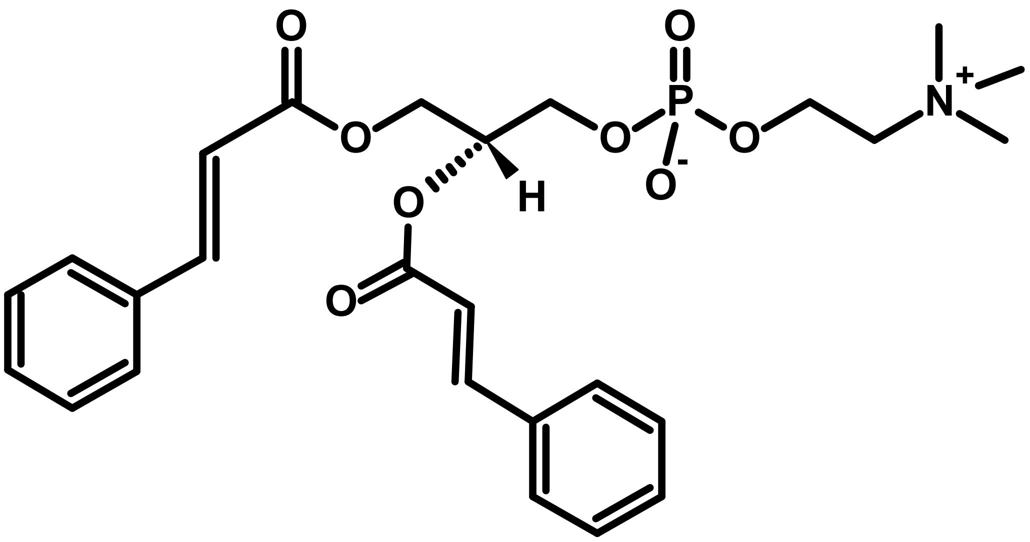

1,2-dicinnamoyl-*sn*-glycero-3-phosphocholine (**3a**)

Figure S1: ^{1}H NMR spectrum of 3a.

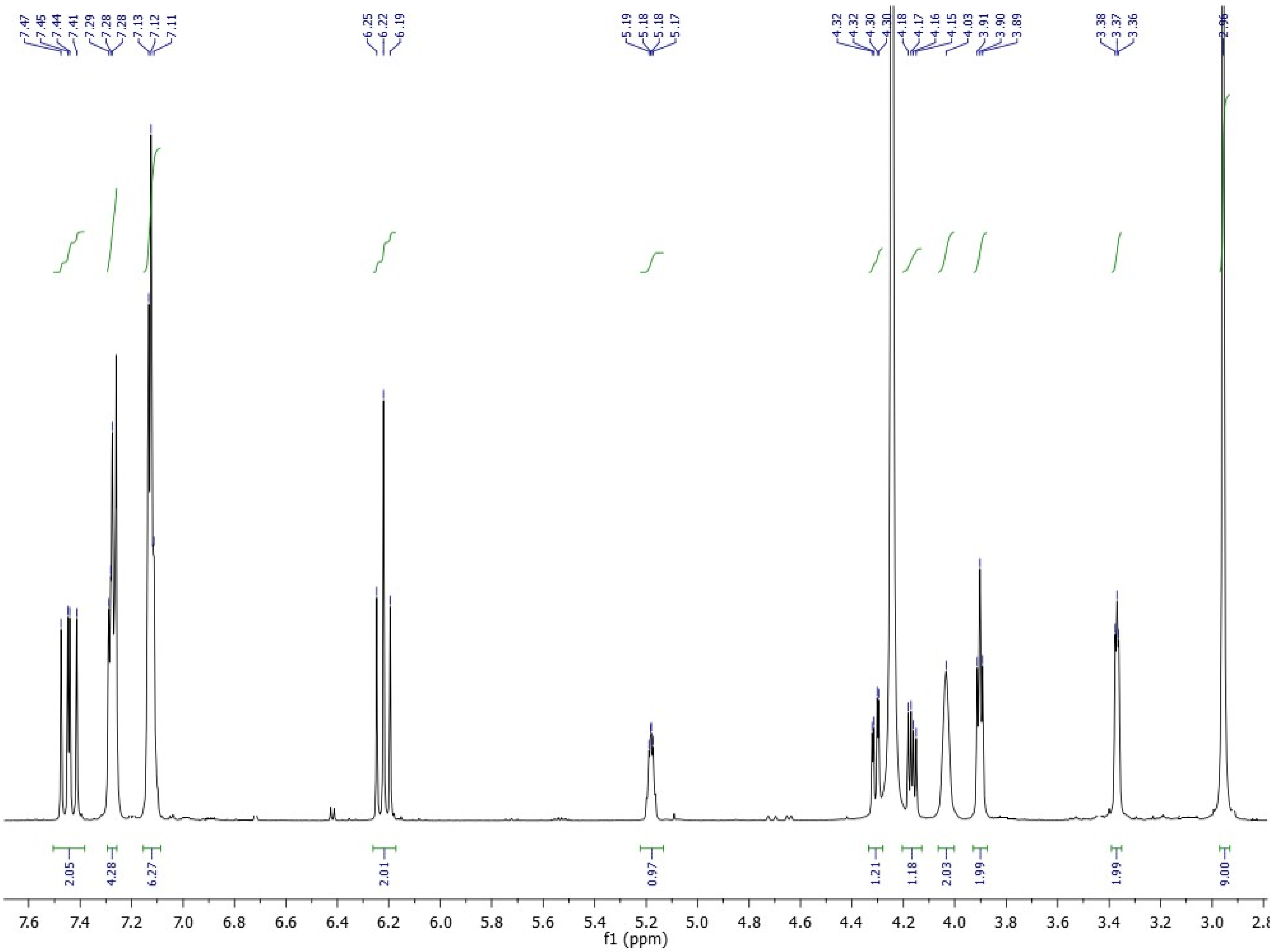

Figure S2: ^{13}C NMR spectrum of 3a.

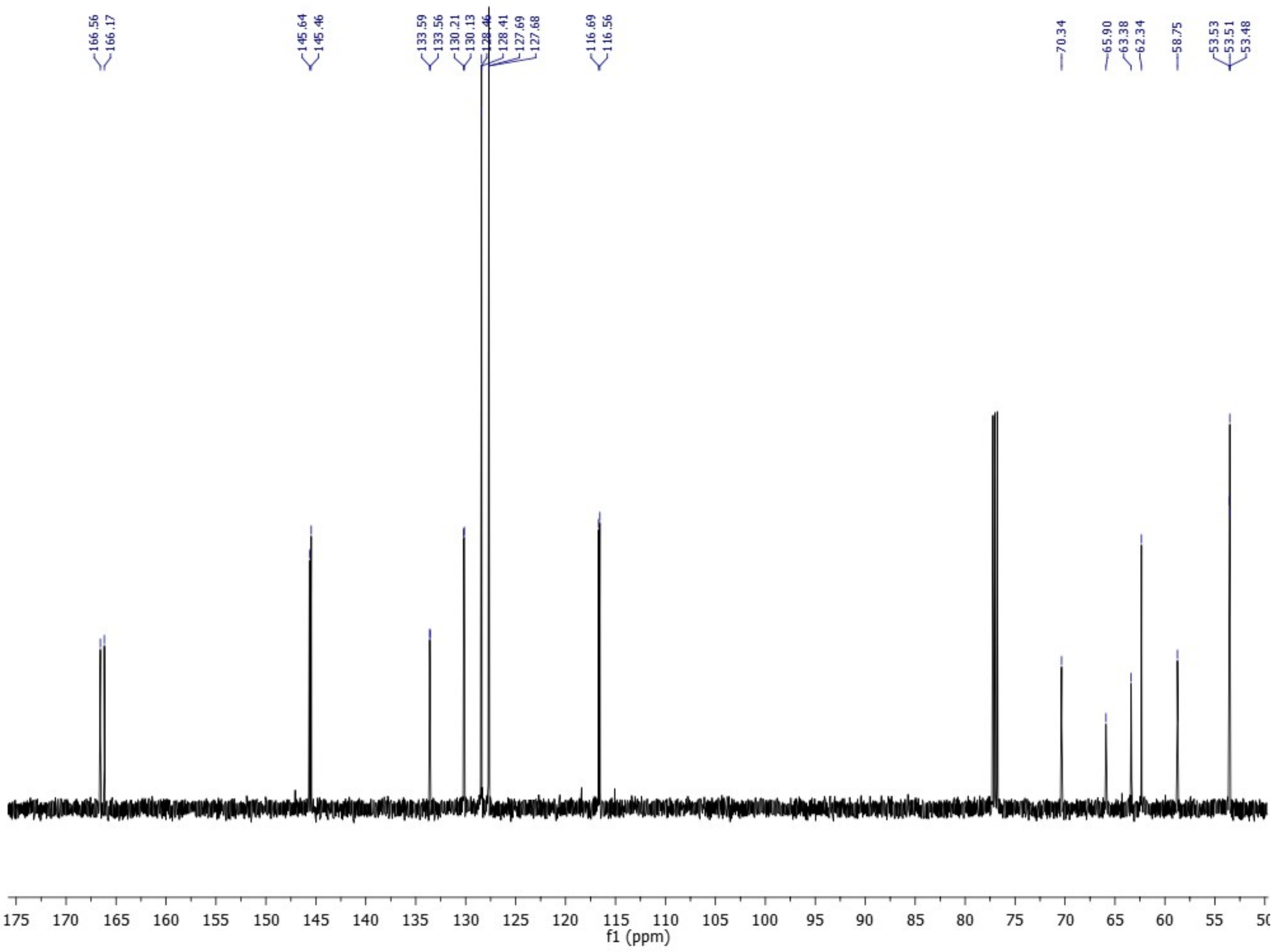

Figure S3: ^{31}P NMR spectrum of 3a.

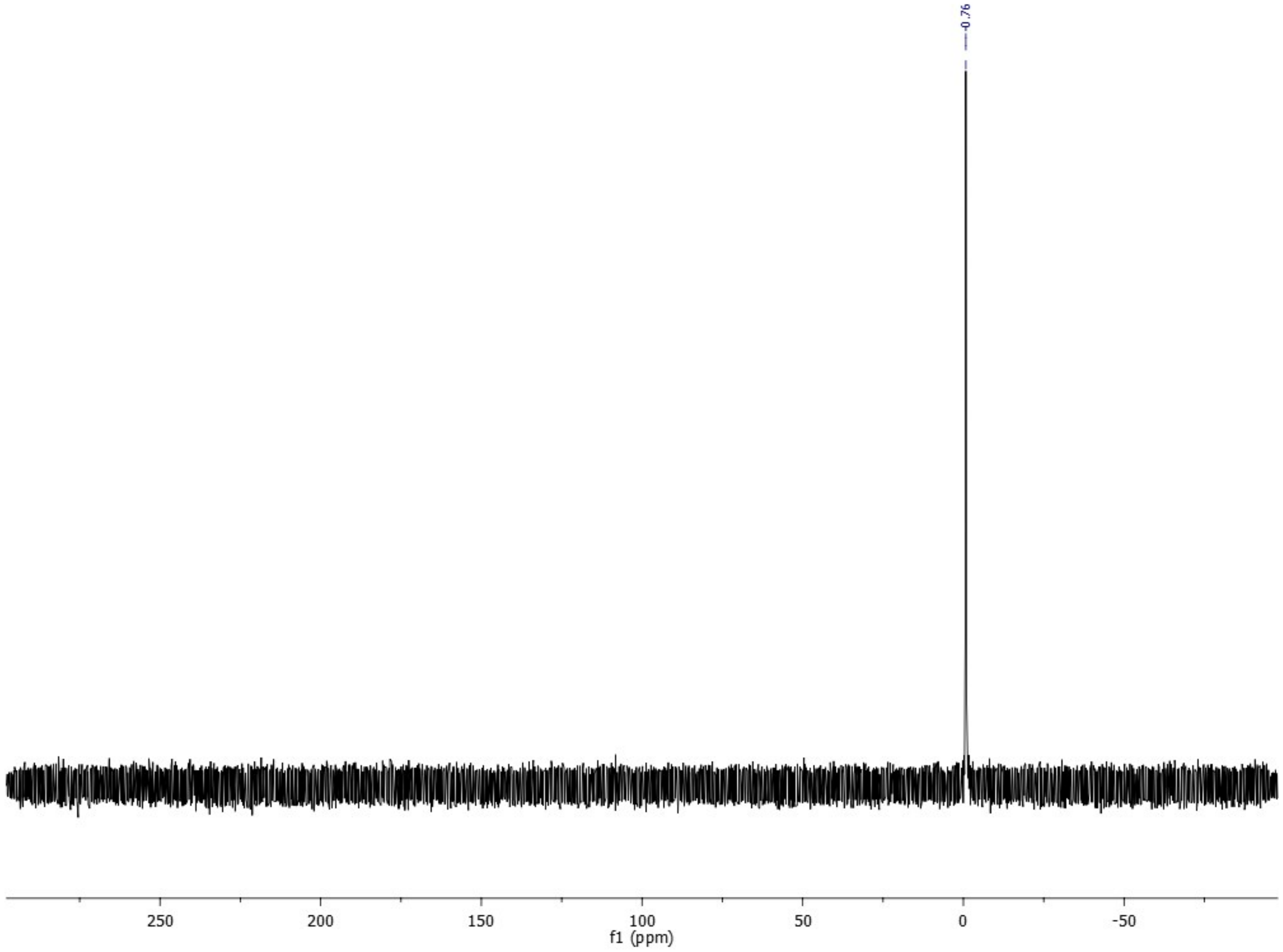

Figure S4: ^{1}H – ^{1}H COSY spectrum of 3a.

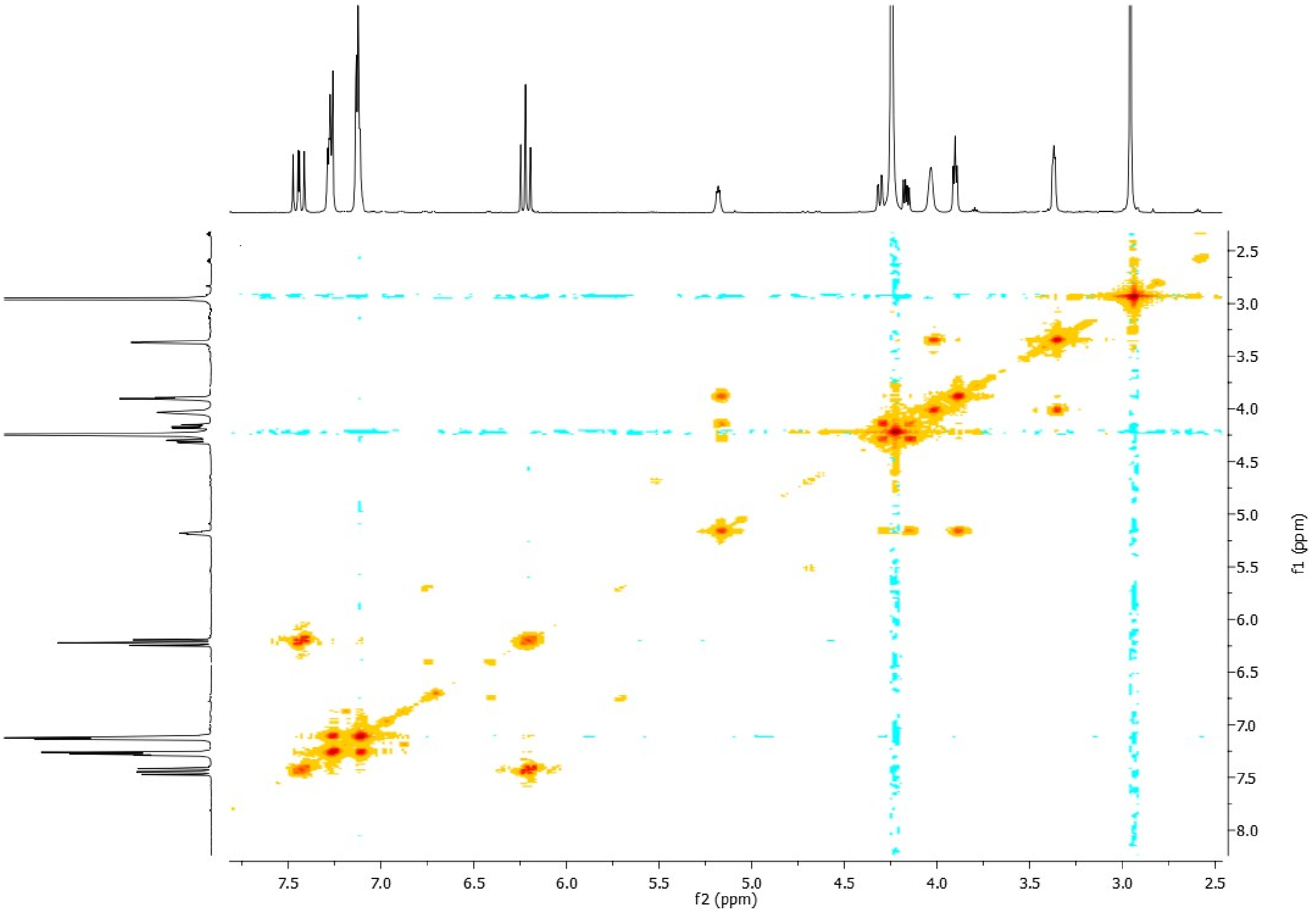

Figure S5: HSQC spectrum of 3a.

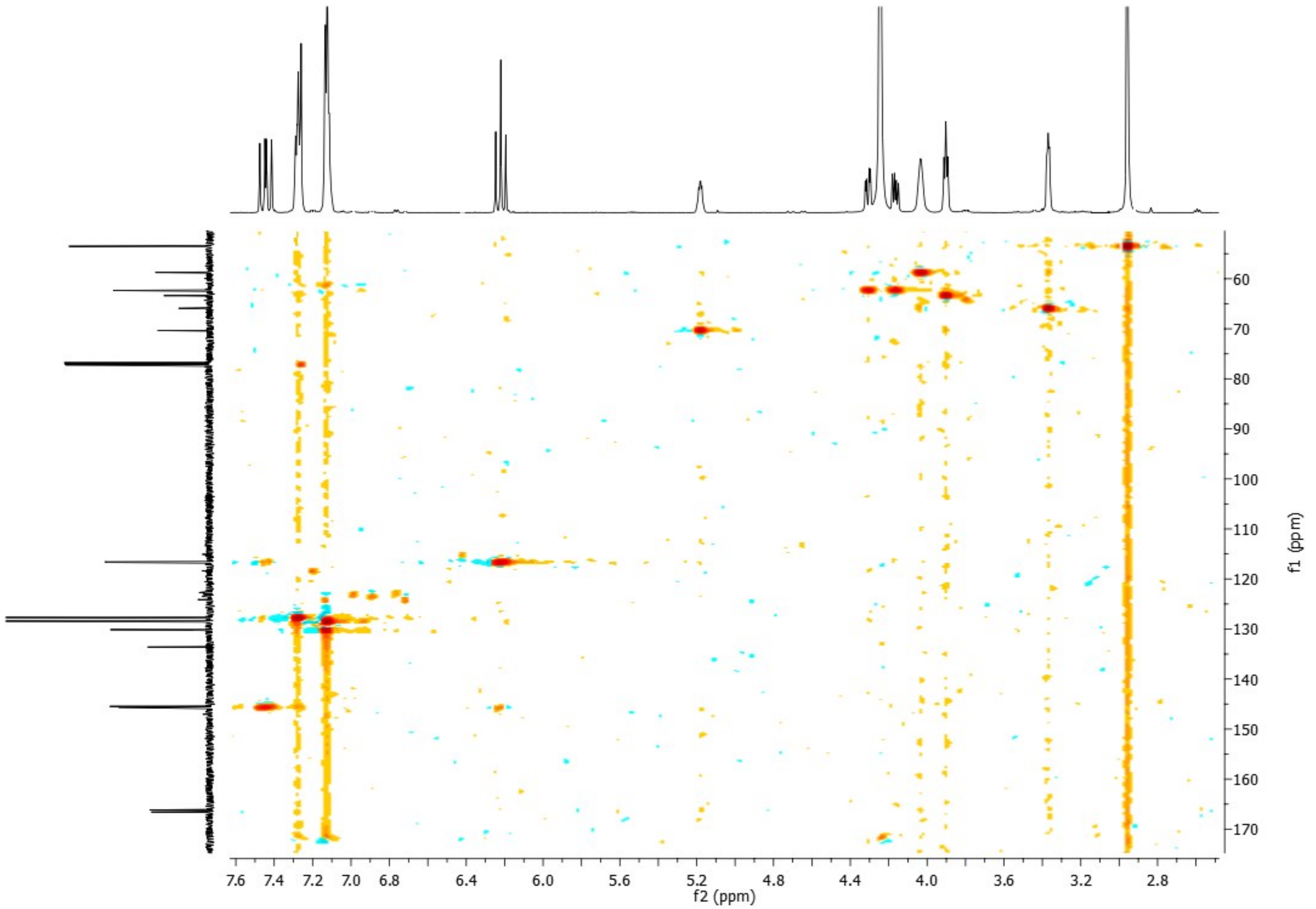

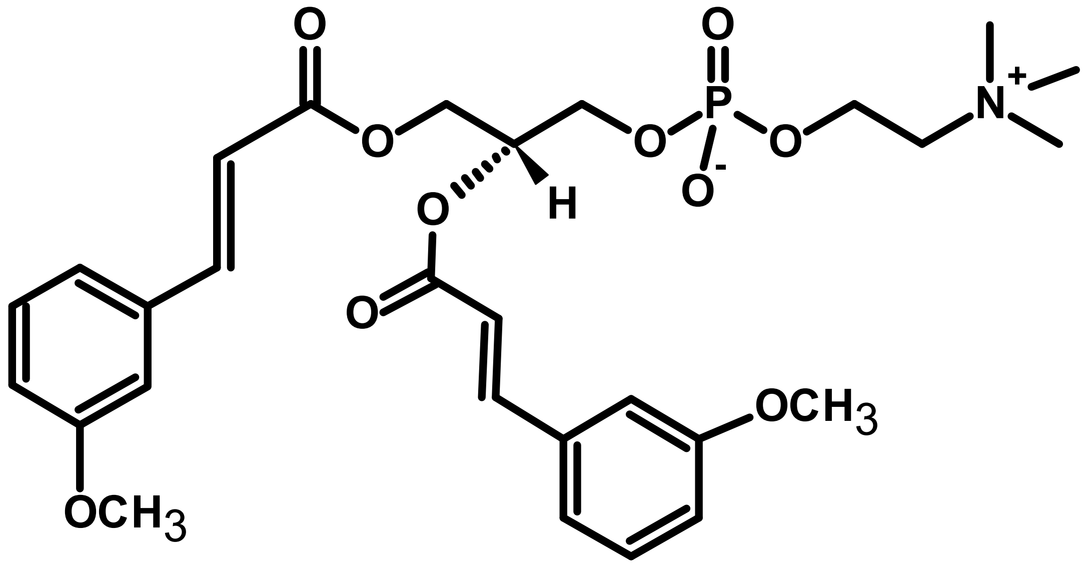

1,2-di(3-methoxycinnamoyl)-*sn*-glycero-3-phosphocholine (**3b**)

Figure S6: ^{1}H NMR spectrum of 3b.

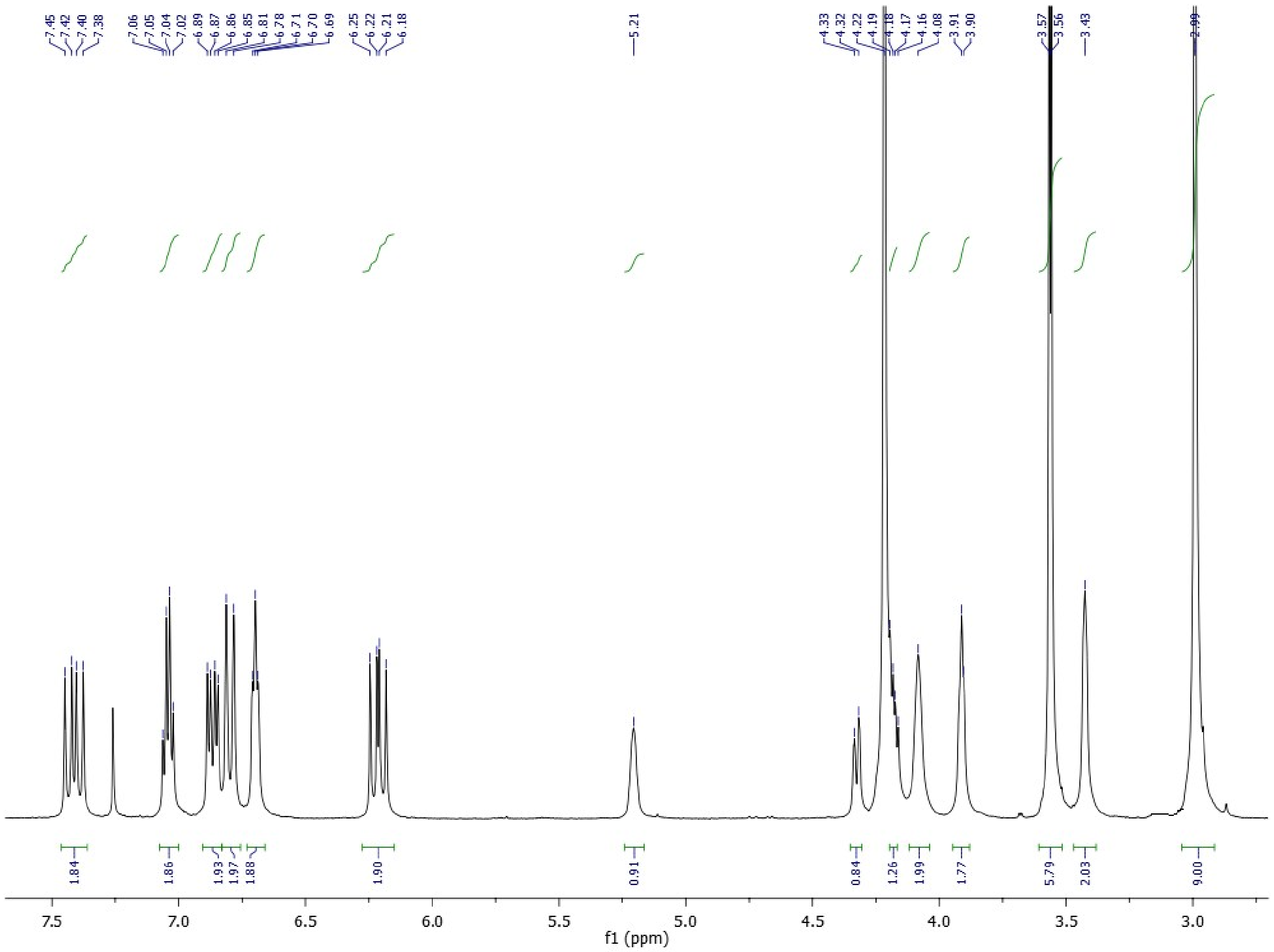

Figure S7: ^{13}C NMR spectrum of 3b.

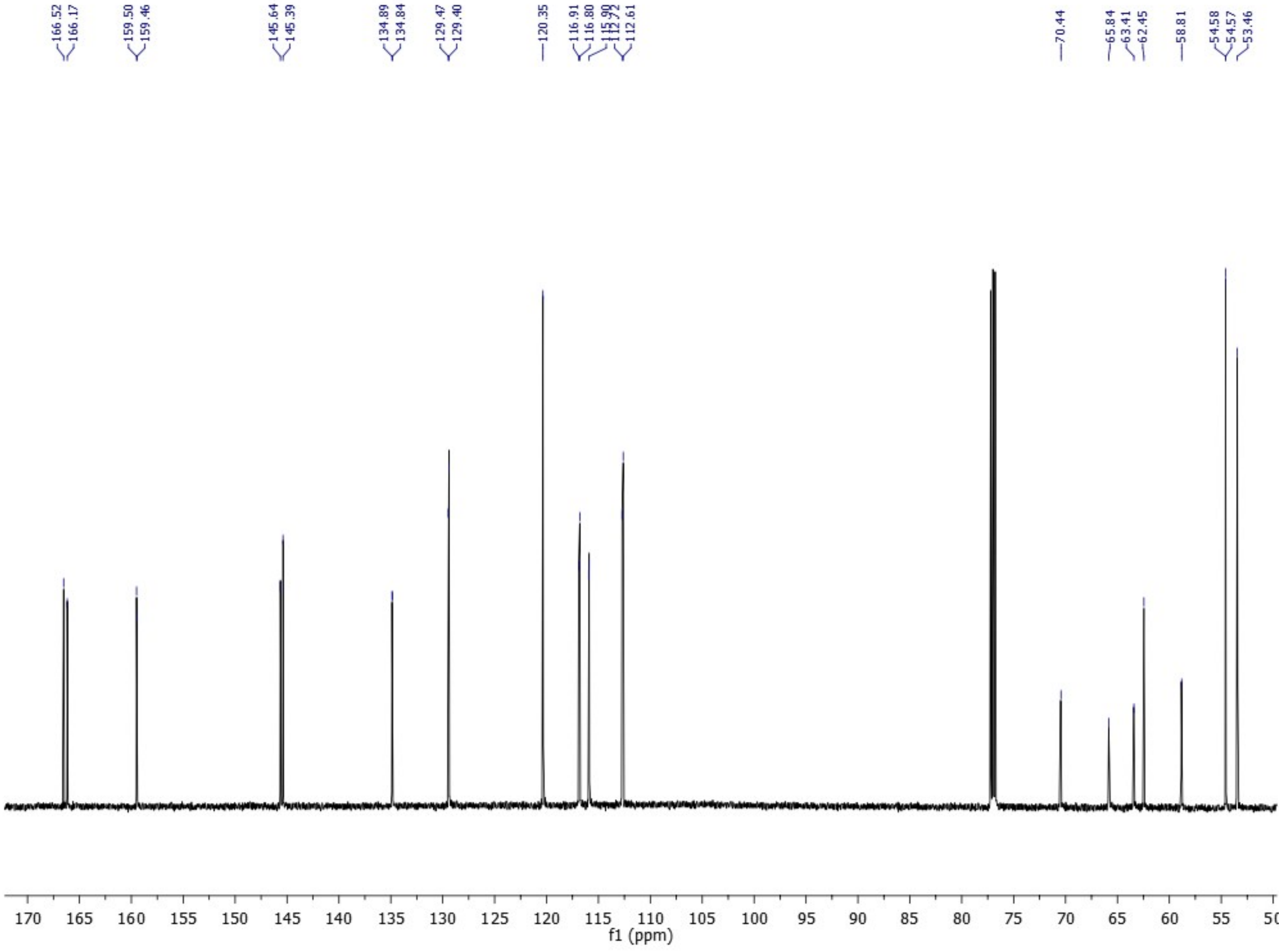

Figure S8: ^{31}P NMR spectrum of 3b.

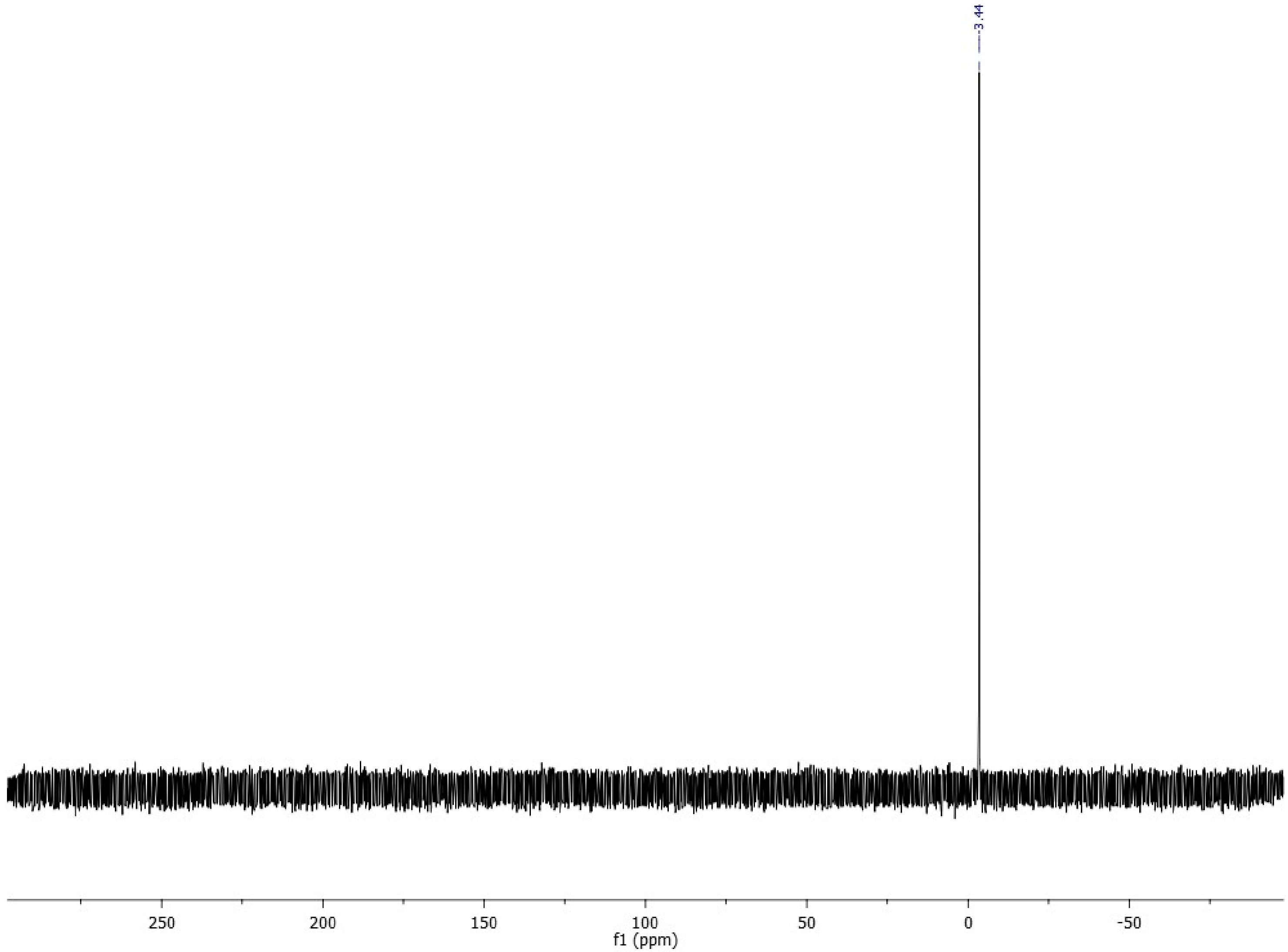

Figure S9: ^{1}H – ^{1}H COSY spectrum of 3b.

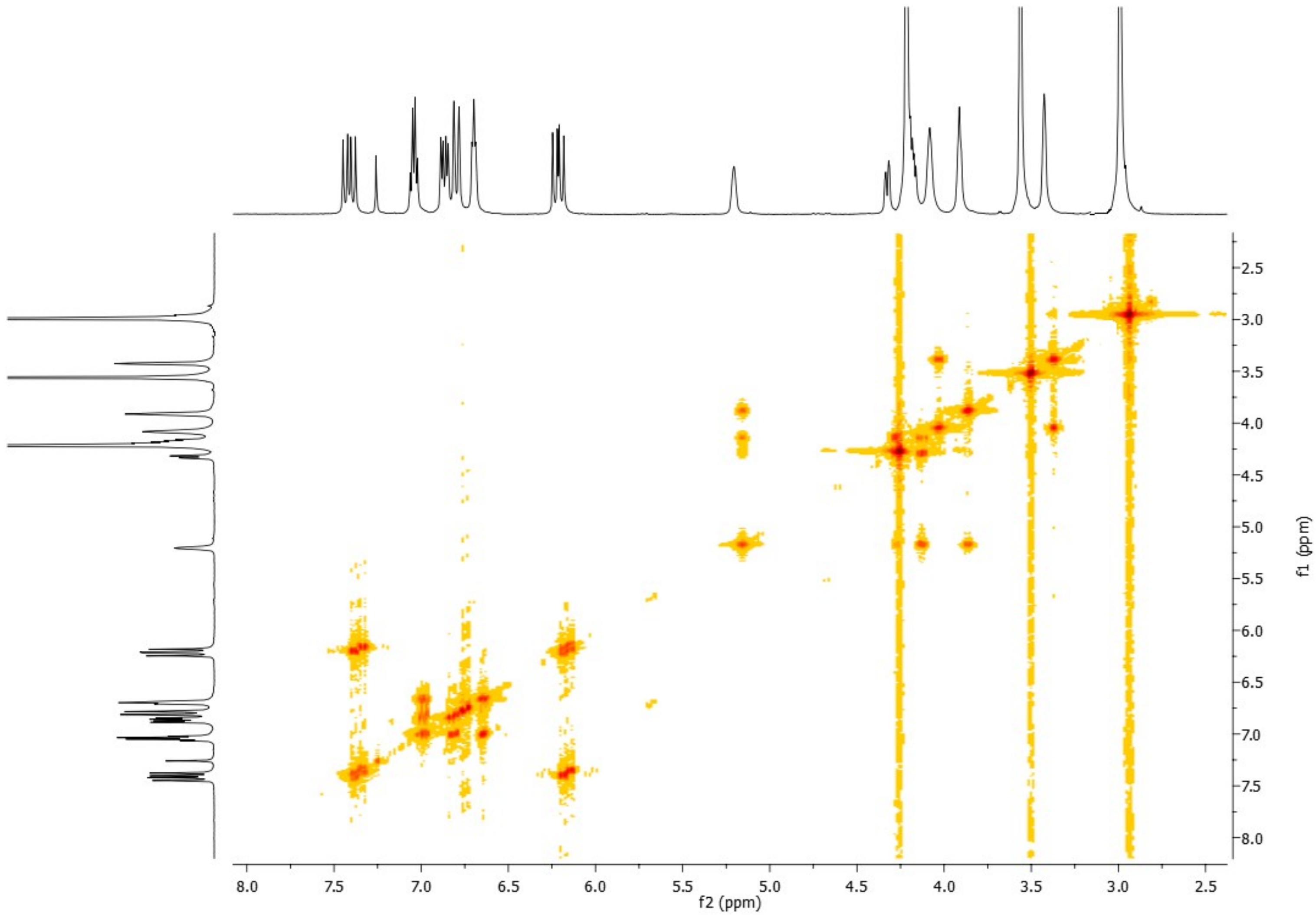

Figure S10: HSQC spectrum of 3b.

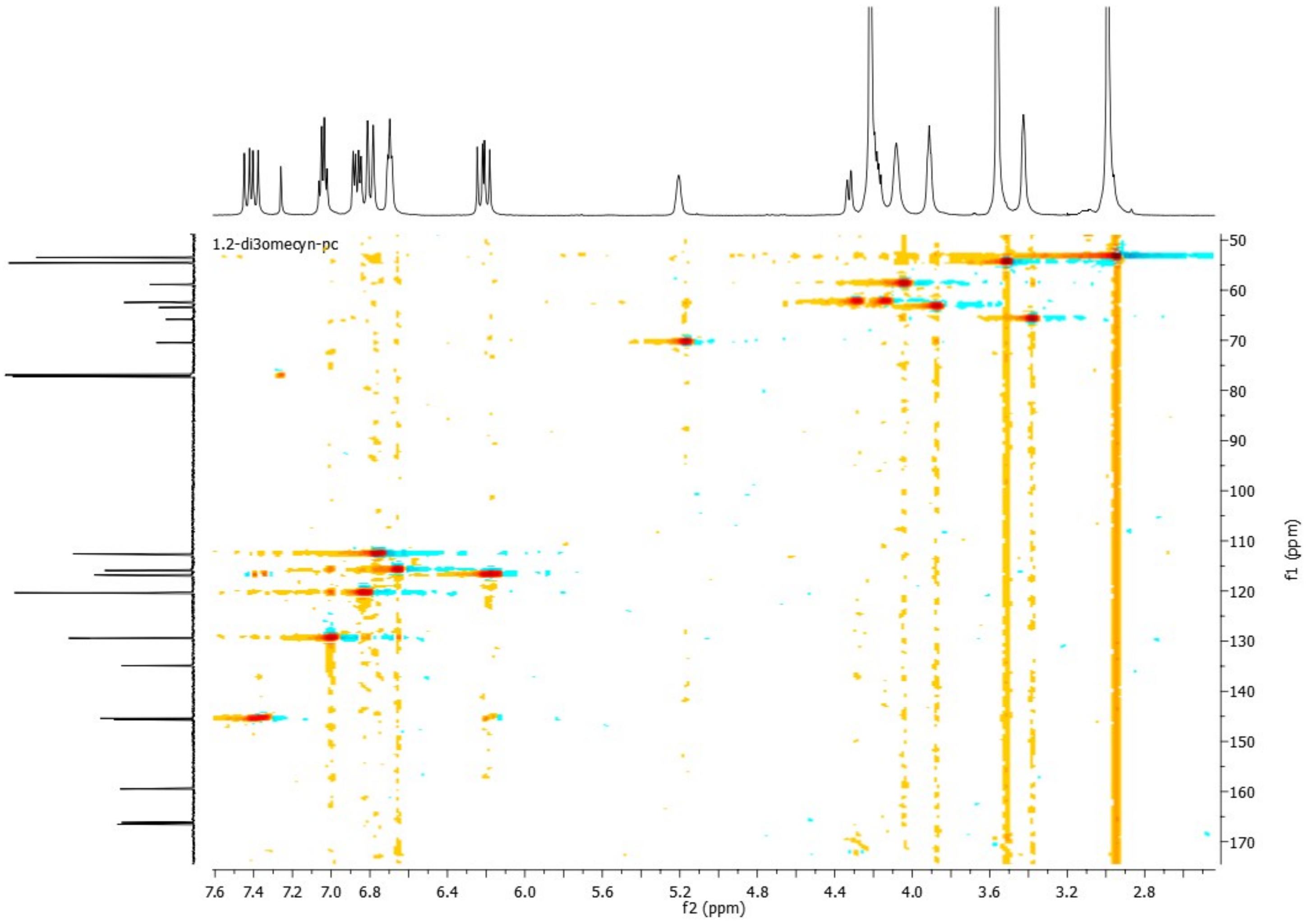

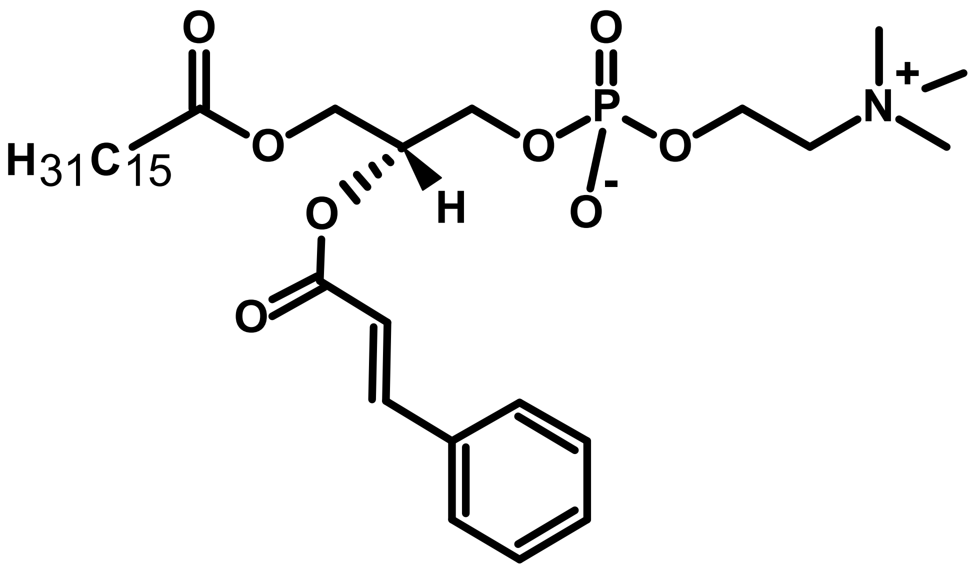

1-palmitoyl-2-cinnamoyl-*sn*-glycero-3-phosphocholine (**5a**)

Figure S11: ^{1}H NMR spectrum of 5a.

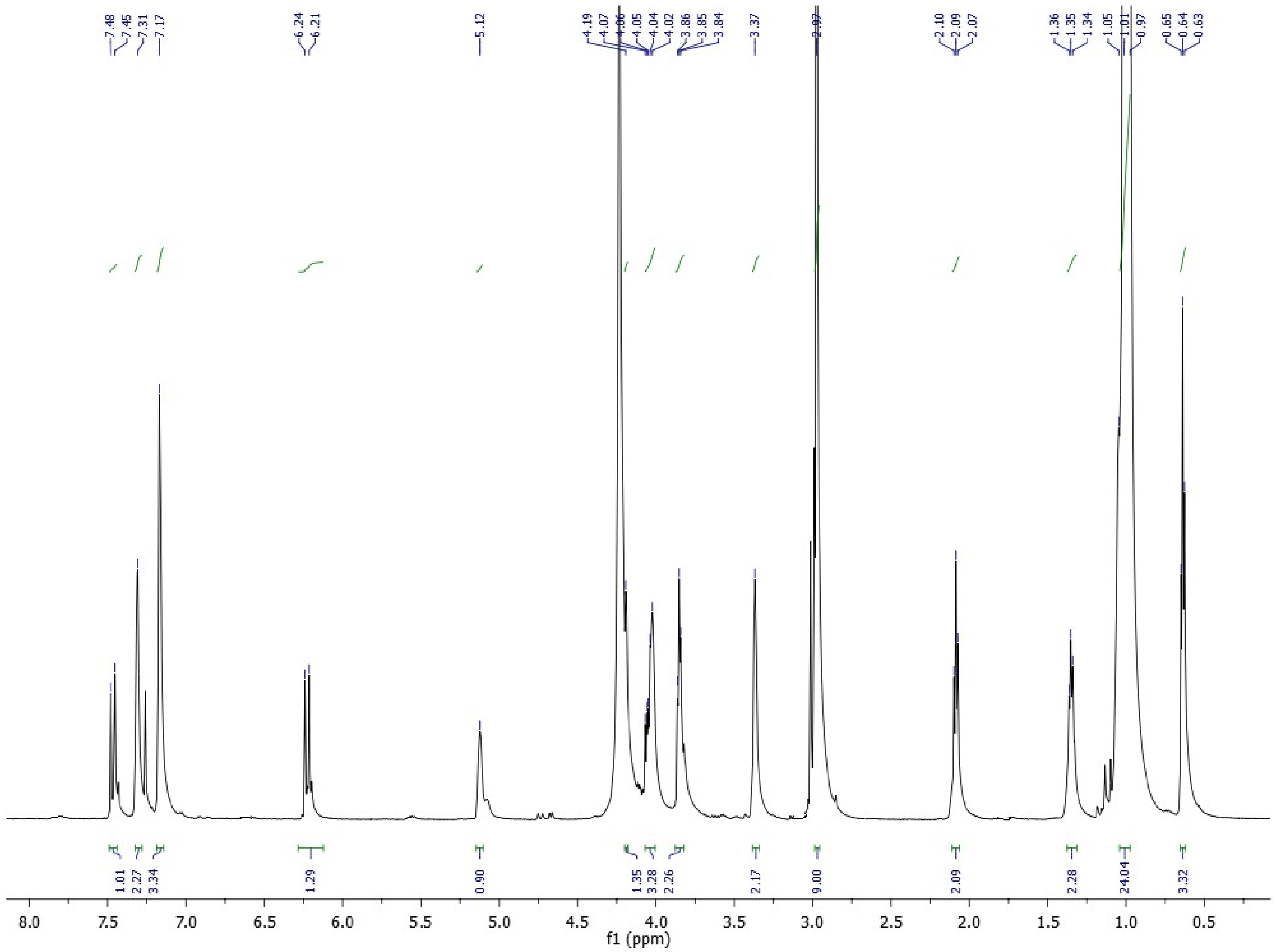

Figure S12: ^{13}C NMR spectrum of 5a.

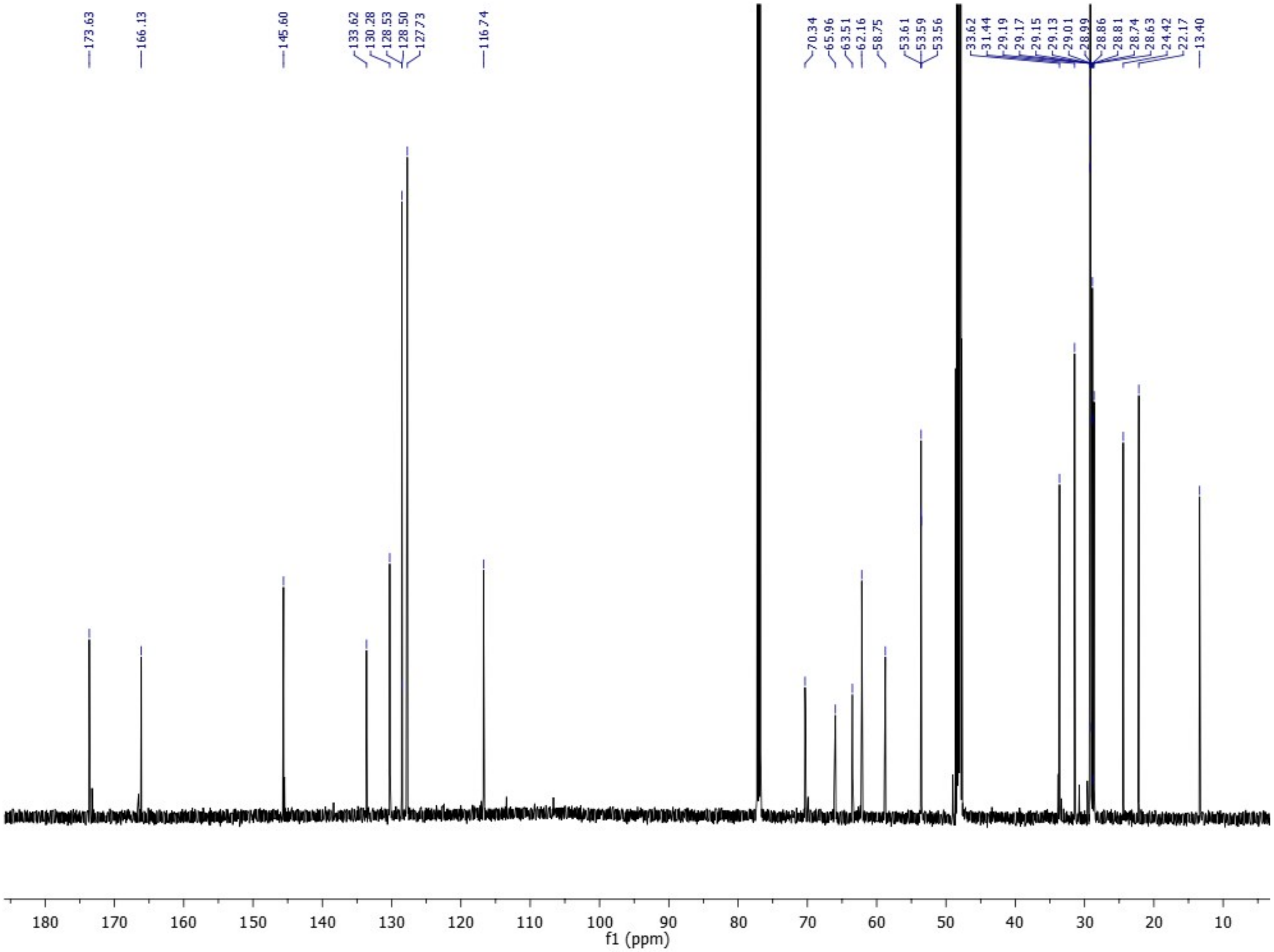

Figure S13: ^{31}P NMR spectrum of 5a.

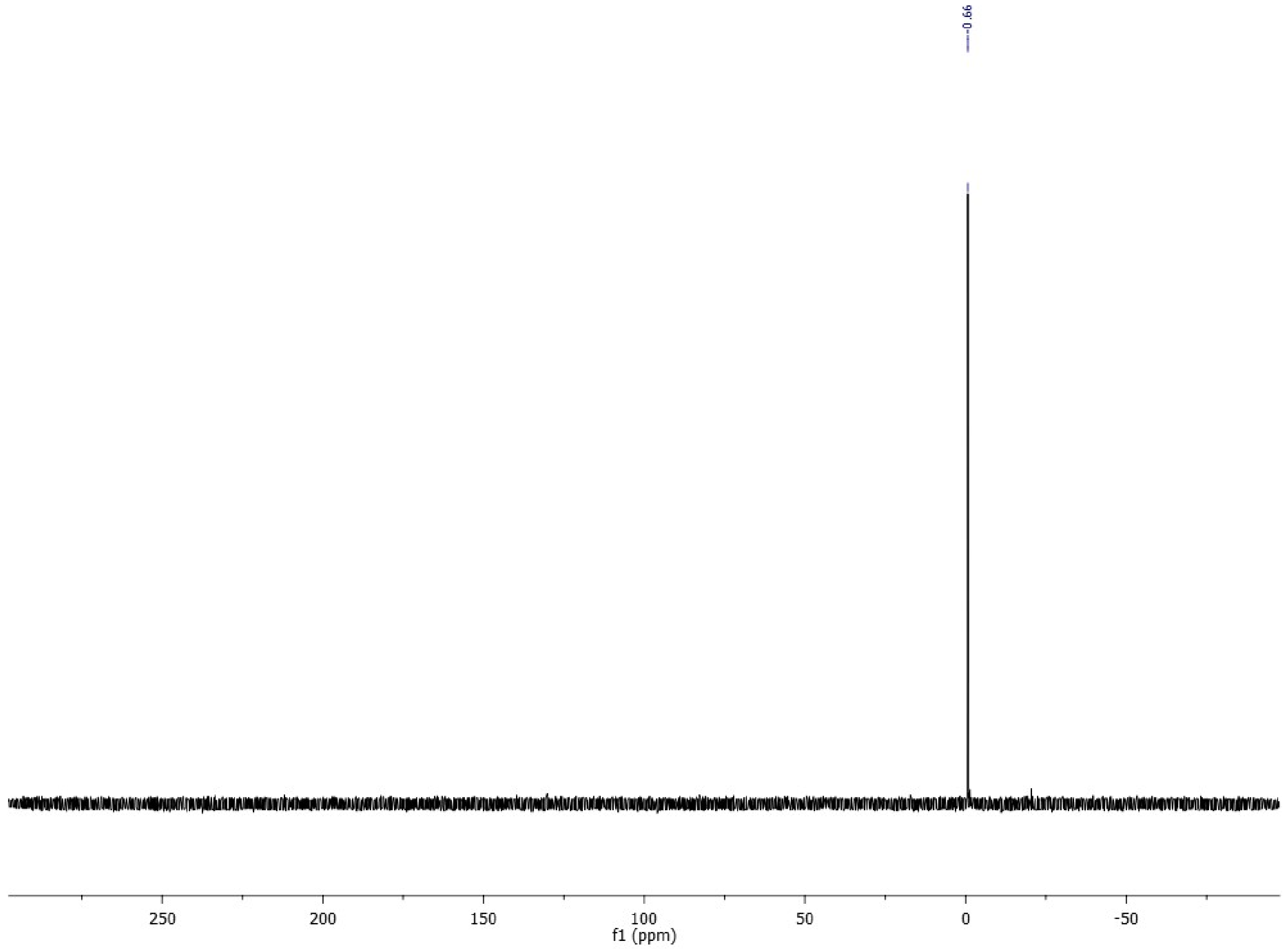

Figure S14: ^{1}H – ^{1}H COSY spectrum of 5a.

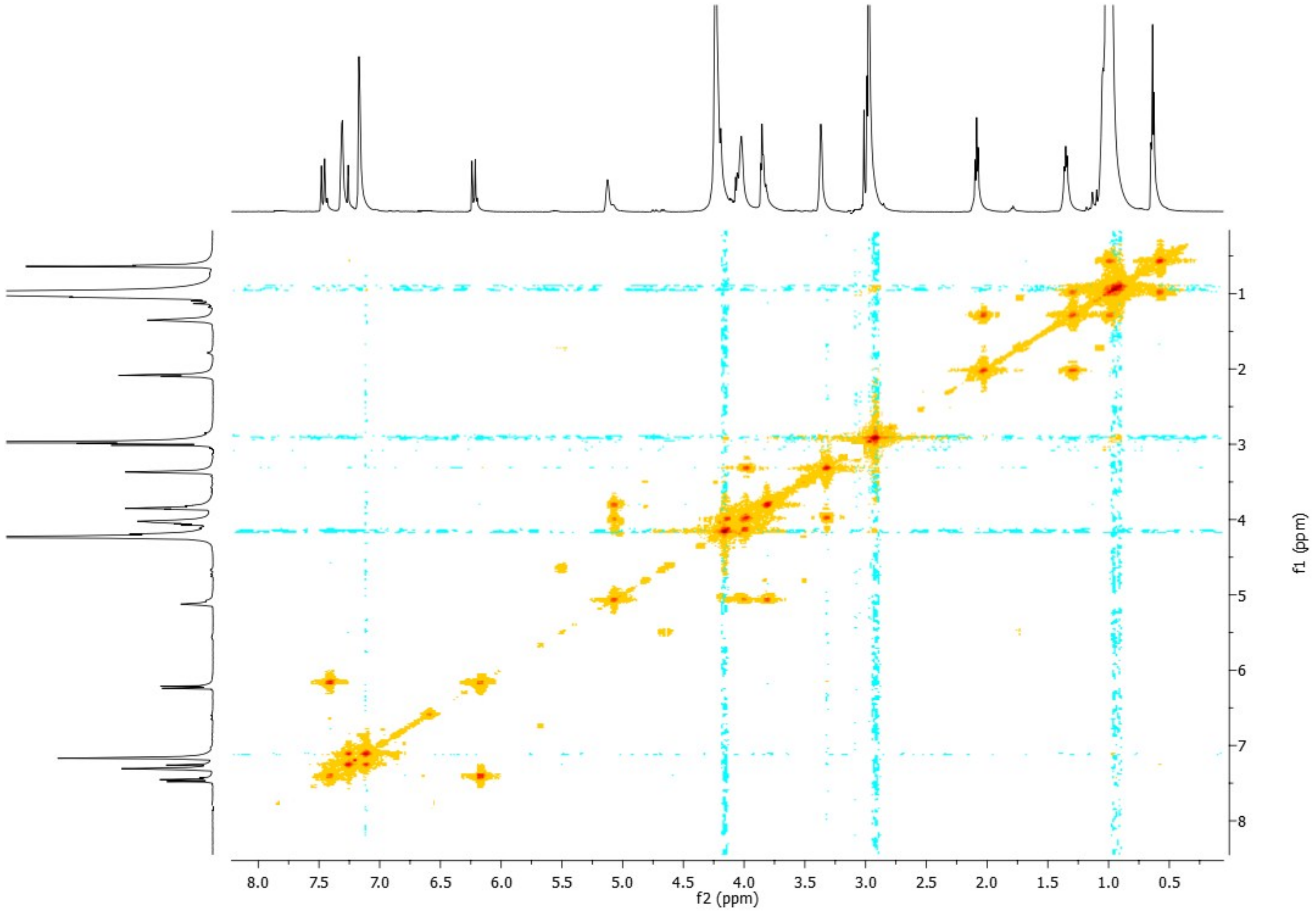

Figure S15: HSQC spectrum of 5a.

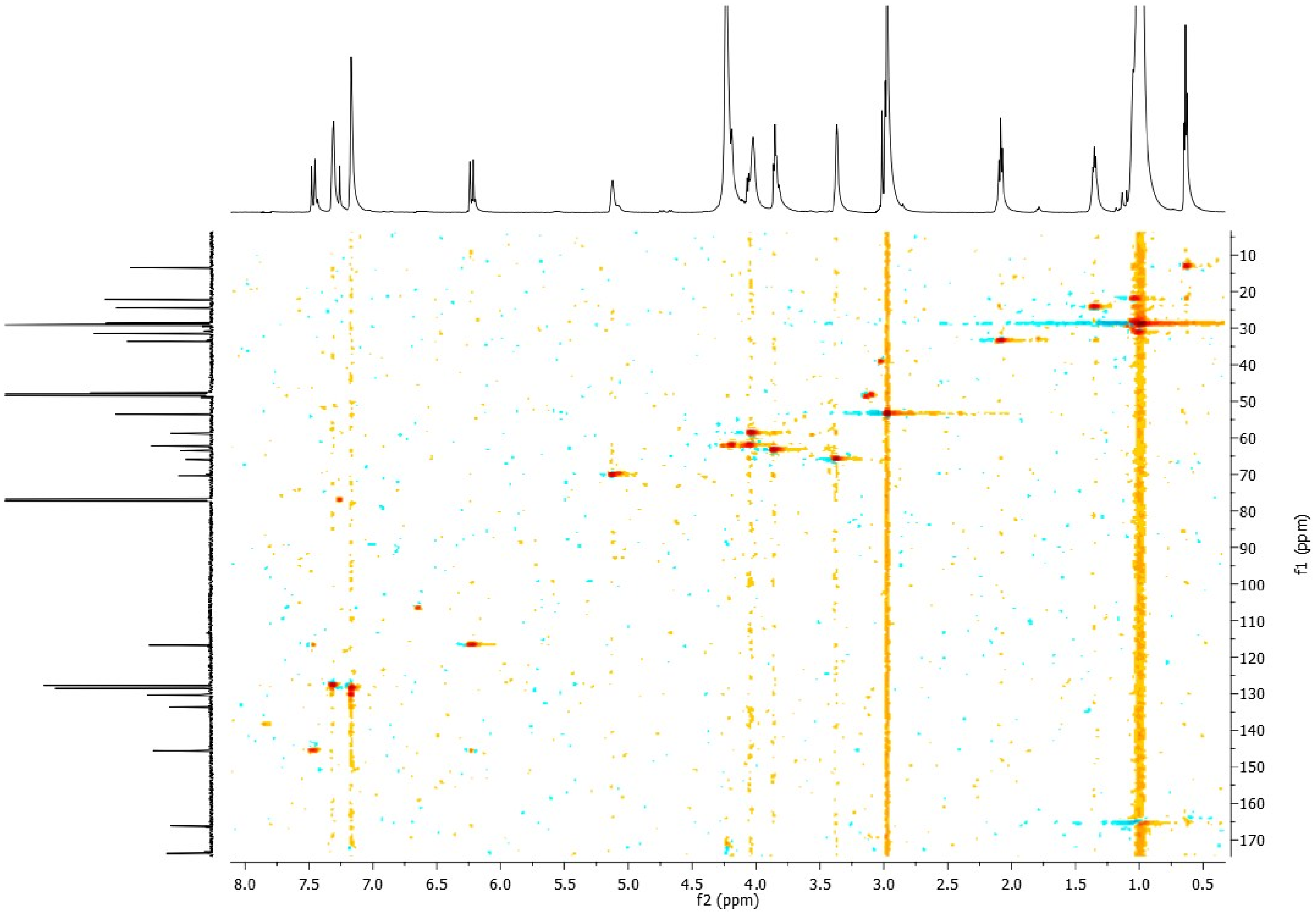

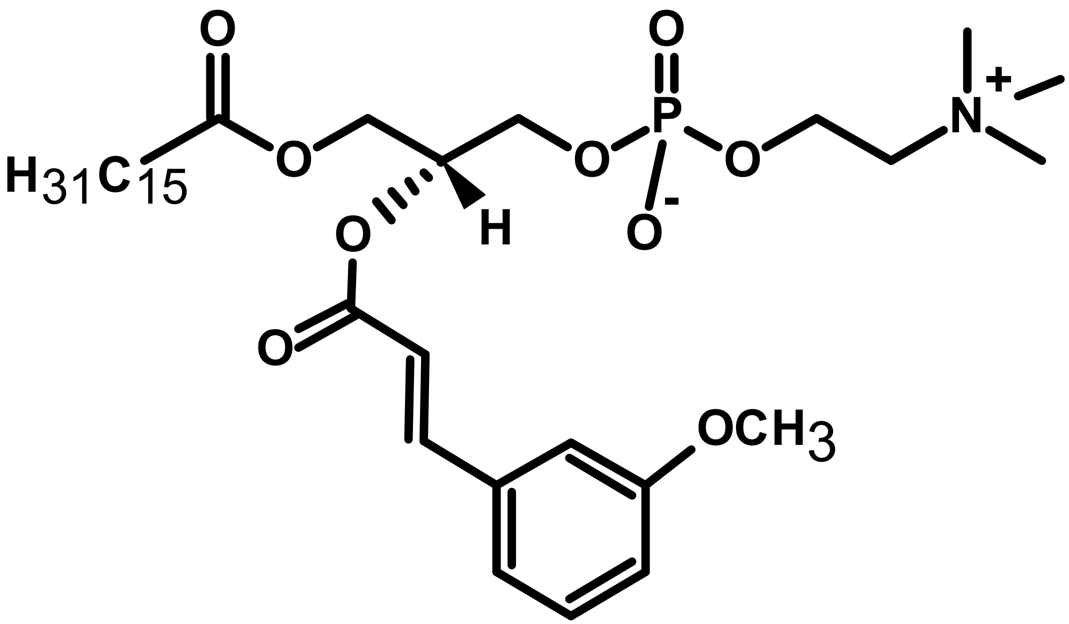

1-palmitoyl-2-(3-methoxycinnamoyl)-*sn*-glycero-3-phosphocholine (**5b**)

Figure S16: ^{1}H NMR spectrum of 5b.

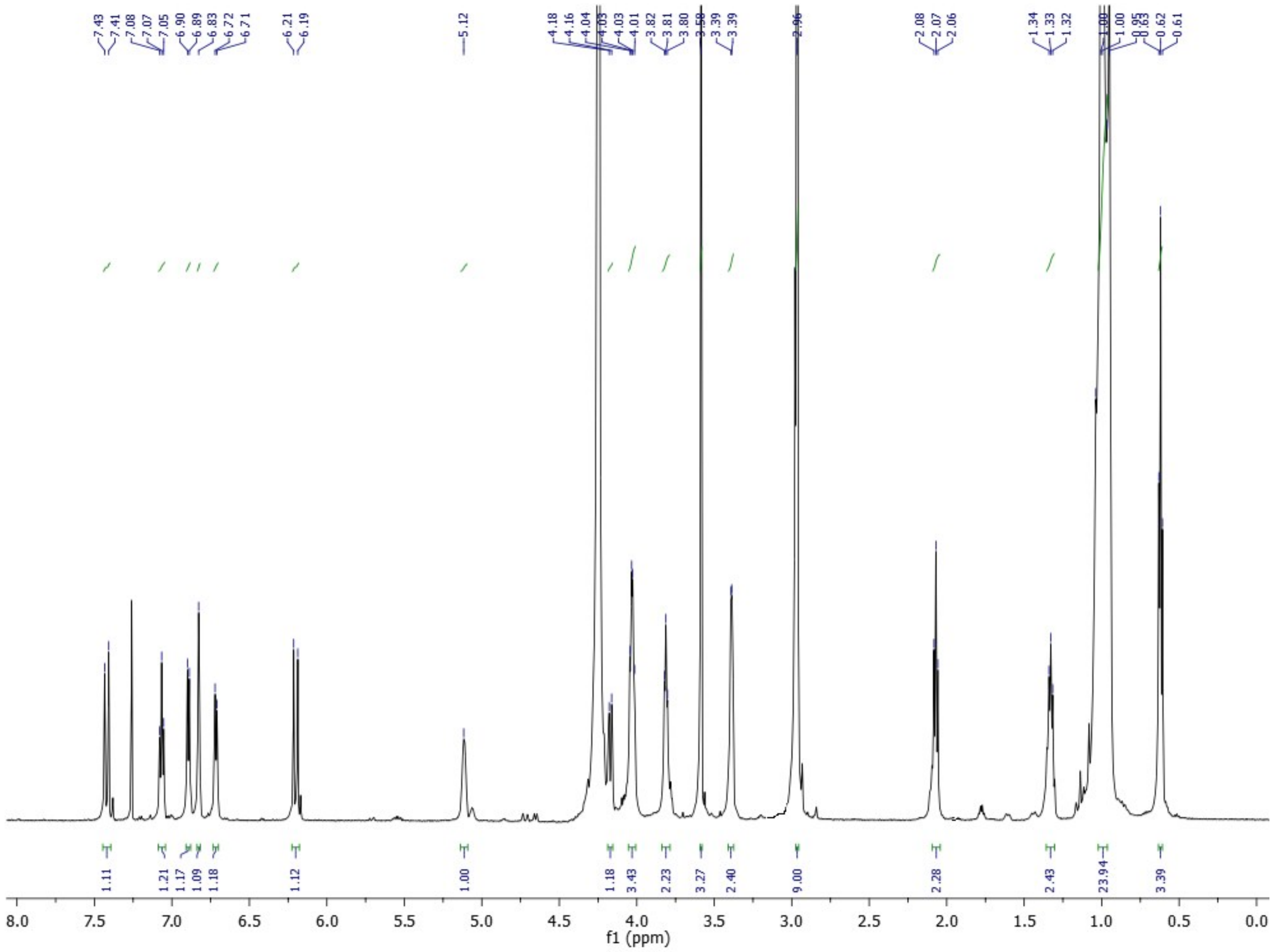

Figure S17: ¹³C NMR spectrum of 5b.

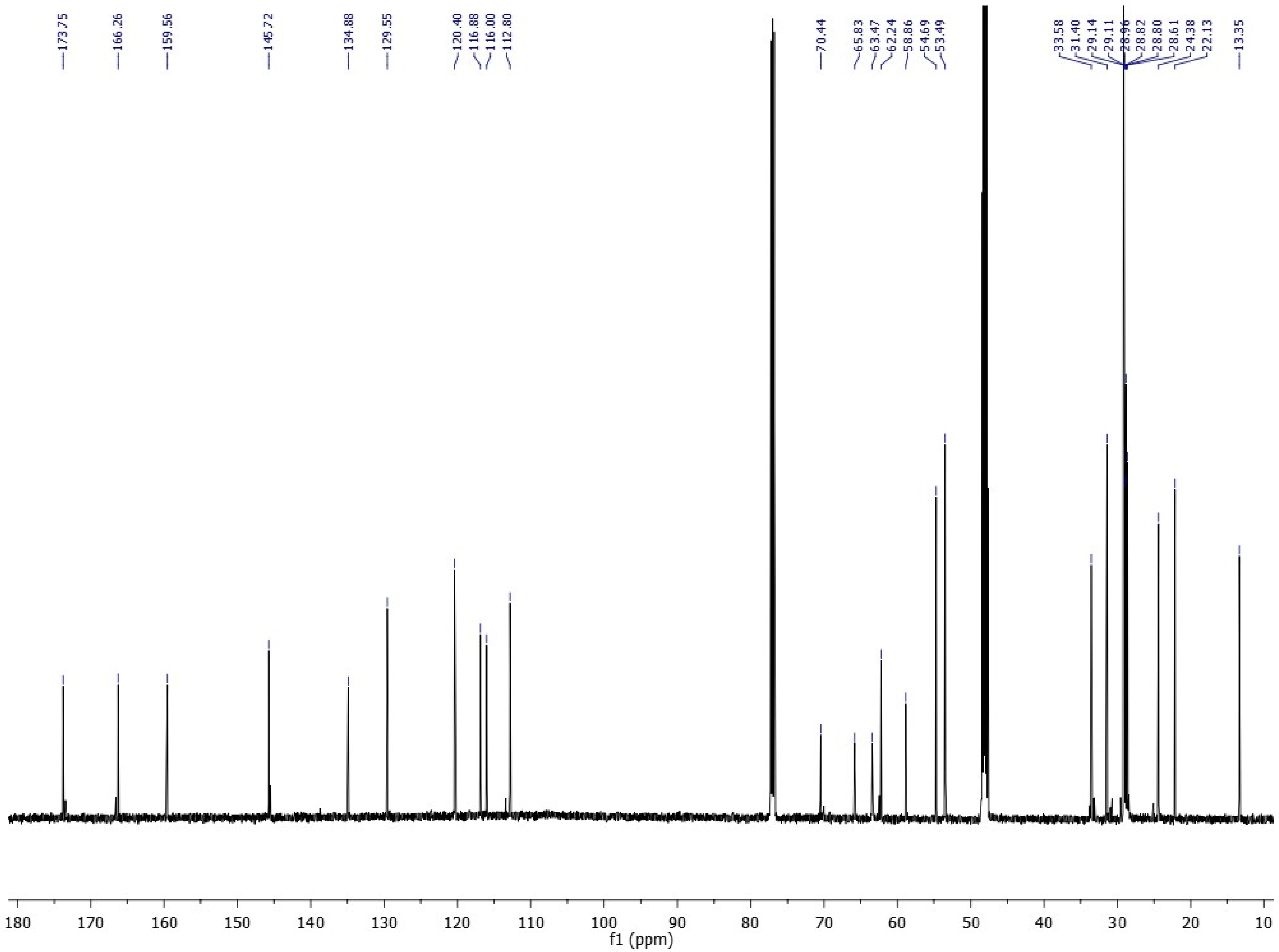

Figure S18: ^{31}P NMR spectrum of 5b.

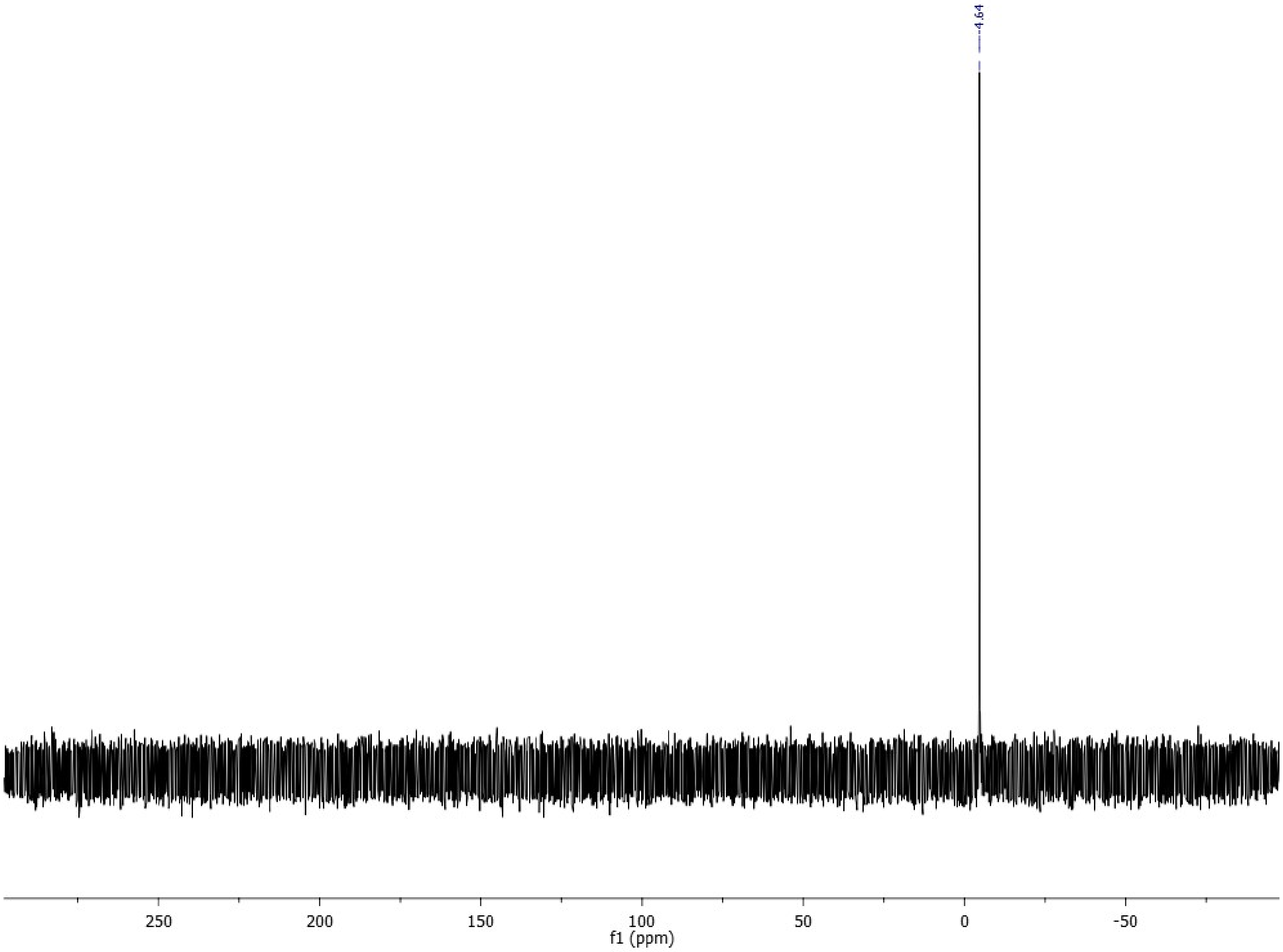

Figure S19: ^{1}H – ^{1}H COSY spectrum of 5b.

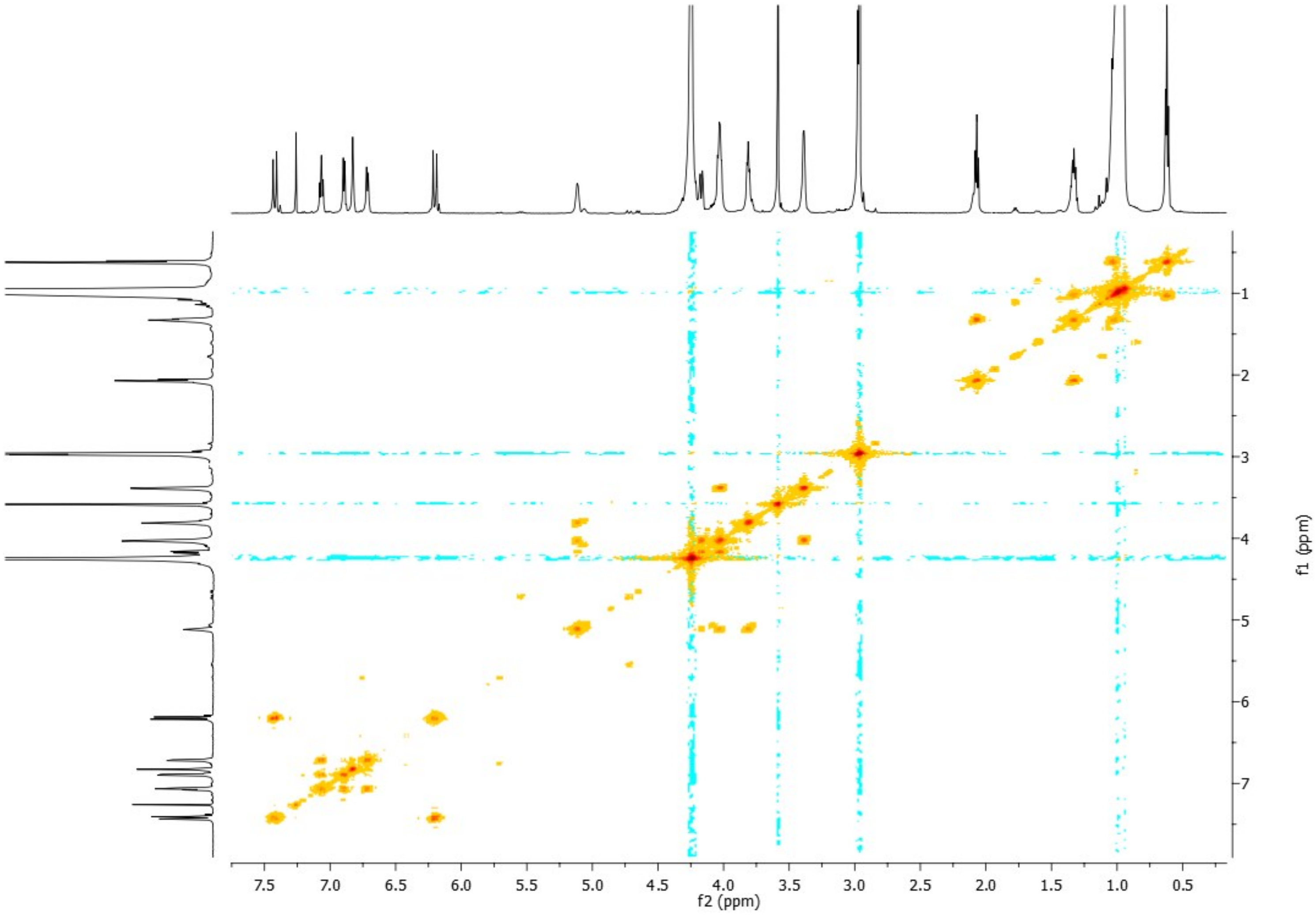

Figure S20: HSQC spectrum of 5b.

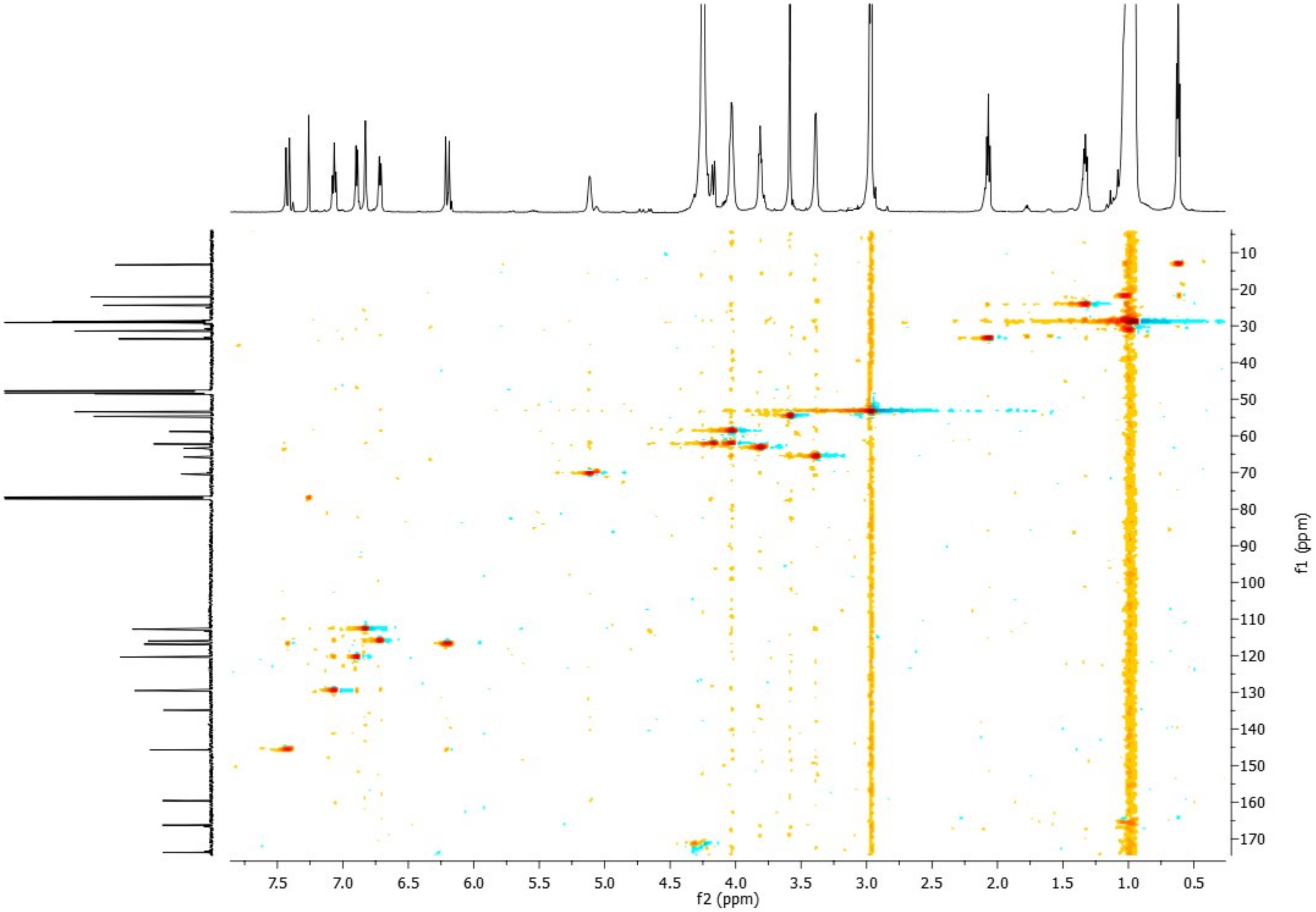

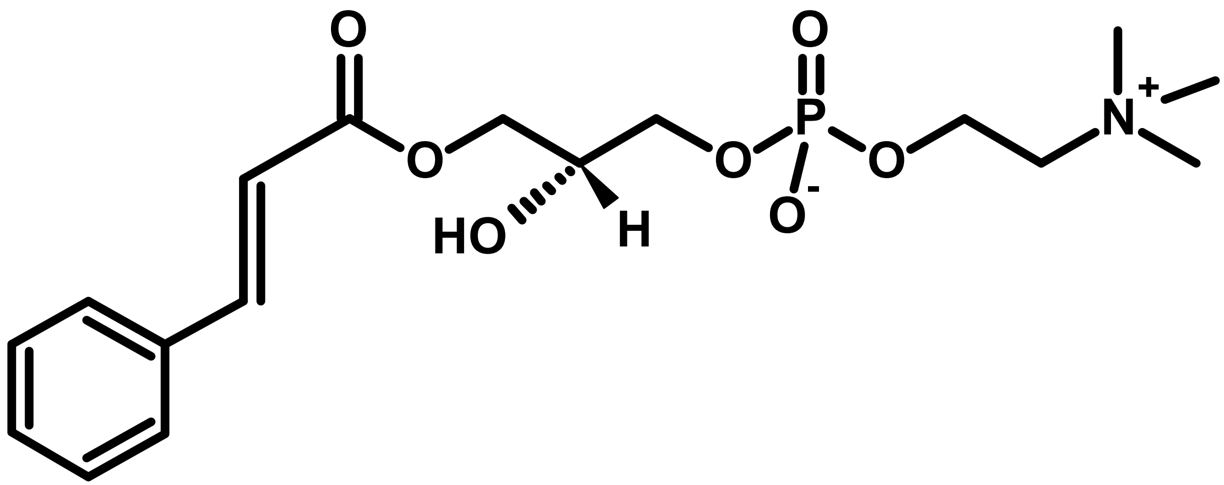

1-cinnamoyl-2-hydroxy-*sn*-glycero-3-phosphocholine (**9a**)

Figure S21: ^{1}H NMR spectrum of 9a.

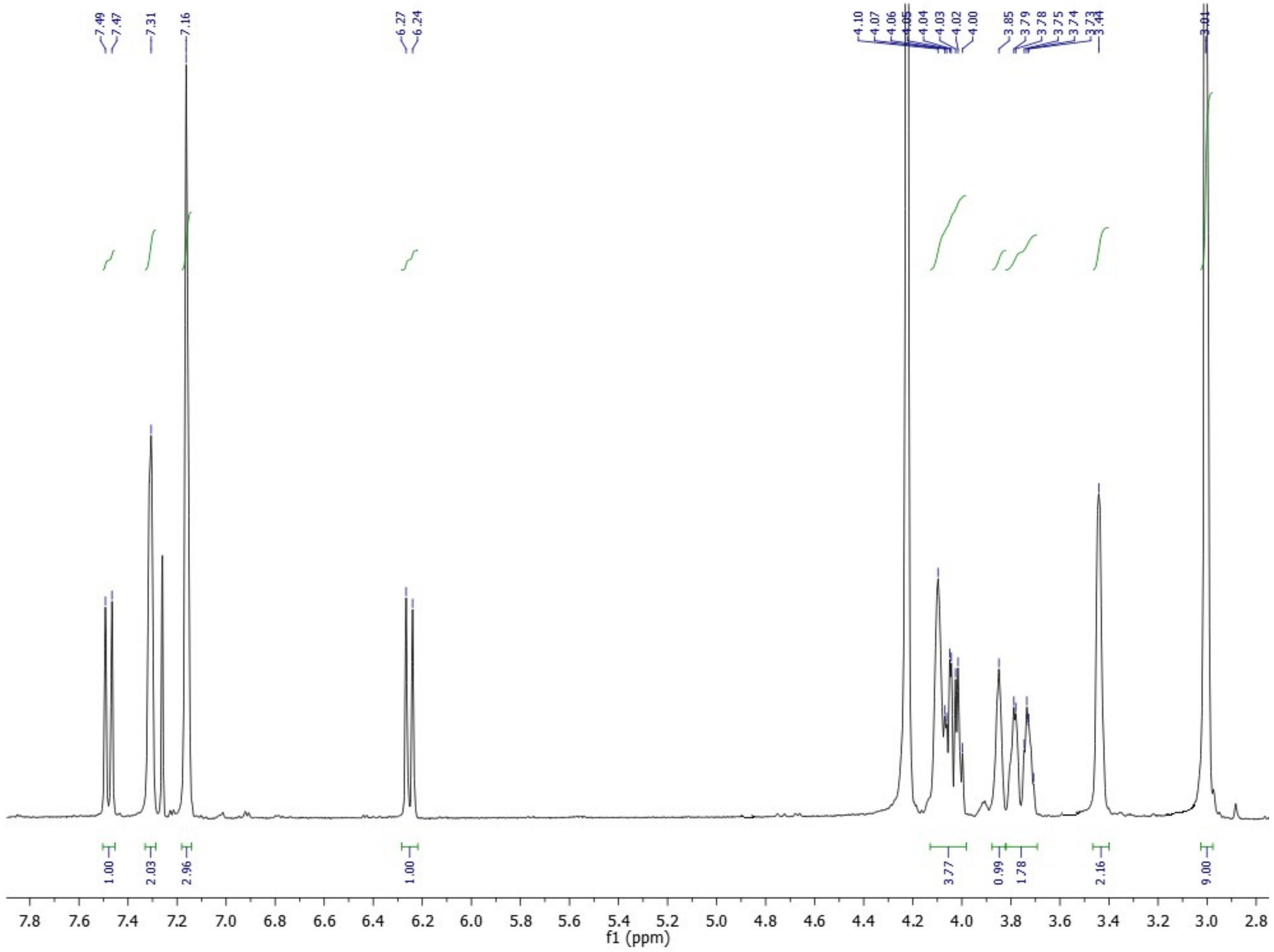

Figure S22: ^{13}C NMR spectrum of 9a.

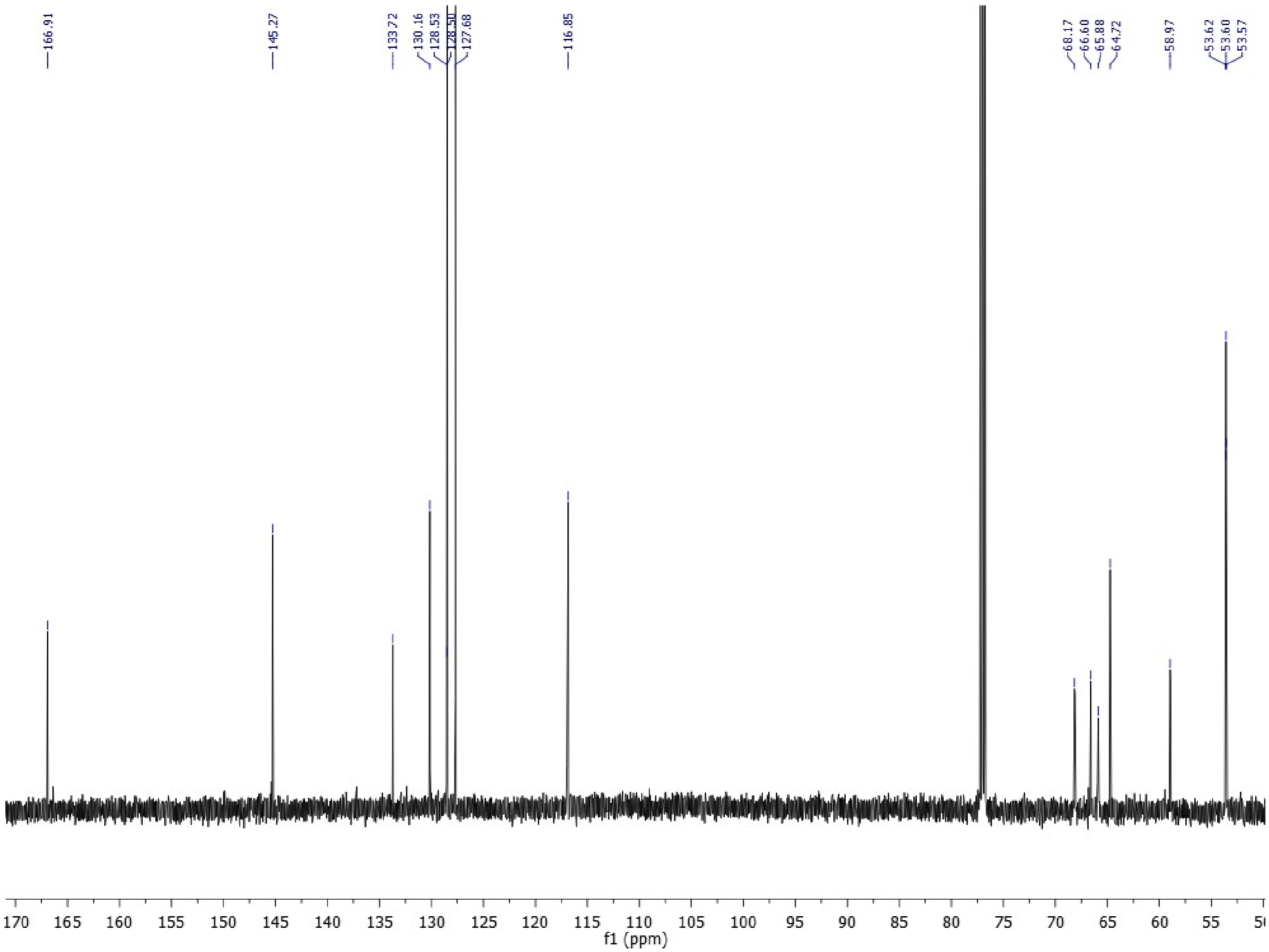

Figure S23: ^{31}P NMR spectrum of 9a.

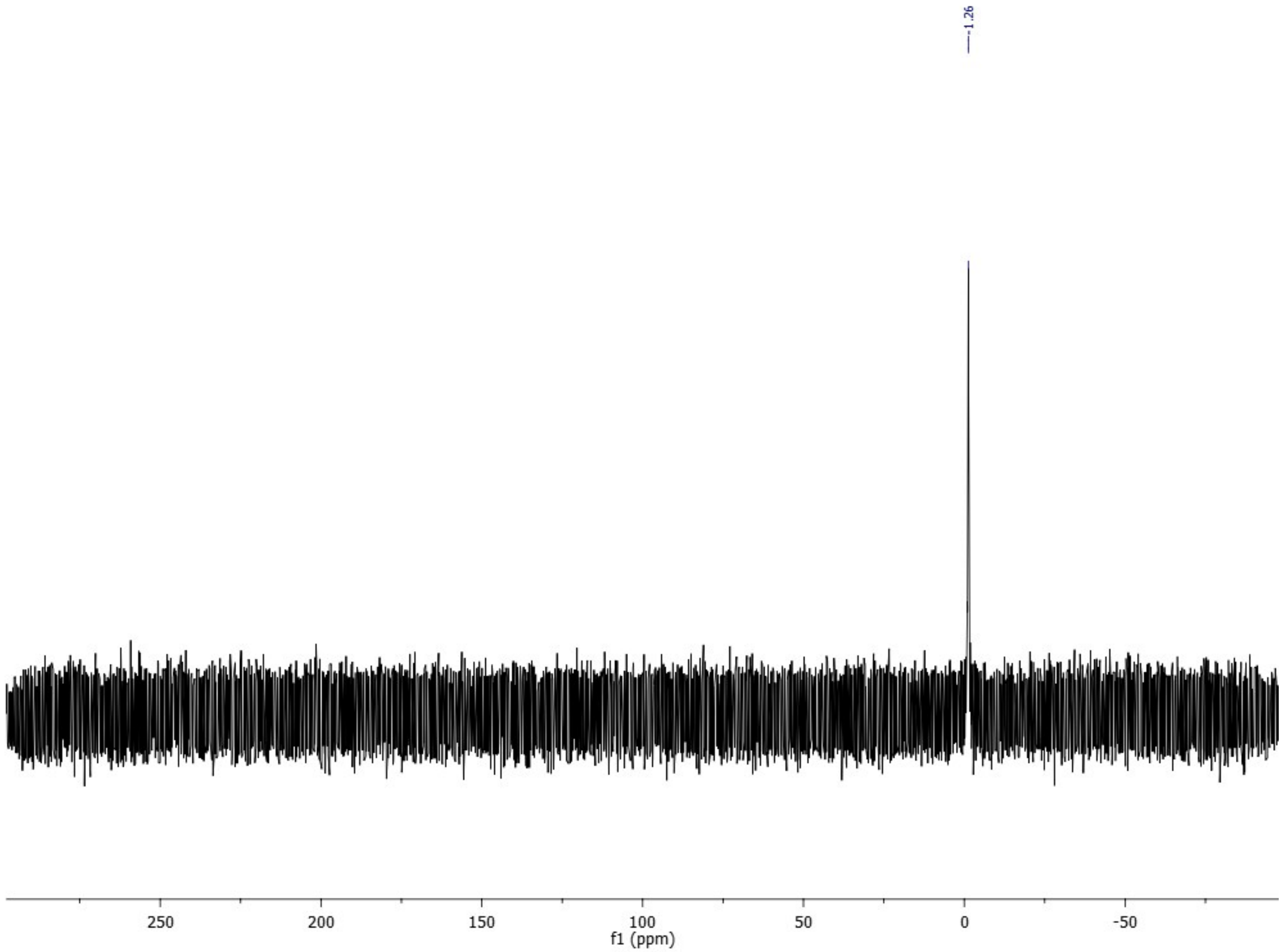

Figure S24: ^{1}H – ^{1}H COSY spectrum of 9a.

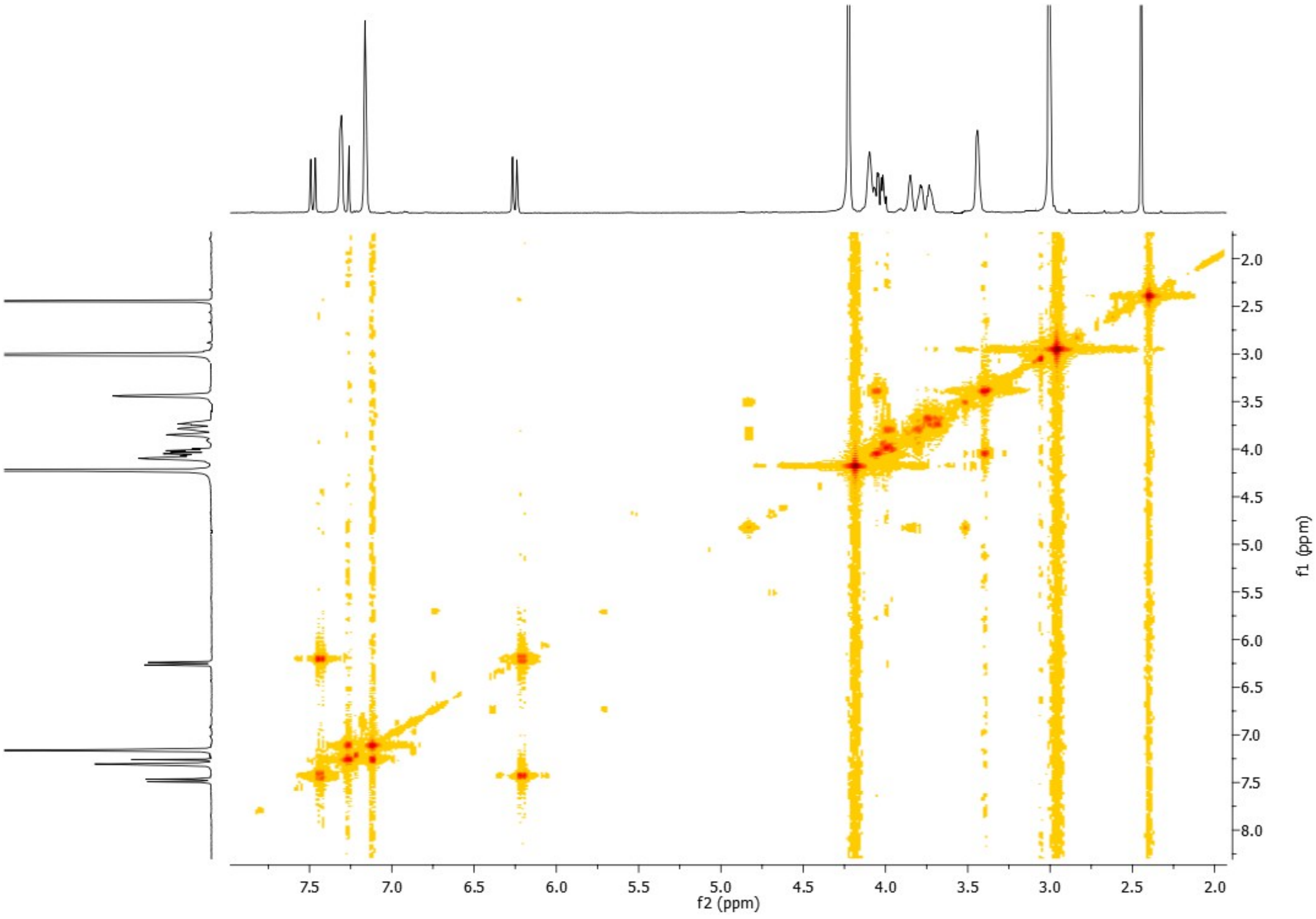

Figure S25: HSQC spectrum of 9a.

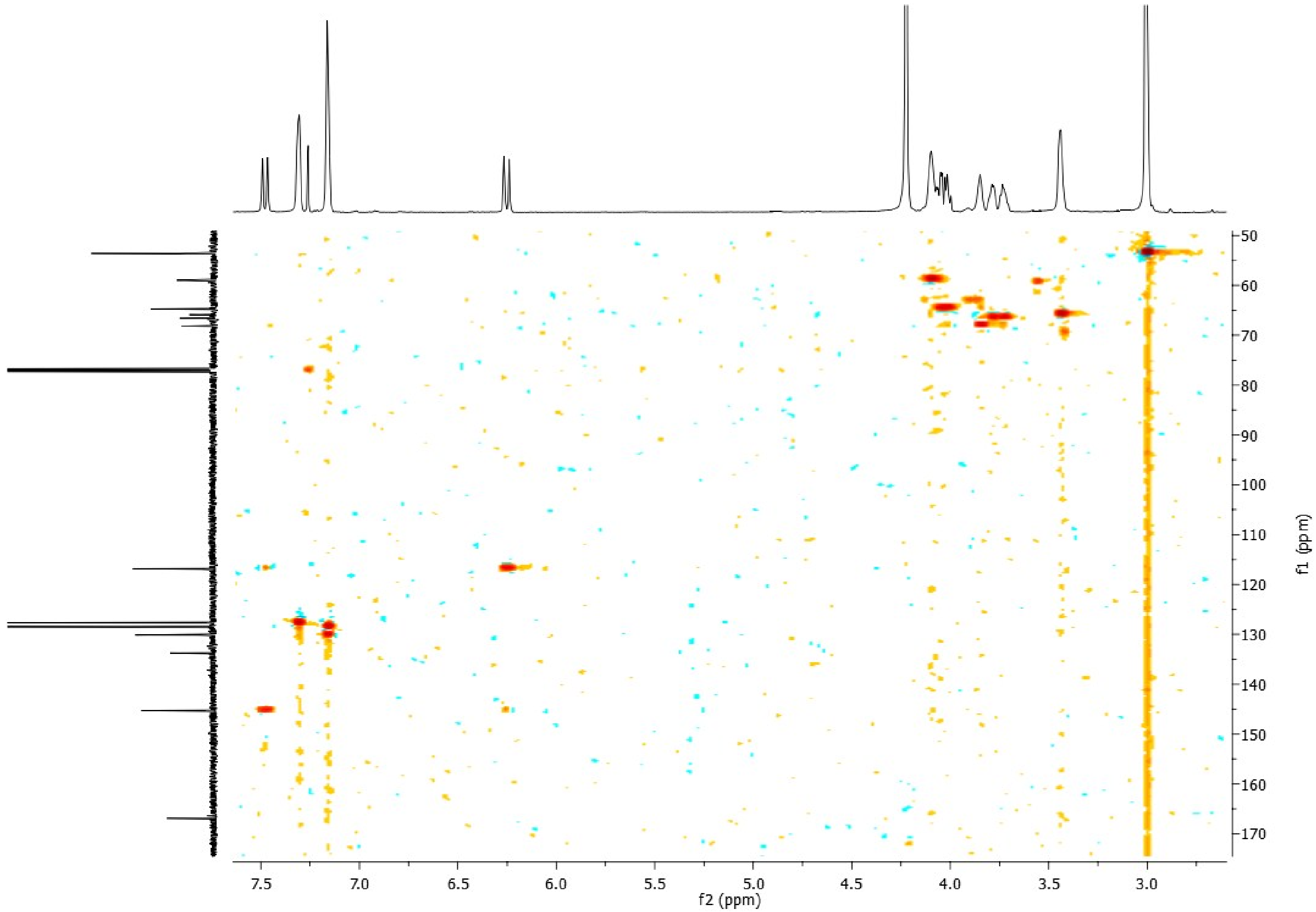

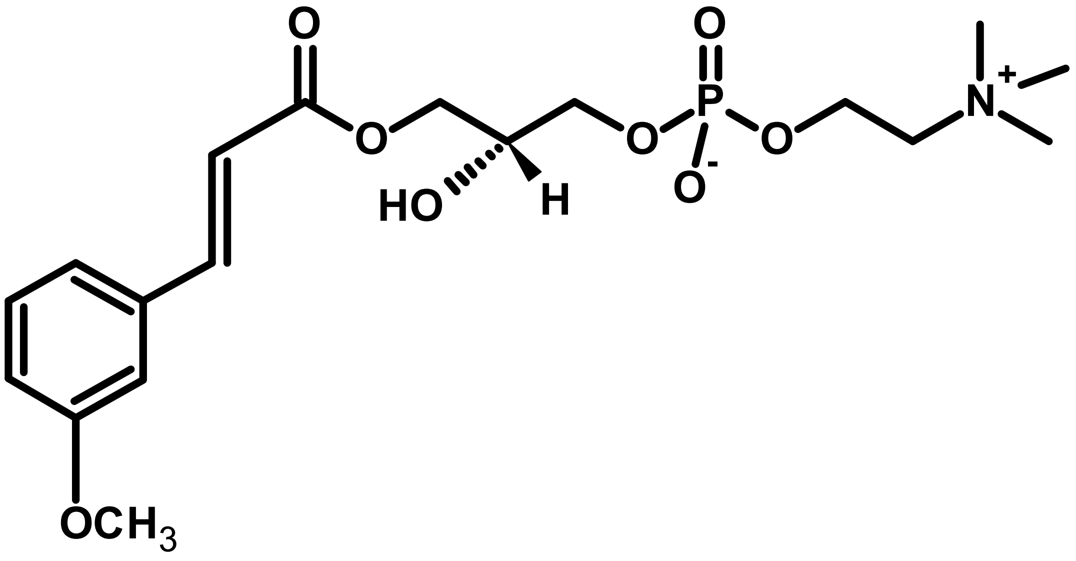

1-(3-methoxycinnamoyl)-2-hydroxy-*sn*-glycero-3-phosphocholine (**9b**)

Figure S26: ^{1}H NMR spectrum of 9b.

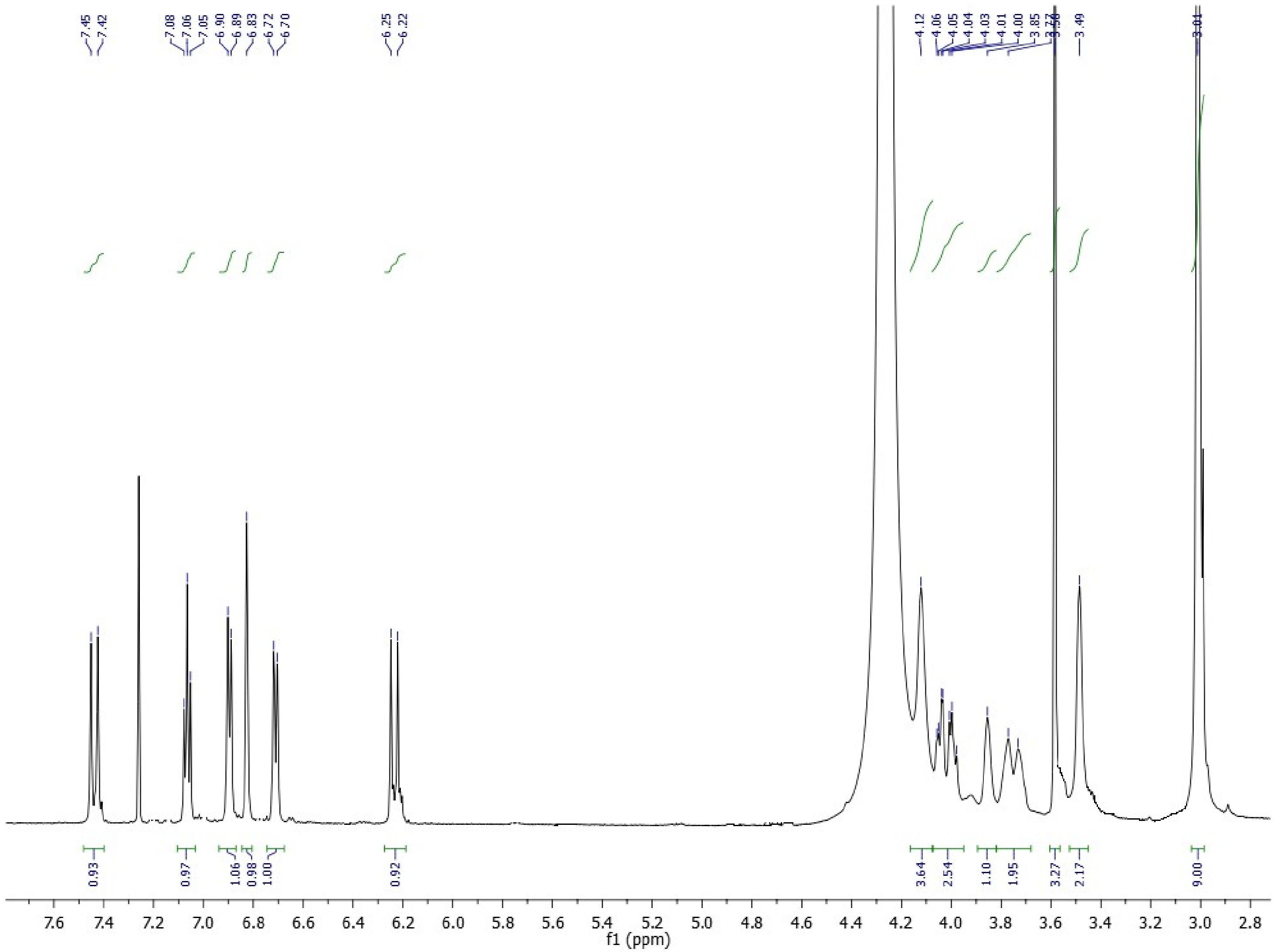

Figure S27: ^{13}C NMR spectrum of 9b.

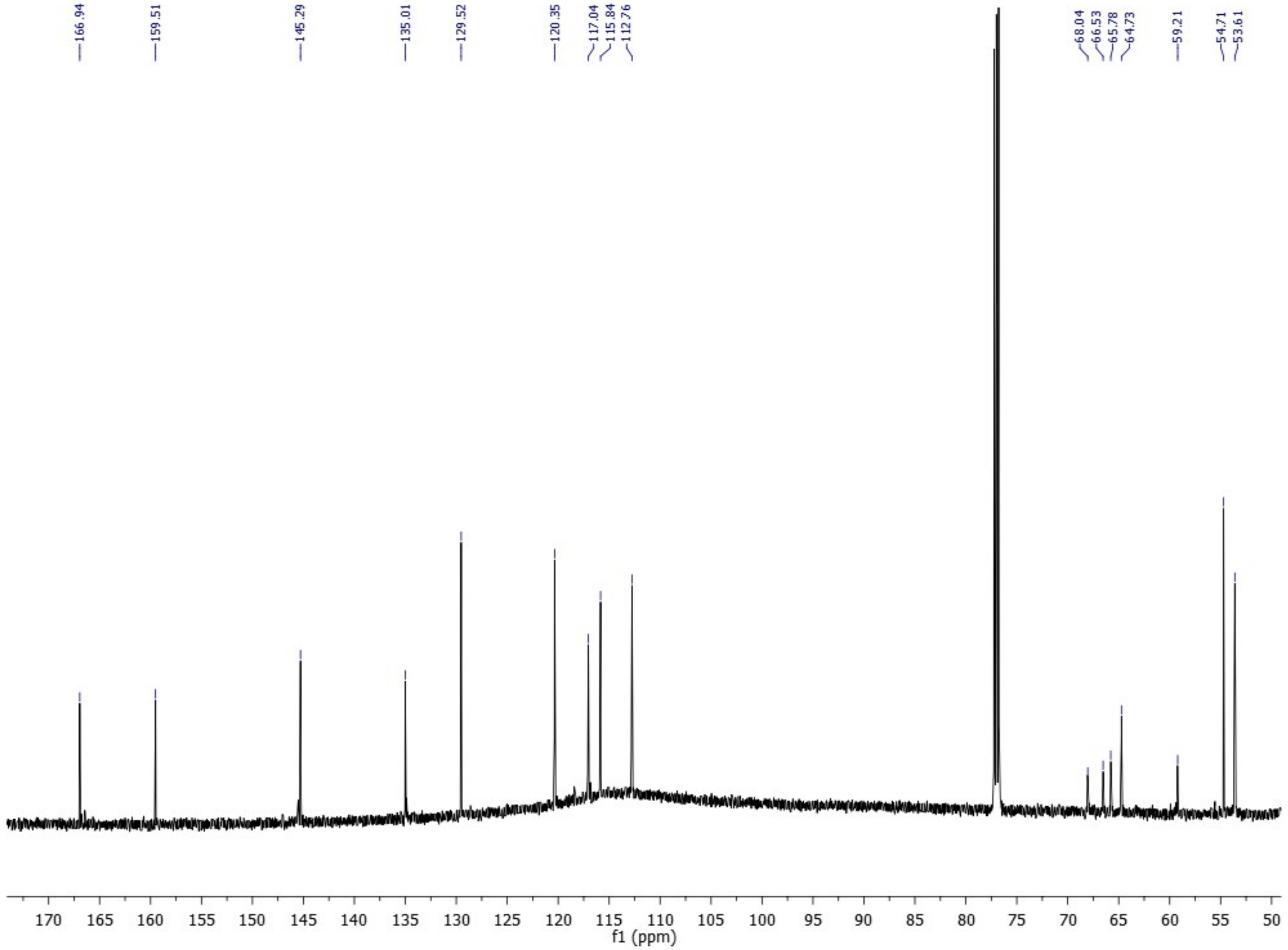

Figure S28: ^{31}P NMR spectrum of 9b.

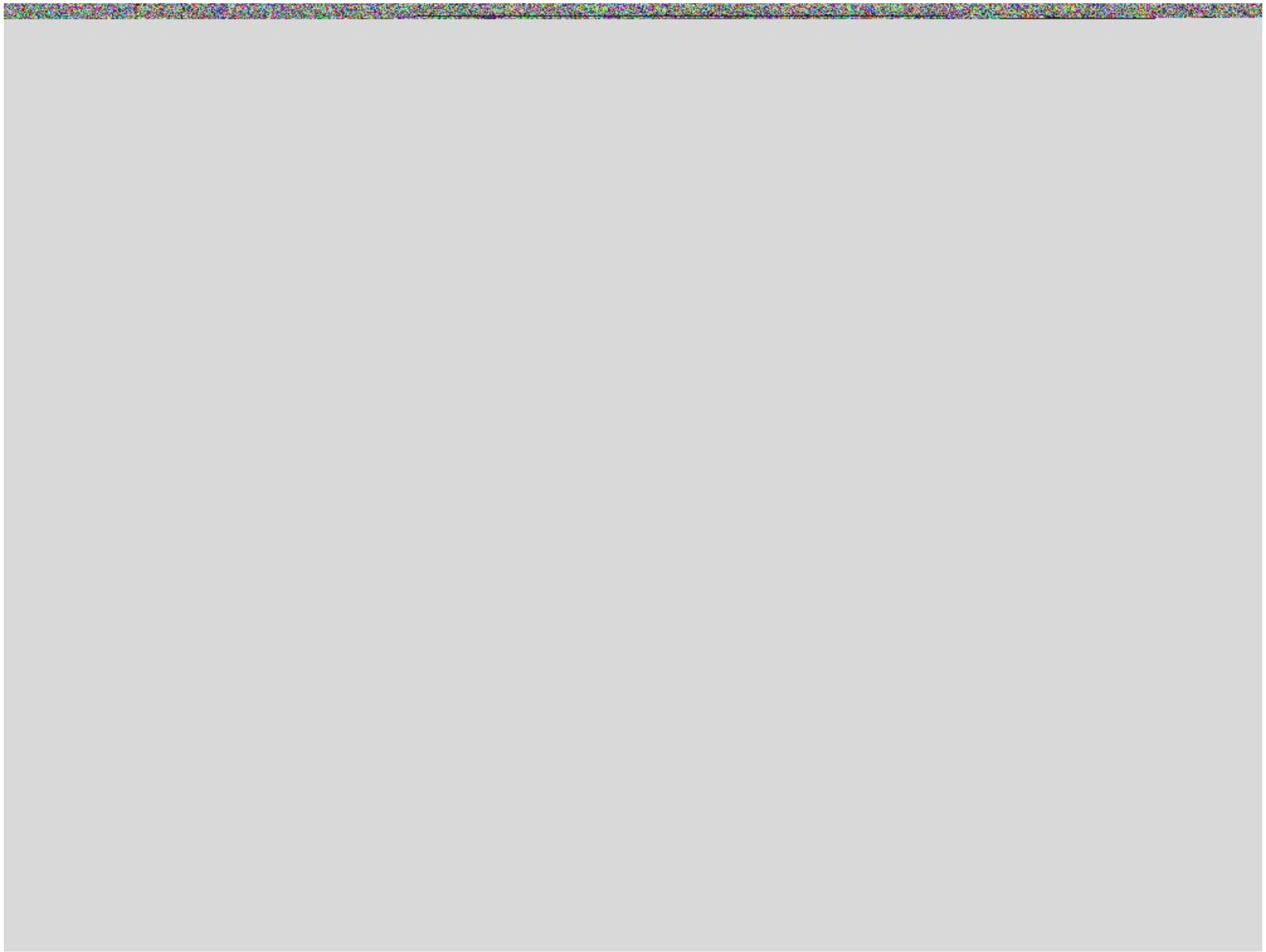

Figure S29: ^{1}H – ^{1}H COSY spectrum of 9b.

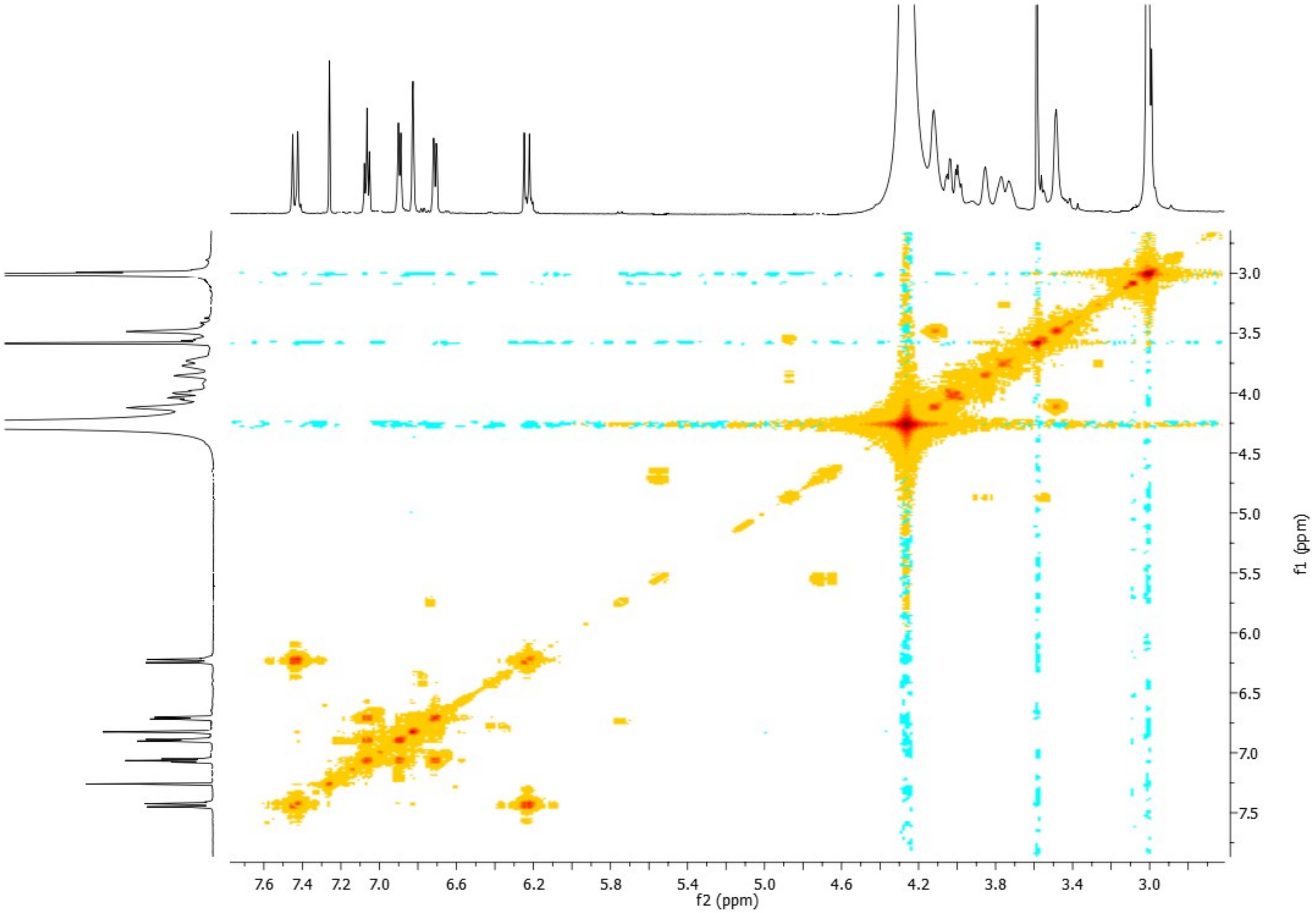

Figure S30: HSQC spectrum of 9b.

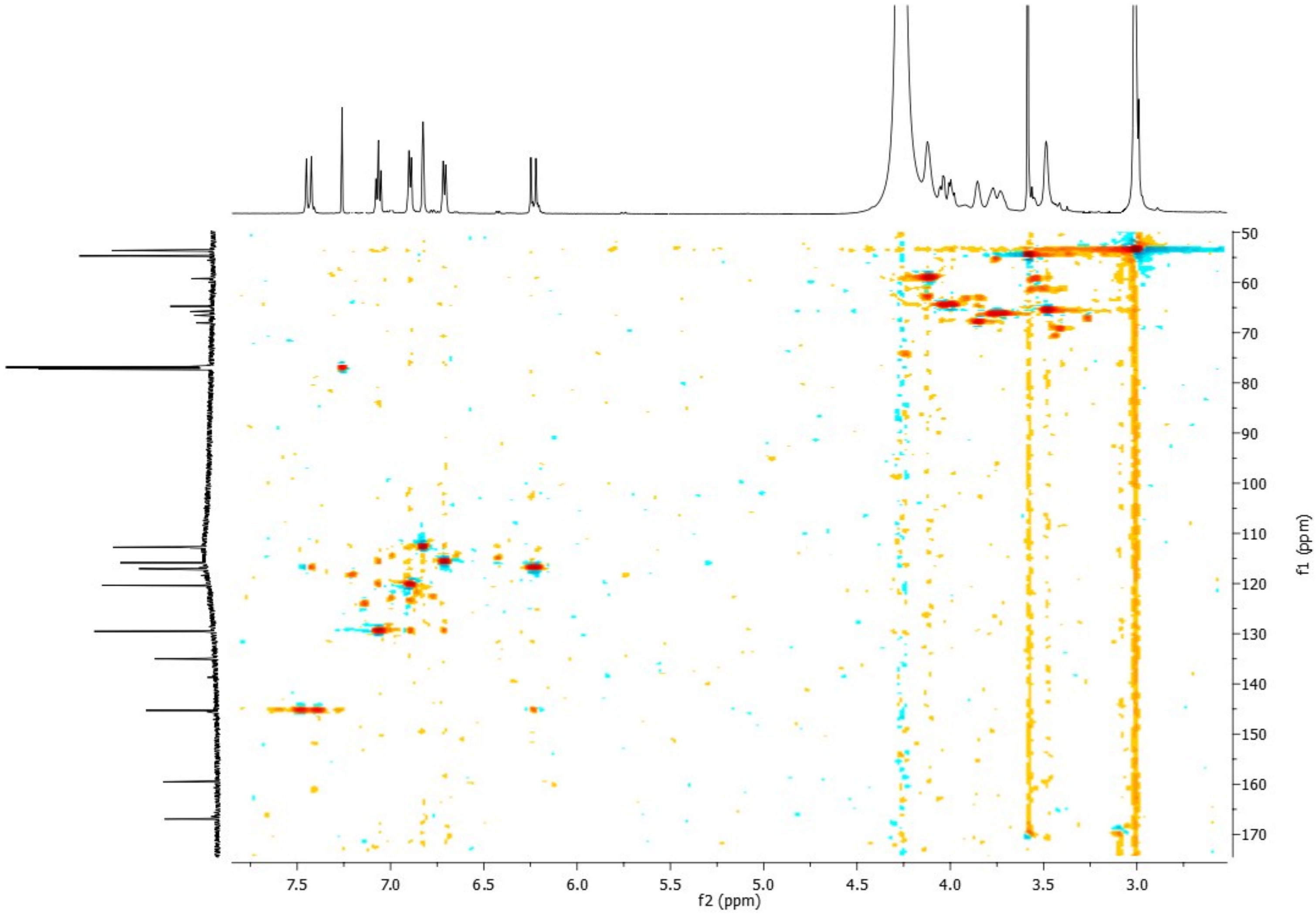

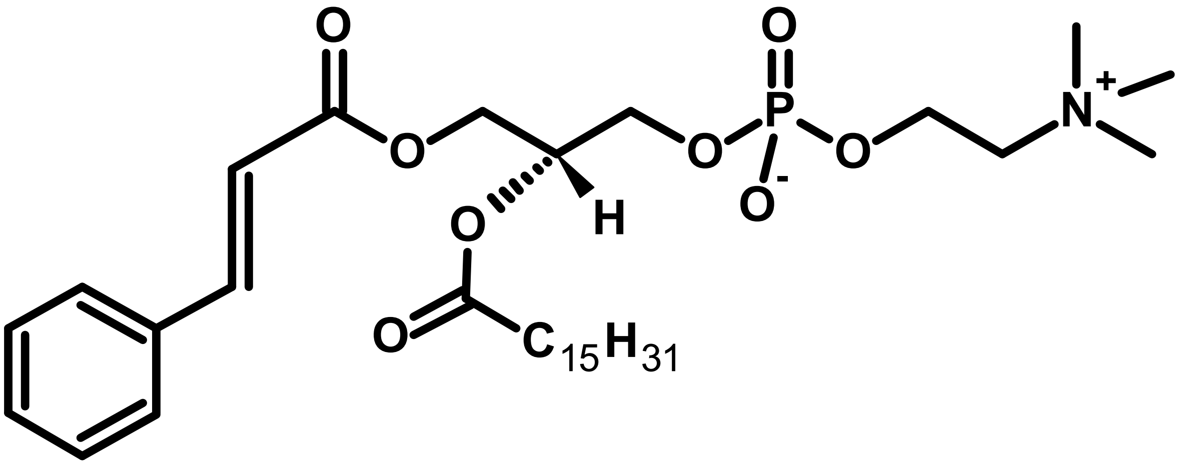

1-cinnamoyl-2-palmitoyl-*sn*-glycero-3-phosphocholine (**10a**)

Figure S31: ^{1}H NMR spectrum of 10a.

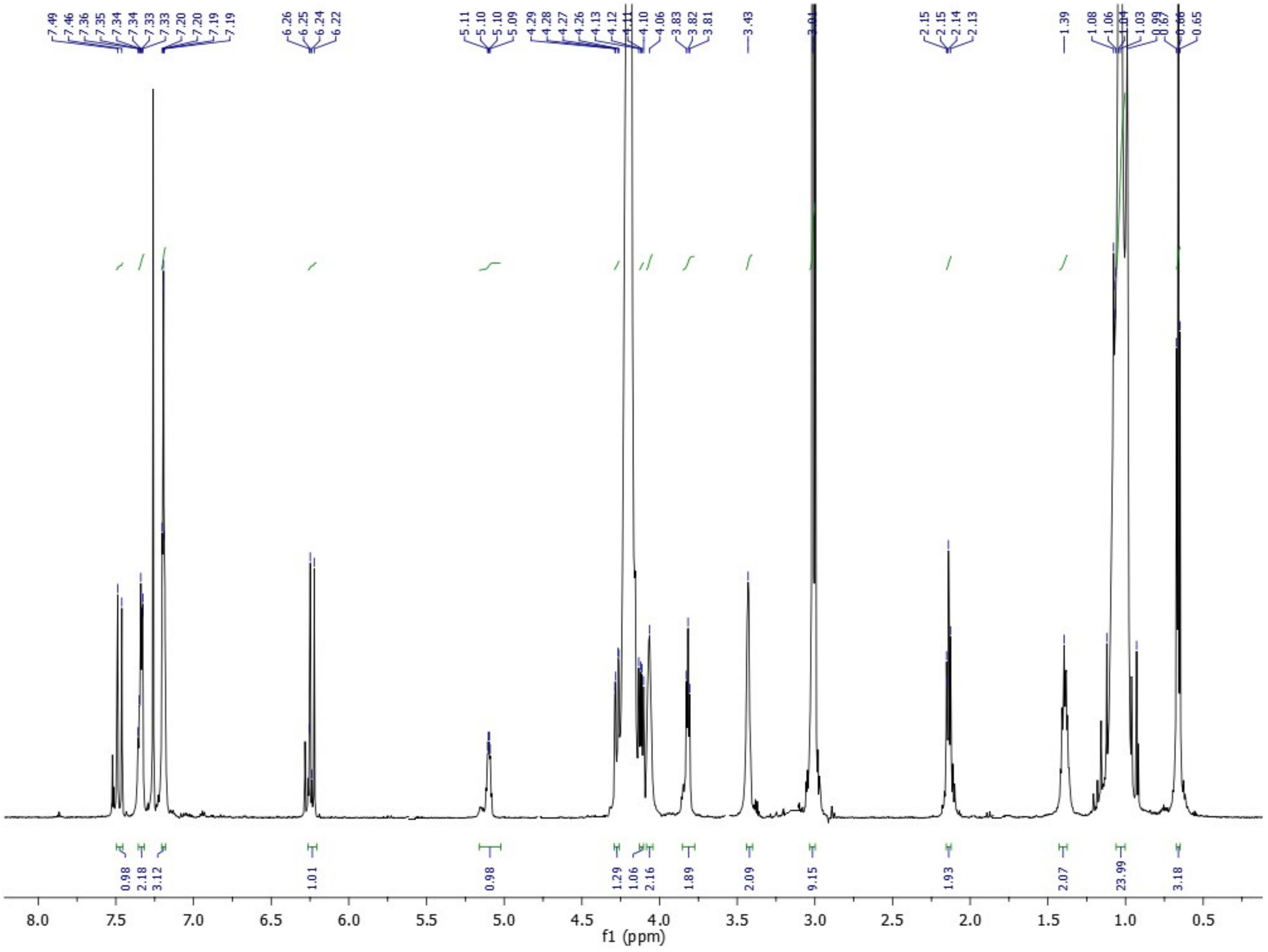

Figure S32: ^{13}C NMR spectrum of 10a.

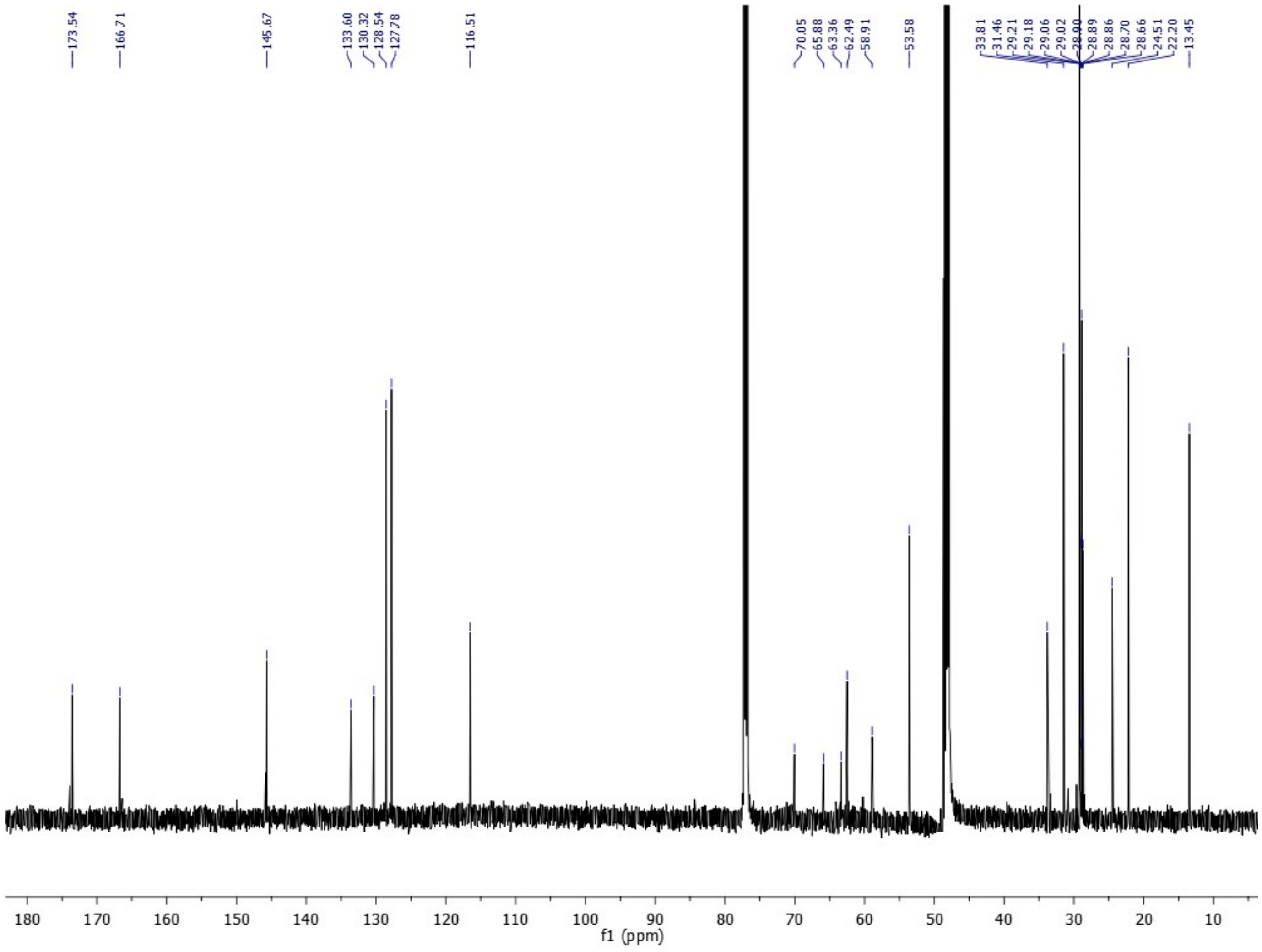

Figure S33: ^{31}P NMR spectrum of 10a.

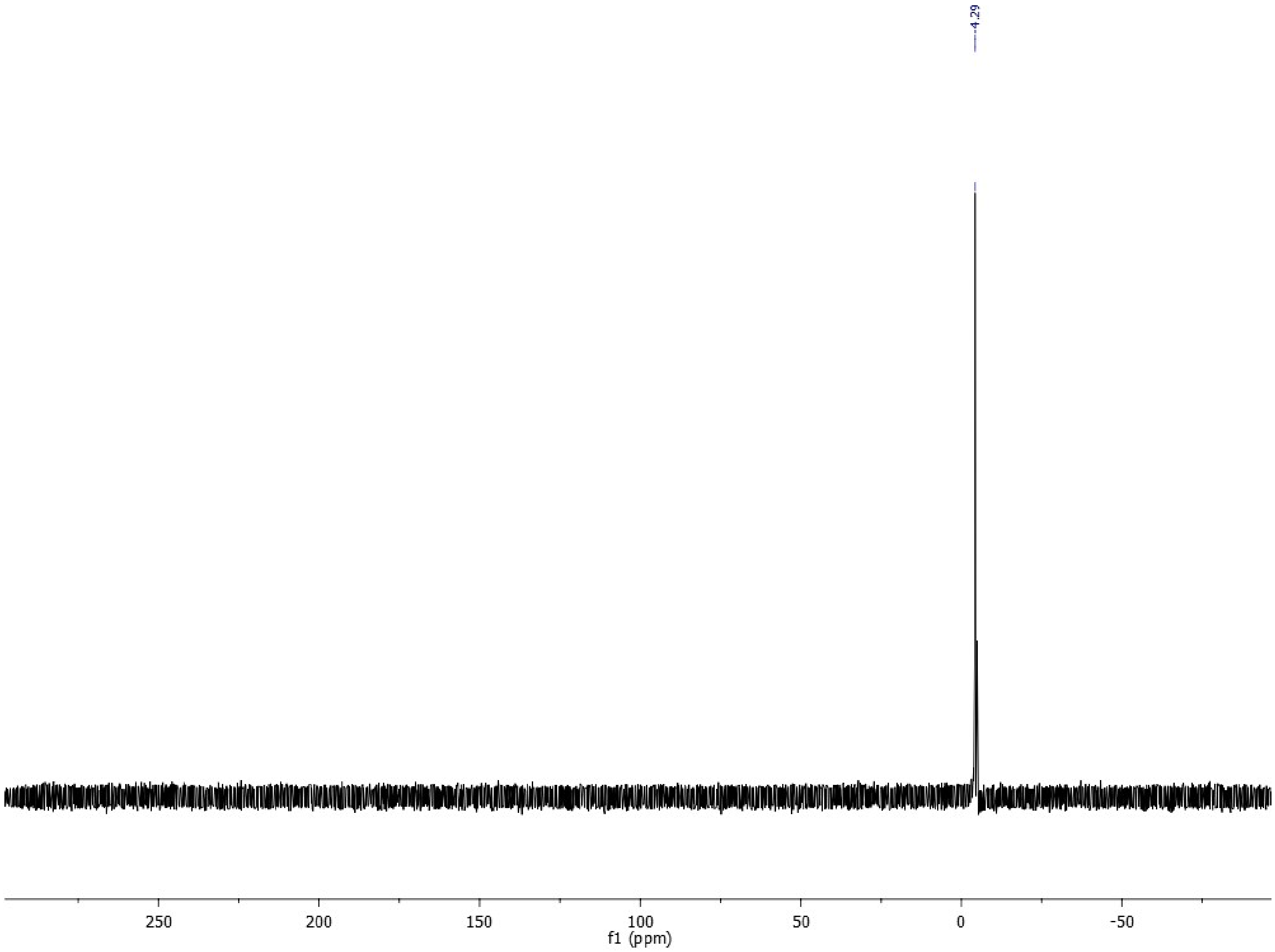

Figure S34: ^{1}H – ^{1}H COSY spectrum of 10a.

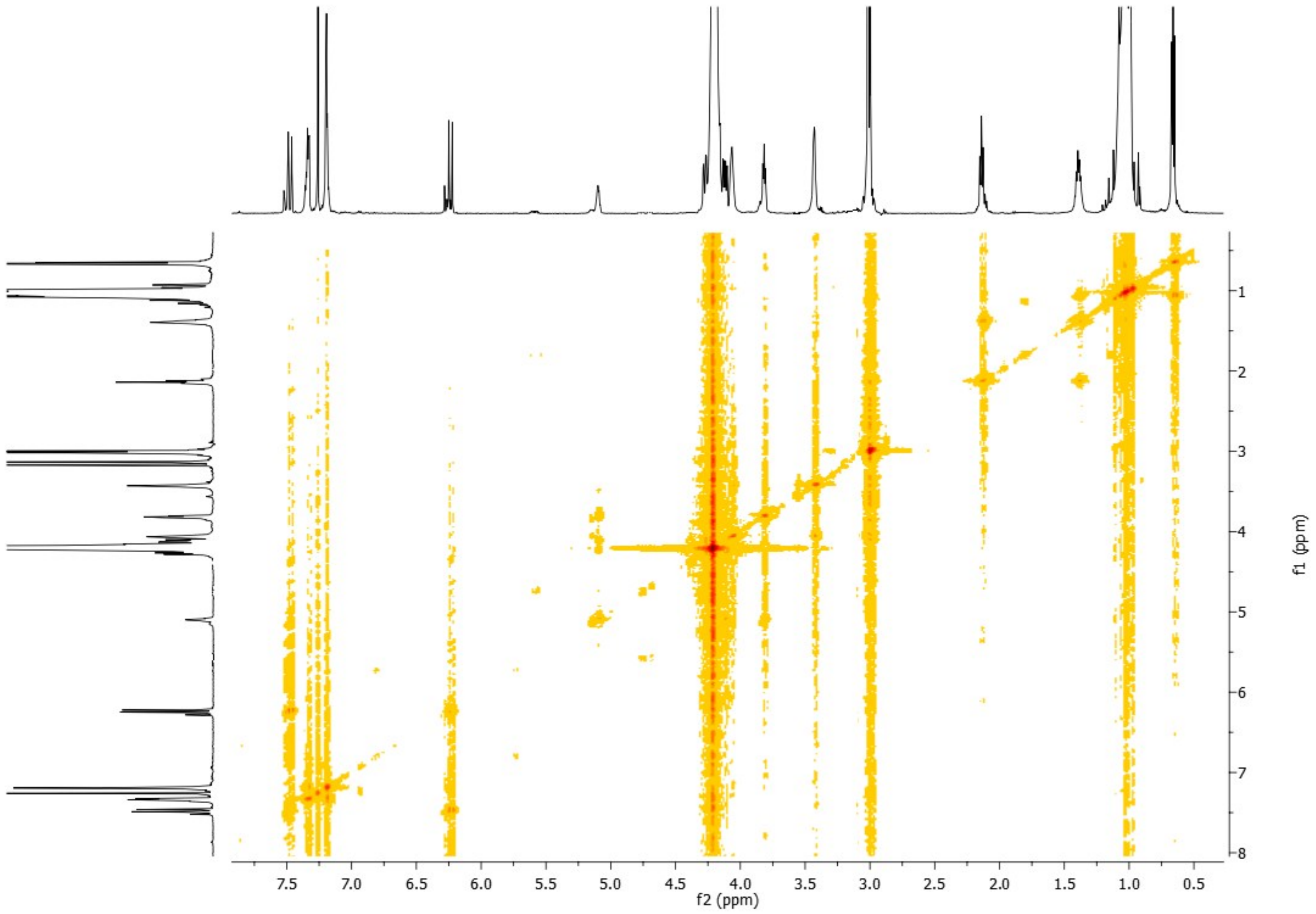

Figure S35: HSQC spectrum of 10a.

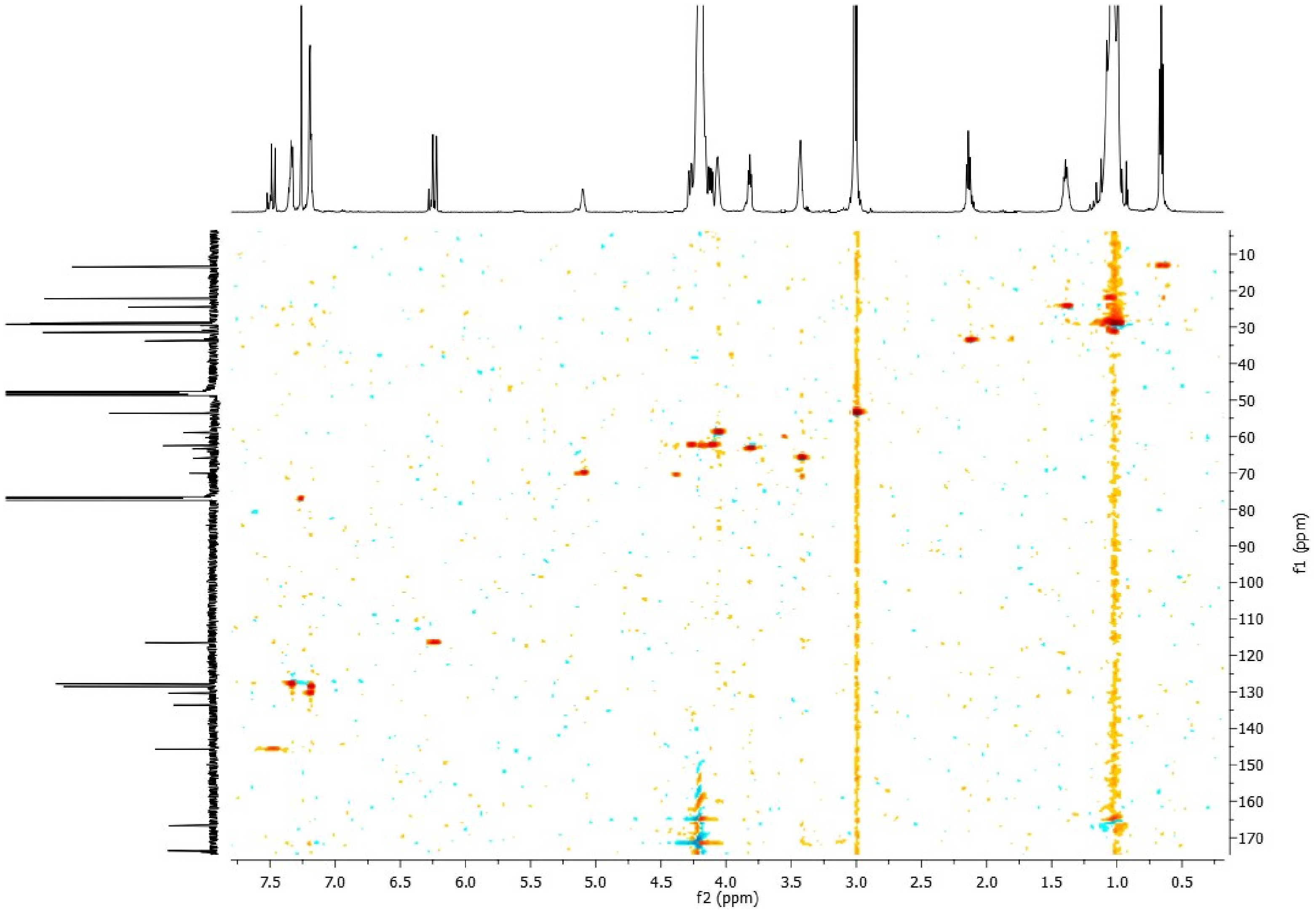

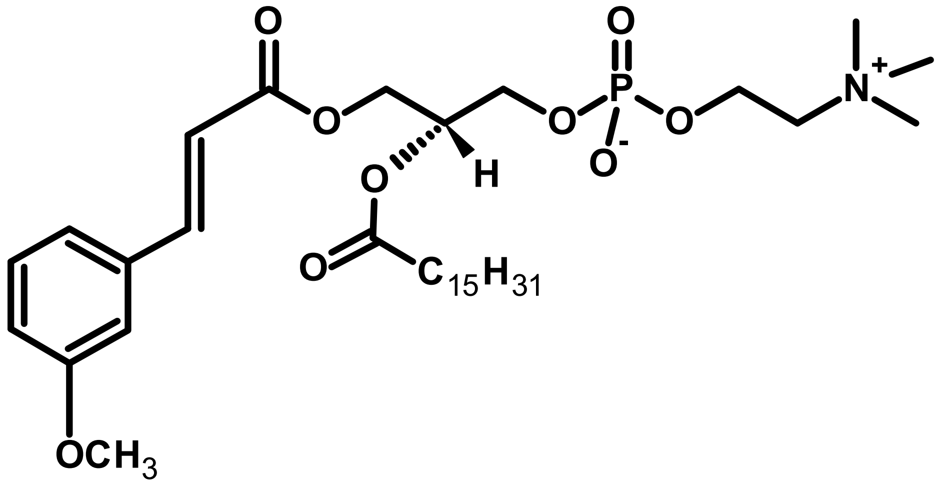

1-(3-methoxycinnamoyl)-2-palmitoyl-*sn*-glycero-3-phosphocholine (**10b**)

Figure S36: ^{1}H NMR spectrum of 10b.

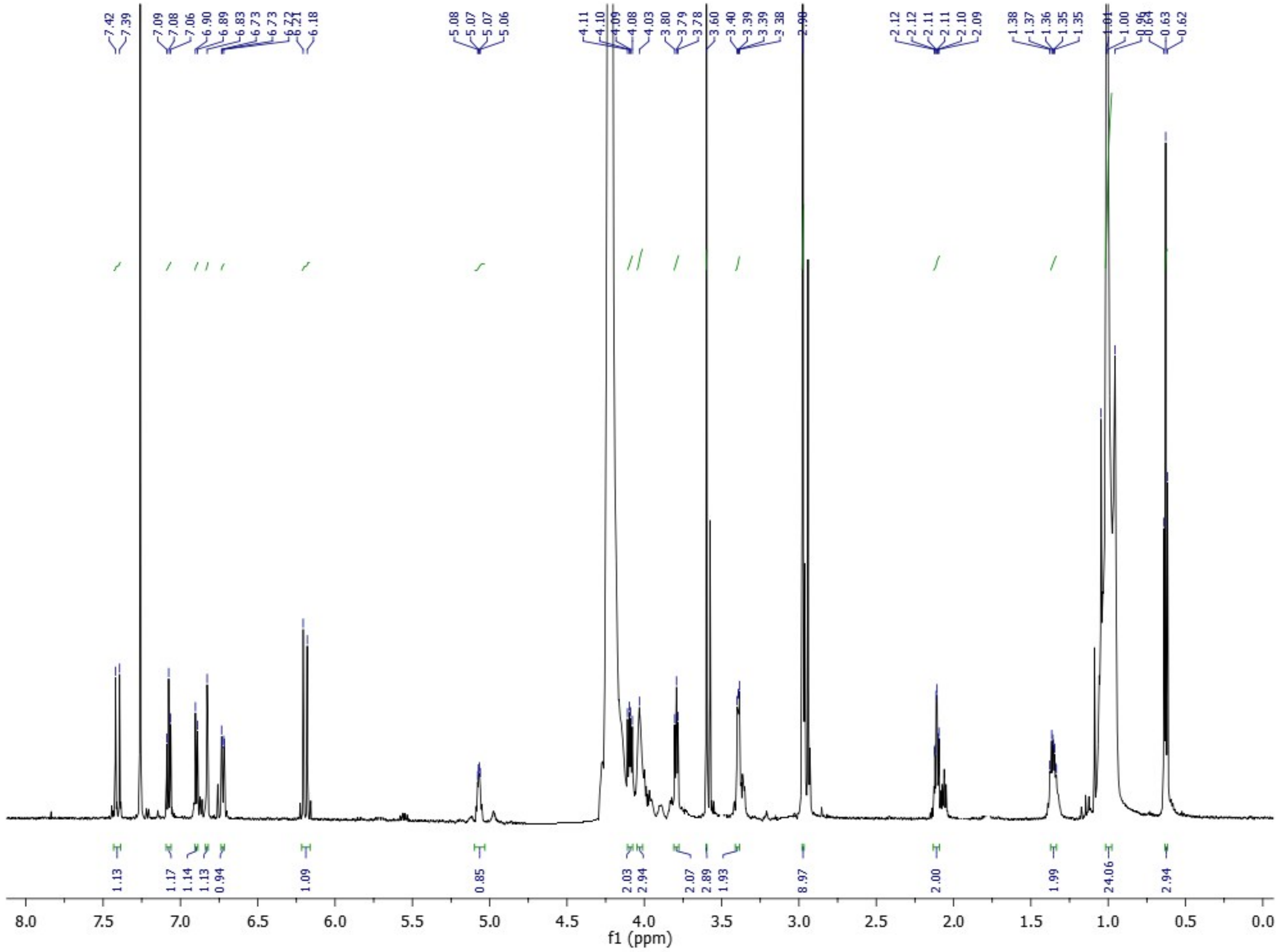

f1 (ppm)

Figure S37: ^{13}C NMR spectrum of 10b.

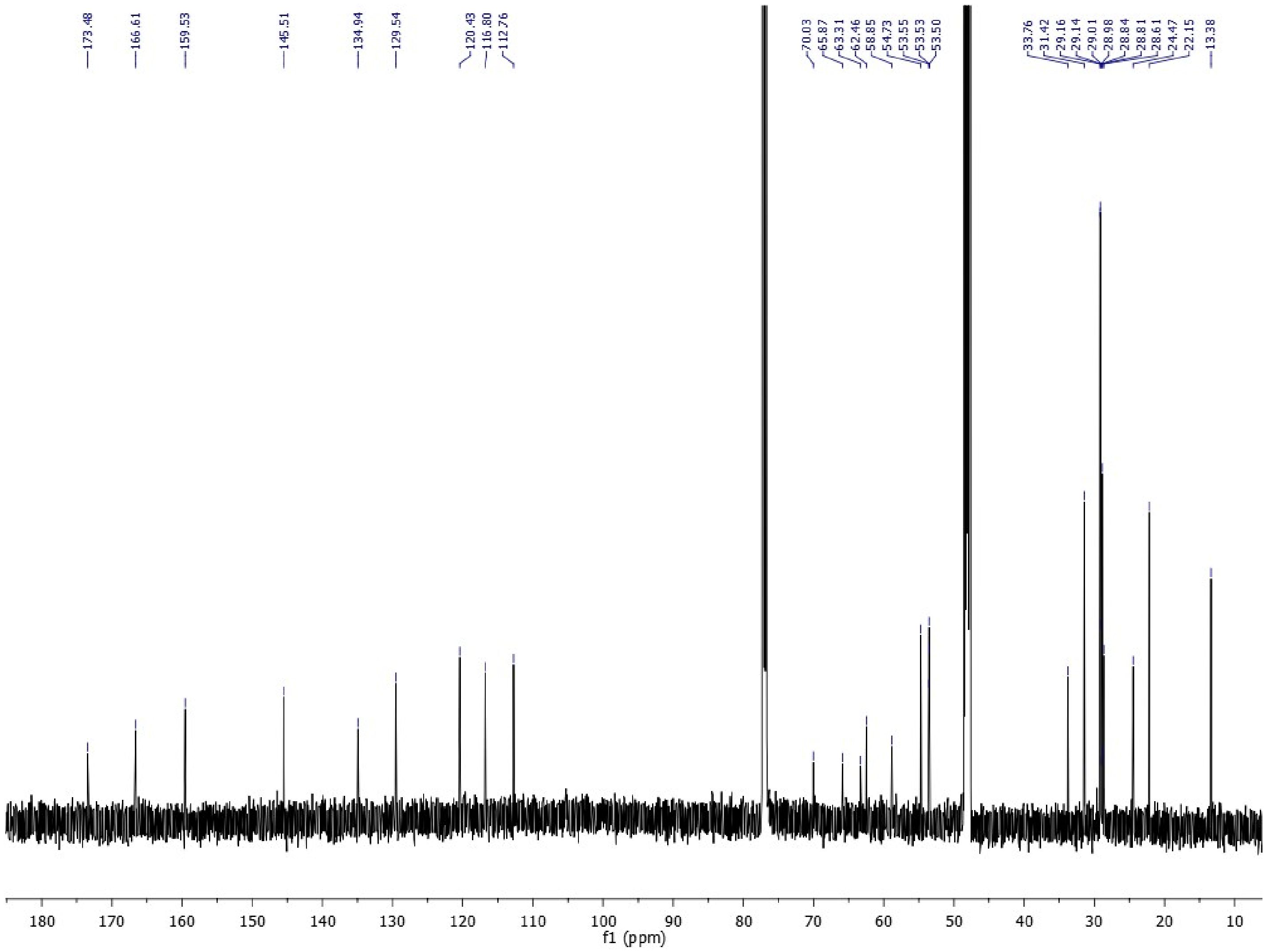

Figure S38: ^{31}P NMR spectrum of 10b.

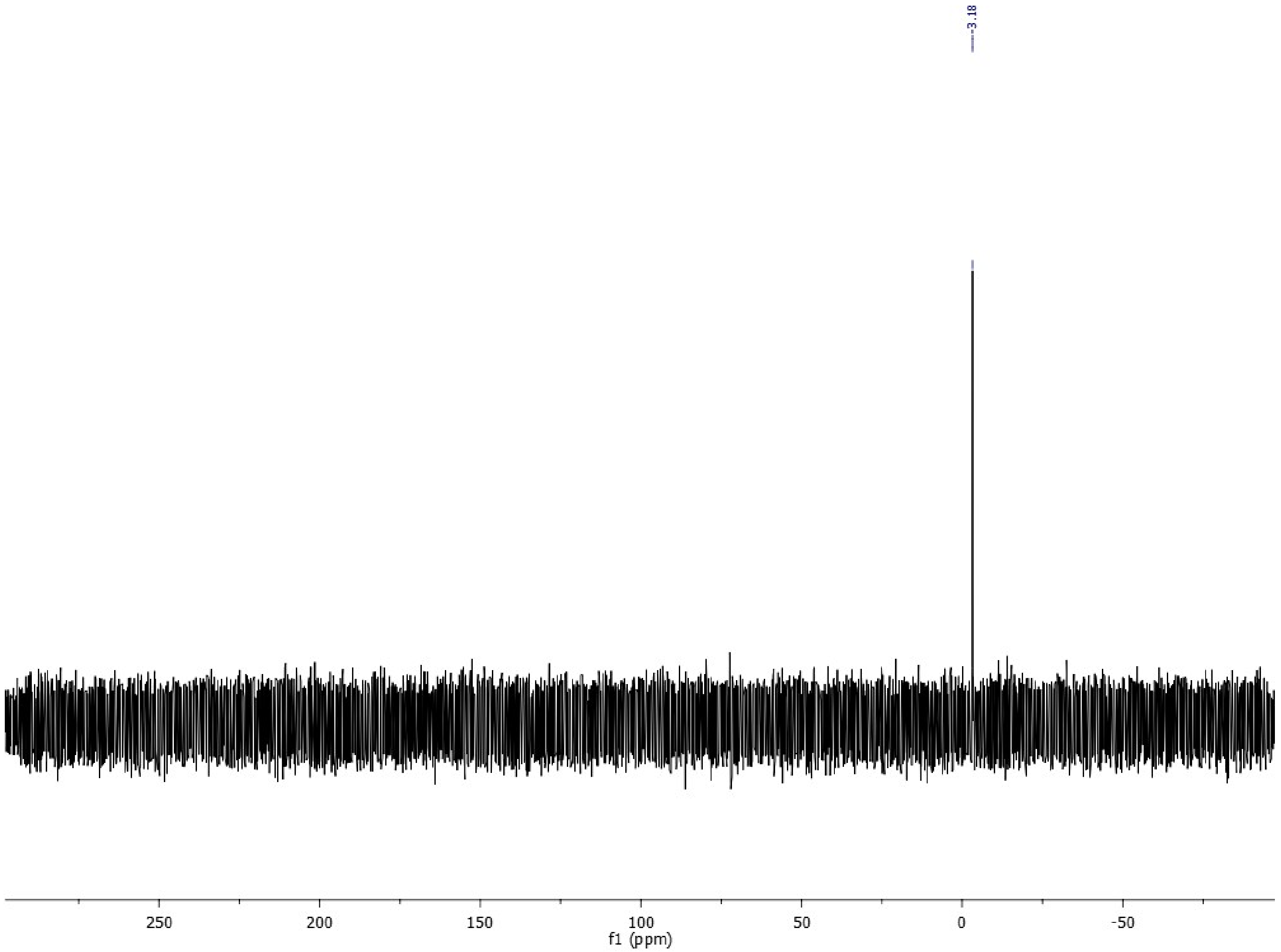

Figure S39: ^{1}H – ^{1}H COSY spectrum of 10b.

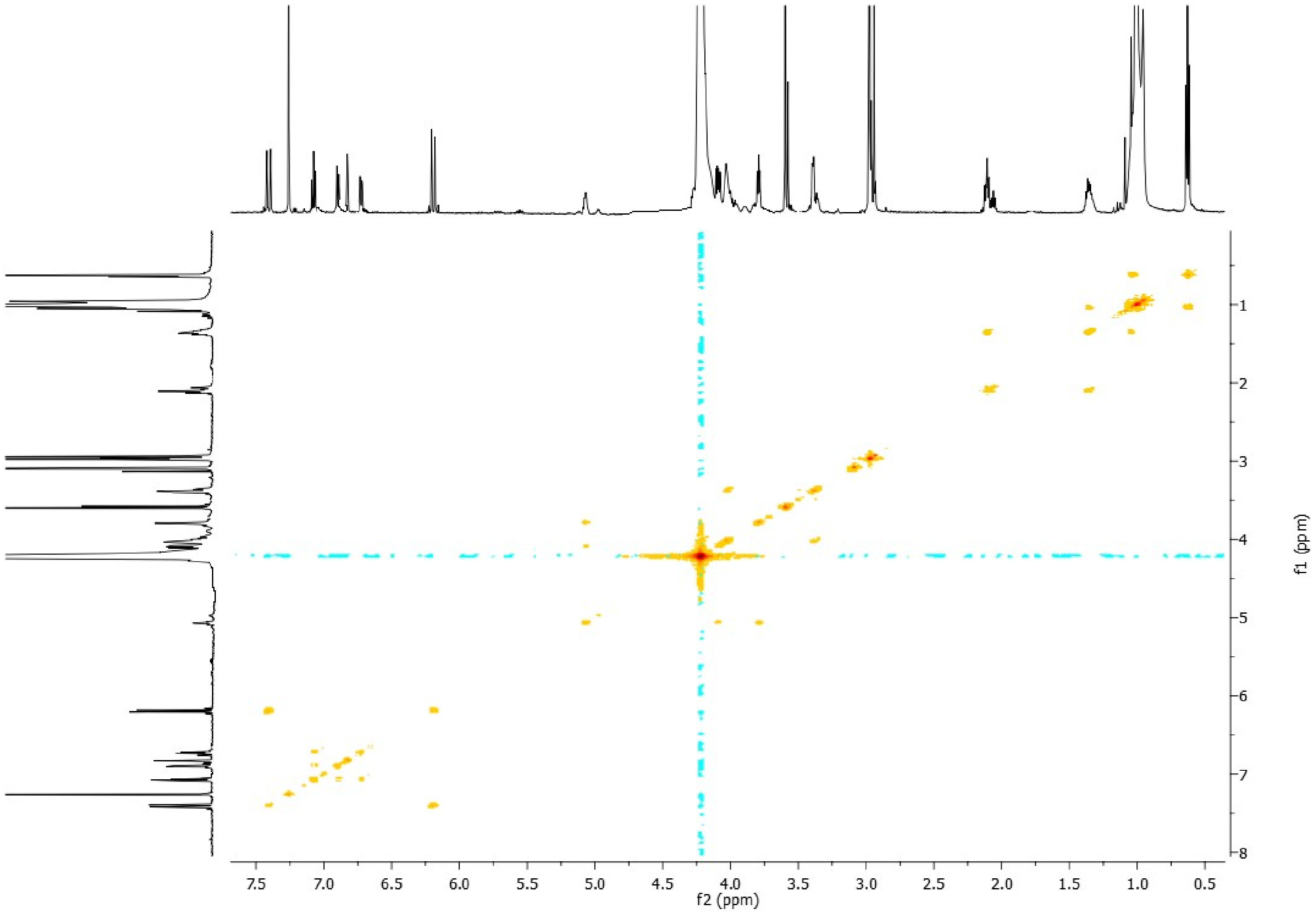

Figure S40: HSQC spectrum of 10b.

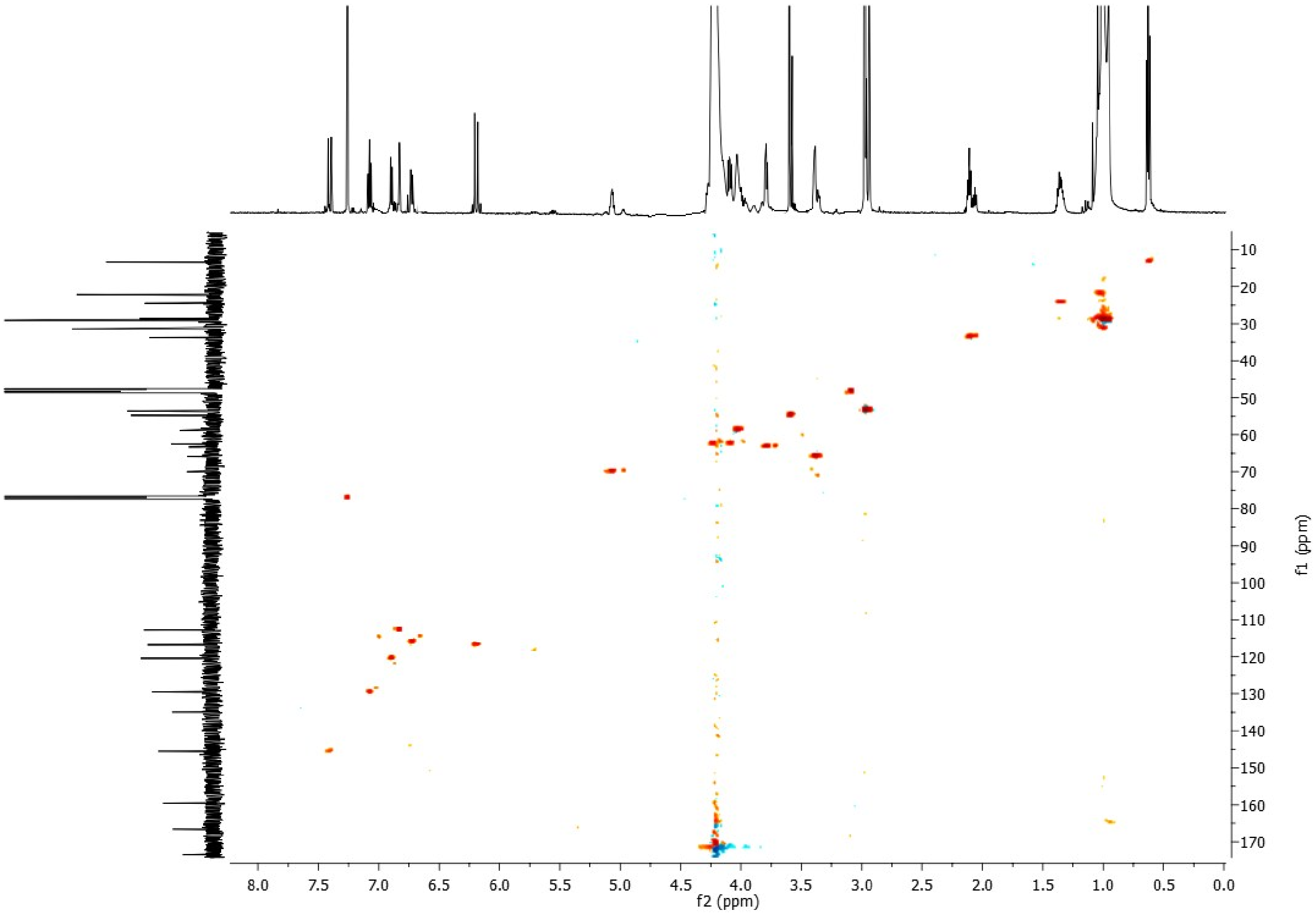

Supplement: RA-008-C8RA07002D-s001 [file RA-008-C8RA07002D-s001.pdf]
